# Urine Metabolomics of Gout Reveals the Dynamic Reprogramming and Non-Invasive Biomarkers of Disease Progression

**DOI:** 10.3390/metabo15090580

**Published:** 2025-08-29

**Authors:** Guizhen Zhu, Yuan Luo, Nan Su, Xiangyi Zheng, Zhusong Mei, Qiao Ye, Jie Peng, Peiyu An, Yangqian Song, Weina Luo, Hongxia Li, Guangyun Wang, Haitao Zhang

**Affiliations:** 1 Laboratory of Clinical Medicine, Air Force Medical Center, Air Force Medical University, People’s Liberation Army of China, Beijing 100142, China; 13121437811@163.com (G.Z.); ly-navy@163.com (Y.L.); zhengxy12345@163.com (X.Z.); mzsa@163.com (Z.M.); yeqiao333@163.com (Q.Y.); 19991205301@163.com (J.P.); yupeipyjj@sina.com (P.A.); 2 Military Medical Center, Air Force Medical Center, Air Force Medical University, People’s Liberation Army of China, Beijing 100142, China; 13911586246@139.com; 3 Rheumatology and Immunology Department, Air Force Medical Center, Air Force Medical University, People’s Liberation Army of China, Beijing 100142, China; m15168405793@163.com; 4 Cardiovascular Department, Air Force Medical Center, Air Force Medical University, People’s Liberation Army of China, Beijing 100142, China; luoweina_215@163.com

**Keywords:** hyperuricemia, gout, urine metabolomics, biomarkers, pathogenesis

## Abstract

**Background/Objectives:** Gout, a complex metabolic disorder of increasing global incidence, remains incompletely understood in its pathogenesis. Current diagnostic approaches exhibit significant limitations, including insufficient specificity and the requirement for invasive joint aspiration, highlighting the need for non-invasive, sensitive biomarkers for early detection. **Methods:** Urine metabolites were extracted from 28 healthy controls, 13 asymptomatic hyperuricemia (HUA) patients, and 29 acute gouty arthritis (AGA) patients. The extracted metabolites were analyzed by UHPLC-MS/MS for untargeted metabolomics. Differential metabolites were screened by partial least squares discriminant analysis (PLS-DA) and volcano plot analysis. Pathway analysis determined the core disorder pathway of gout progression. **Results:** A total of 278 differential metabolites associated with gout progression were identified. The most pronounced metabolic alterations were observed between the AGA and control groups, indicative of substantial metabolic reprogramming during disease transition. Metabolic pathway analysis revealed four significantly dysregulated pathways: histidine metabolism, nicotinate and nicotinamide metabolism, phenylalanine metabolism, and tyrosine metabolism. Receiver operating characteristic (ROC) curve analysis revealed that three urine markers with high diagnostic efficacy—oxoamide, 3-methylindole, and palmitic acid—exhibited progressive alterations across the disease continuum. **Conclusions:** This metabolomics study identified core regulatory metabolites and newly discovered metabolic pathways underlying gout pathogenesis, along with novel urinary biomarkers capable of predicting HUA-to-AGA progression. The aberrant levels of key metabolites in the disordered pathway implicate neuroimmune dysregulation, energy metabolism disruption, and oxidative stress in gout pathogenesis. These findings provide new foundations and strategies for the daily monitoring and prevention of gout.

## 1. Introduction

The incidence of gout, a common metabolic disease, has significantly increased worldwide in recent years [1,2,3]. During acute gouty arthritis (AGA), patients often experience joint pain, redness, fever, and other symptoms. These symptoms cause difficulty in walking, greatly reducing freedom of movement and the ability to perform daily activities, which can seriously affect patients’ quality of life [4]. Moreover, AGA symptoms can lead to a series of complications, such as joint dysfunction, kidney damage, and cardiovascular disease [5,6]. Therefore, it is critical to explore the pathogenesis of gout and develop effective early diagnostic methods for improving patients’ physical and mental health and quality of life.

Although some achievements have been made in medical research exploring the pathogenesis of gout, many key links remain unclear. Research has shown that the deposition of urate crystals in joints and surrounding tissues is the key starting factor for the induction of gout-related inflammatory reactions [7,8]. Immune cells can recognize urate crystals. Subsequently, inflammatory corpuscles are activated, causing inflammatory cells to release numerous inflammatory factors, such as interleukin-1β, interleukin-6, and tumor necrosis factor-α, resulting in local inflammation and joint injury [9,10,11]. However, hyperuricemia (HUA) is not the same as gout [12,13]. Clinically, only some patients with HUA develop gout [14]. Furthermore, some AGA patients still experience seizures even if their blood uric acid level is well-controlled [15]. This phenomenon shows that the pathogenesis of gout is extremely complicated. In addition to the abnormal metabolism of uric acid, gout may also be closely related to genetic and environmental factors, imbalances in the intestinal microbial community, and disorders of various metabolic pathways in the body. For example, changes in several metabolic pathways may affect uric acid transport, metabolic processes, and the regulation of the inflammatory response.

Early and accurate diagnosis is critical for effective gout treatment and prognosis improvement. Gout is clinically diagnosed by blood uric acid level detection, joint fluid puncture, and clinical manifestations [16,17,18]. Analysis of the serum uric acid level is one of the most commonly used diagnostic methods. However, during a gout attack, the blood uric acid level is easily disturbed by many factors. Some patients have normal blood uric acid levels, increasing the missed diagnosis risk. The joint fluid puncture is the gold standard for diagnosing gout; the diagnosis can be made by detecting urate crystals in joint fluid. However, this method is an invasive operation, which will make patients suffer physical pain. The operation process of joint fluid puncture is complex and requires high technology and equipment, so it is difficult to use for early screening and routine examination on a large scale. In addition, the clinical manifestations of gout are subjective and atypical. In particular, the initial symptoms may be insignificant or similar to those of other joint diseases, which may easily lead to misdiagnosis. Therefore, it is urgent to explore sensitive, specific, and non-invasive early warning biomarkers to realize early and accurate diagnosis of gout, which is highly important for clinical diagnostics and treatment.

Nontargeted urine metabolomics, as a key branch of metabolomics, provides new hope and opportunities for the in-depth exploration of gout pathogenesis and the identification of early warning biomarkers [19,20,21,22]. Urine, which contains many endogenous small-molecule metabolites, is an important excretion route for human metabolic waste [23]. These metabolites are products of the body’s metabolism, and changes in their types and contents can accurately reflect the body’s metabolic state and pathophysiological process in real time [24,25,26]. In addition, urine samples have the advantages of convenient collection, non-invasiveness, and strong repeatability. Urine is convenient for large-scale sample collection and long-term dynamic monitoring [27]. With the help of nuclear magnetic resonance, ultrahigh-performance liquid chromatography–mass spectrometry (UHPLC-MS), and gas chromatography–mass spectrometry, the metabolites in urine can be analyzed unbiasedly, omnidirectionally, and accurately [28,29,30]. Using nontargeted urine metabolomics technology, we can perform an in-depth analysis of the mechanisms of the metabolic disorder underlying gout pathogenesis and accurately locate the key metabolic pathways and metabolites closely related to gout.

This study employed UHPLC-MS/MS to analyze the characteristics of urine metabolomics in healthy people (control), asymptomatic HUA patients, and AGA patients. The objectives were as follows: (1) to explore the differences of urine metabolomics among the three groups; (2) to elucidate previously unrecognized metabolic pathways implicated in gout pathogenesis; and (3) to identify highly specific and sensitive urinary biomarkers for the early detection of gout.

## 2. Materials and Methods

### 2.1. Participants and Study Design

**Figure 1:**
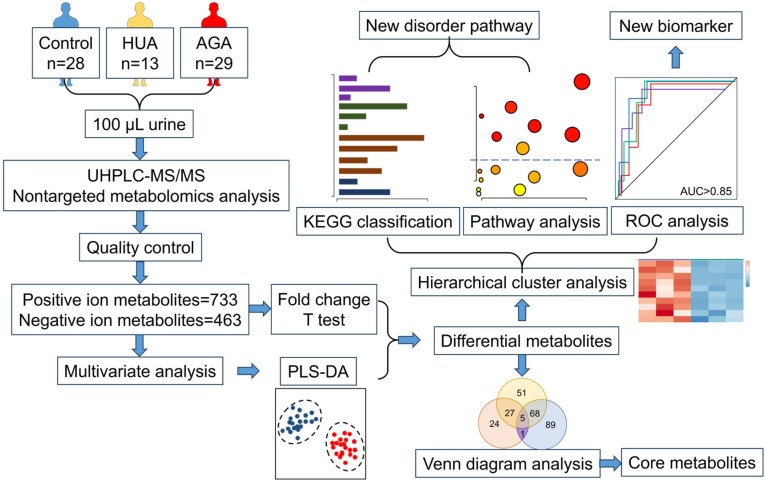
Research design and data analysis workflow. HUA, hyperuricemia; AGA, acute gouty arthritis; UHPLC-MS/MS, ultrahigh-performance liquid chromatography–mass spectrometry; PLS-DA, partial least squares discriminant analysis; KEGG, Kyoto Encyclopedia of Genes and Genomes; ROC, receiver operating characteristic.

Urine samples were collected from 69 individuals in the Department of Rheumatology and Immunology, Air Force Medical Center. Among these 69 individuals, 28 were healthy people, 13 were asymptomatic HUA patients, and 29 were AGA patients. HUA and AGA patients had no other diseases and had not been treated clinically. Urine samples were collected at the early stage of the disease. Clinicians classified all urine according to the diagnostic results. Fresh midstream urine samples were collected from each participant in the morning. The urine samples were centrifuged at 4 °C and 1000× *g* for 15 min to remove cells and cell debris. The supernatant was divided into two 2 mL frozen tubes. After quick freezing in liquid nitrogen for 15 min, the samples were stored at −80 °C for testing. The workflow of the research design and data analysis is shown in Figure 1. This study was approved by the ethics committee of the Air Force Medical Center and conducted in compliance with the 1964 Helsinki Declaration. All participants provided written informed consent.

### 2.2. Sample Treatment

A total of 100 µL of each urine sample was placed in an EP tube. Next, 400 μL of 80% methanol aqueous solution was added. The mixture was vortexed and allowed to stand in an ice bath for 5 min. The mixture was subsequently centrifuged at 15,000× *g* for 20 min at 4 °C. An aliquot of the supernatant was diluted with water until the methanol content reached 53%. The diluted solution was centrifuged again according to the above conditions, and the supernatant was collected for UHPLC-MS/MS analysis.

### 2.3. UHPLC-MS/MS

A ThermoFisher Vanquish UHPLC instrument equipped with a Hypes IL Gold column (100 × 2.1 mm, 1.9 μm) was used to separate the urine samples. Mobile phase A was an aqueous solution containing 0.1% formic acid. Mobile phase B was methanol. The elution method was as follows: 0–1.5 min, 2–2% B; 1.5–3 min, 2–85% B; 3–10 min, 85–100% B; 10–10.1 min, 100–2% B; and 10.1–12 min, 2–2% B. The flow rate was 0.2 mL/min. The injection volume was 10 µL. The column temperature was stable at 40 °C.

Detection was performed in positive and negative ion modes using the heated electrospray ion source of a Q Exactive HF/Q Exactive HF-X mass spectrometer. The sheath gas flow rate was 35 psi. The flow rate of auxiliary gas was 10 L/min. The spray voltage was 3.5 kV. The temperature of the ion transport tube was 320 °C. The S-lens RF level of ion introduction was 60. The auxiliary gas heater temperature was 350 °C. In Full scan mode, the scanning range was 100–1500 Da, the resolution was 60,000 fwhm, the AGC was 3 × 10^6^, and the maximum injection time was 100 ms. The MS/MS spectra of the top 20 ions with peak intensity were obtained by data-dependent scans. The resolution was 15,000 fwhm, AGC was 2 × 10^5^, the maximum injection time was 25 ms, and the dynamic exclusion time was 10 s.

### 2.4. Data Preprocessing and Metabolite Identification

The data obtained from UHPLC-MS/MS detection were imported into the Compound Discoverer 3.3 database search software to screen each metabolite’s retention time and mass–charge ratio. The first quality control (QC) sample was used to correct the peak area, making the identification result more accurate. Peak extraction was performed with a mass deviation of 5 ppm, a signal intensity deviation threshold of 30%, and a minimum signal intensity of 100,000, while incorporating adduct ion forms and excluding isotopic peaks. The peak area was quantified, and the target ions were integrated. The molecular formula was subsequently predicted using molecular ion peaks and fragment ions and compared with the mzCloud, mzVault, and Masslist databases. A blank sample was used to remove background ions. The original quantitative results were standardized according to the following formula to obtain the relative peak area: original quantitative value of samples/(total quantitative value of metabolites in samples/total quantitative value of metabolites in QC1 samples). The compounds with relative peak area CVs greater than 30% in the QC samples were filtered out. Finally, the metabolites were identified and quantified.

### 2.5. Statistical Analysis

The metabolites identified above were annotated using the KEGG, HMDB, and LIPID MAPS databases. The MetaX v2.87 software was used to convert the data, and then principal component analysis (PCA) and PLS-DA were used to obtain the variable importance in projection (VIP) value of each metabolite. The statistical significance (*p* value) of the metabolites was calculated using a t-test. The fold change (FC) of metabolites between the two groups was calculated. The differential metabolites were screened on the basis of VIP > 1, *p* < 0.05, and FC > 1.2 or FC < 0.83. A volcano map was constructed with R-bag ggplot2 to show the screening results of the differential metabolites. Through hierarchical cluster analysis of differential metabolites obtained from the metaboanalyst website, the differences in metabolic expression patterns between groups and within groups were obtained. The website was used to analyze the pathways of different metabolites and construct a bubble diagram of the enriched metabolic pathways.

## 3. Results

### 3.1. Clinical Characteristics of the Participants

The clinical characteristics of the study participants are presented in Table 1. The serum urate acid levels were significantly elevated in both AGA patients [median (IQR): 465.0 (336.0–696.0) μmol/L] and HUA patients [median (IQR): 477.1 (431.8–567.9) μmol/L] compared to controls [median (IQR): 342.0 (231.6–427.3) μmol/L]. Notably, the serum uric acid levels of AGA and HUA patients are similar. Although participants with other metabolic diseases were excluded to minimize confounding, significant differences were observed between the AGA and control groups for age, body mass index (BMI), smoking status, alcohol consumption, daily sleep duration, alanine aminotransferase (ALT), glucose, and triglyceride levels. The HUA patients also exhibited significant differences relative to the controls in BMI and blood urea nitrogen levels. However, except for age and smoking status, there were no significant differences in these parameters between AGA and HUA patients.

### 3.2. Quality Control of Metabolomics Data Using UHPLC-MS/MS

Metabolic groups are easily disturbed by external factors and change rapidly. In particular, the sample size is large and sample detection can be a lengthy process. Whether the stability of the instrument and the signal response are normal in the process of metabolite detection is particularly important. Therefore, data QC is necessary to obtain stable and accurate metabolomic results. We prepared QC samples by mixing control, HUA, and AGA samples and inserted them into the sample analysis sequence for detection.

**Figure 2:**
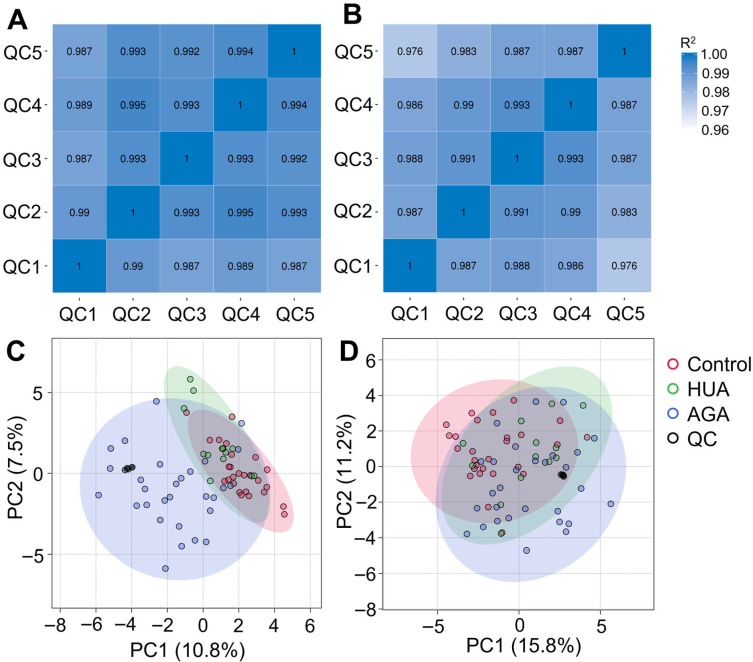
(**A**,**B**) Correlation analysis of QC samples in UHPLC-MS/MS positive and negative ion modes. (**C**,**D**) PCA of total samples in UHPLC-MS/MS positive and negative ion modes. HUA, hyperuricemia; AGA, acute gouty arthritis; QC, quality control; PC, principal component.

QC sample correlation analysis and population sample PCA were used to control the data quality. The Pearson correlation coefficient between the QC samples was calculated according to the relative quantitative values of the metabolites. The R2 value between the QC samples was greater than 0.97 in both positive and negative ion modes (Figure 2A,B). That is, the correlation between the QC samples was high. We further analyzed the peaks extracted from all the control, HUA, AGA, and QC samples using PCA, revealing that the QC samples’ distribution was clustered (Figure 2C,D). The above results show that the instrument state and the detection method were stable throughout the entire detection process and that the experimental data were accurate.

### 3.3. Metabolite Analysis

We used positive and negative ion ionization modes to perform UHPLC-MS/MS to identify metabolites to the greatest extent possible. A total of 727 and 362 metabolites were identified in positive and negative ion mode, respectively (Tables S1 and S2). Organic compounds, organic acids and their derivatives, and lipids and lipid-like molecules accounted for 23.42%, 22.30%, and 21.85%, respectively, in the positive ion mode (Figure S1A). Lipids and lipid-like molecules accounted for 27.21%, and organic acids and their derivatives accounted for 25.90% in the negative ion mode (Figure S1B).

The identified metabolites were annotated using the KEGG, HMDB, and LIPID MAPS databases to understand the functional characteristics and classification of different metabolites (Figures S2–S4). Pathway annotation using the KEGG database revealed that 1089 metabolites were mainly involved in the amino acid metabolism pathway. In the HMDB and LIPID MAPS database annotation classifications, the identified metabolites were mainly organic acids and their derivatives and fatty acids and conjugates. Notably, HUA patients exhibited significantly lower urinary uric acid levels compared to healthy controls. In contrast, AGA patients showed significantly higher urinary uric acid levels than HUA patients, with levels comparable to healthy controls (Figure S5A). These findings suggest that, unlike serum urate levels, urinary uric acid measurement may not serve as a reliable biomarker for gout diagnosis.

### 3.4. Screening of Differential Metabolites

**Figure 3:**
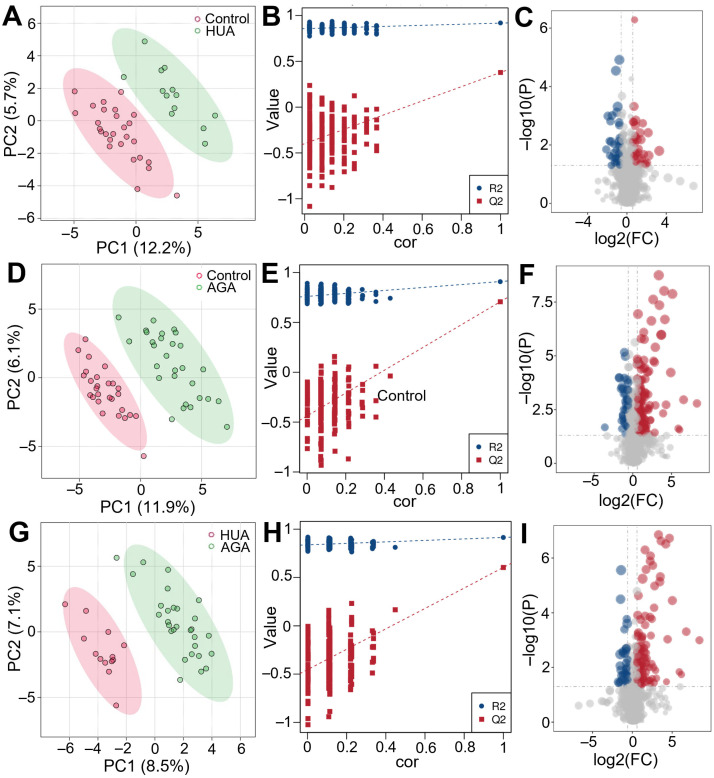
(**A**) PLS-DA analysis, (**B**) model evaluation, and (**C**) volcano plot analysis of HUA patients vs. controls. (**D**) PLS-DA analysis, (**E**) model evaluation, and (**F**) volcano plot analysis of AGA patients vs. controls. (**G**) PLS-DA analysis, (**H**) model evaluation, and (**I**) volcano plot analysis of AGA vs. HUA patients. PC, principal component; FC, fold change.

PLS-DA is a supervised statistical method of discriminant analysis that uses partial least squares regression to establish a relationship model between the expression of metabolites and sample categories to predict sample categories. PLS-DA analysis was conducted on the positive and negative combined metabolic spectra of the HUA vs. control, AGA vs. control, and AGA vs. HUA groups (Figure 3A,D,G). The three comparative pairs all clearly exhibited intragroup aggregation and intergroup dispersion, demonstrating that the samples had good parallelism and that the metabolic spectra of the two groups were obviously different. The results showed that the metabolic process in healthy people that developed into AGA changed significantly. The interpretation rates (R2Y) of the PLS-DA models of the three comparative pairs were 0.92, 0.91, and 0.91, and the prediction abilities (Q2Y) of the models were 0.38, 0.71, and 0.6. The model we constructed showed strong differentiation capabilities between the control, HUA, and AGA samples, demonstrating that the model is stable and reliable.

We randomly scrambled the grouping labels of each sample before modeling and forecasting. Each model corresponded to a set of R2 and Q2 values. According to the Q2 and R2 values after 200 perturbations and modeling, regression lines were obtained to check whether the PLS-DA model was overfitted (Figure 3B,E,H). The R2 values of the three comparison pairs of the PLS-DA model were greater than the Q2 values, and the intercepts of the Q2 regression line and *Y*-axis were less than zero. Therefore, the PLS-DA model we established had not been fitted. That is, the model can describe the sample well and can be used as a prerequisite for finding the biomarker population of the model.

The contribution degree (VIP value) of each metabolite obtained from the PLS-DA model combined with the fold change (FC) and the *p* value obtained from the t test were used to screen the differential metabolites in the HUA vs. control, AGA vs. control, and AGA vs. HUA groups. The thresholds were set as VIP > 1, FC > 1.5 or FC < 0.67, and *p* < 0.05. The overall distribution of differential metabolites is visually displayed in a volcano map. The abscissa represents the different folds of metabolites in different groups, and the ordinate represents the difference in significance level. Each point in the volcano map represents a metabolite (Figure 3C,F,I). The red dots represent metabolites whose expression is significantly upregulated, and the blue dots represent metabolites whose expression is significantly downregulated. The size of the dot represents the VIP value. A total of 70, 175, and 147 differential metabolites were screened from the three comparative pairs (Tables S3–S5), resulting in a total of 277. In HUA patients, 34 metabolites demonstrated upregulated expression, and 36 demonstrated downregulated expression. In AGA patients, 136 metabolites demonstrated upregulated expression, and 39 demonstrated downregulated expression. In the AGA vs. HUA group, 111 metabolites demonstrated upregulated expression, and 36 demonstrated downregulated expression. The differential metabolites of AGA vs. control were significantly more than those of other groups, indicating that AGA had more significant metabolic reprogramming than the control.

### 3.5. Differential Metabolite Analysis

**Figure 4:**
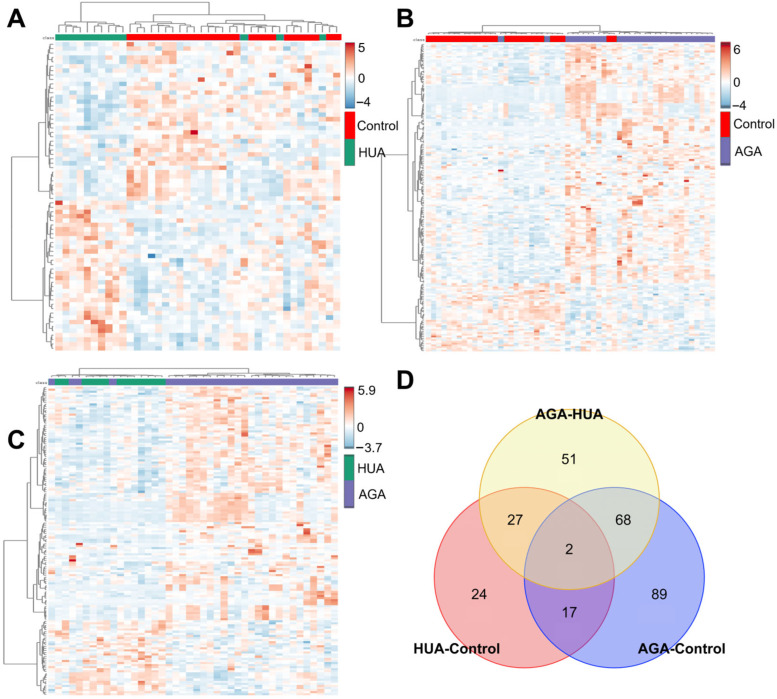
Heatmap analysis and Venn diagram analysis results (**D**) of differential metabolites of the (**A**) HUA vs. control, (**B**) AGA vs. control, and (**C**) AGA vs. HUA groups. HUA, hyperuricemia; AGA, acute gouty arthritis.

To study the change trend of the abovementioned screened differential metabolites in the control, HUA, and AGA groups, hierarchical cluster analysis was performed. The differences in metabolic expression patterns between and within groups were determined (Figure 4A,B). The abscissa represents the sample name, and the ordinate represents the differential metabolites. The color from blue to red indicates that the expression abundance of metabolites ranges from low to high. That is, red indicates that the expression abundance of different metabolites is relatively high. According to the heatmap results, the metabolites with the same change trend were clustered, i.e., their metabolic patterns were the same. The color of the squares between the control, HUA, and AUA groups clearly changed, indicating that the content of differential metabolites between sample groups changed significantly. In addition, the contents of most metabolites in the AGA vs. control and AGA vs. HUA groups increased, consistent with the results shown in the volcano plot.

Through Venn diagram analysis, two core metabolites were found to be regulated in the HUA vs. control, AGA vs. control, and AGA vs. HUA groups (Figure 4D). A total of 24 metabolites were unique to HUA, 51 metabolites to AGA, and 89 metabolites to HUA vs. AGA. Table 2 shows the fold changes in the core metabolites of the three compared pairs. The content of cotinine, the main metabolic marker of smoking, gradually increased in the control, HUA, and AGA groups (Figure S5). This finding aligns with the significantly higher smoking prevalence observed in the AGA group (Table 2). Furthermore, L-homocitrulline, a metabolite implicated in protein carbamylation and linked to arginine and urea cycle metabolism, was significantly depleted in AGA patients compared to both control and HUA groups (Figure S5B,C).

### 3.6. KEGG Analysis of Differential Metabolites

**Figure 5:**
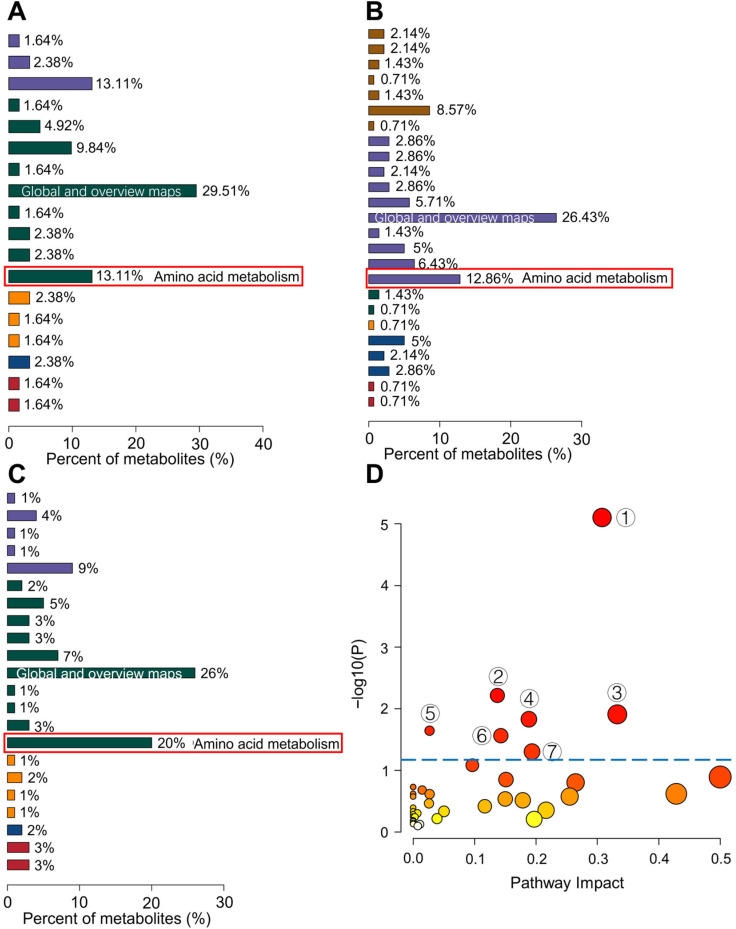
KEGG classification analysis of differential metabolites in the (**A**) HUA vs. control, (**B**) AGA vs. control, and (**C**) AGA vs. HUA groups. (**D**) Metabolic pathway analysis of all differential metabolites. The bubble color denotes the enrichment p-value, with darker shades representing greater significance. The bubble size represents the pathway impact factor from the topology analysis, with larger sizes indicating higher impact. ① Caffeine metabolism, ② arginine and proline metabolism, ③ nicotinate and nicotinamide metabolism, ④ histidine metabolism, ⑤ glycine, serine and threonine metabolism, ⑥ phenylalanine metabolism, and ⑦ tyrosine metabolism.

The Kyoto Encyclopedia of Genes and Genomes (KEGG) is a powerful tool for metabolic analysis and network research in organisms. A KEGG classification diagram was constructed according to the differential metabolites among the control, HUA, and AGA groups (Figure 5A–C). Among the three compared pairs, the number of differential metabolites annotated by the amino acid metabolic pathway was the greatest. We further analyzed the pathways of 277 kinds of differential metabolites by combining enrichment analysis and topological analysis. With the help of a hypergeometric test, the *p* value of pathway enrichment was obtained. The pathways with *p* < 0.05 were significantly enriched (Figure 5D and Table S6). The pathway analysis results revealed that seven metabolic pathways were significantly disturbed during gout: caffeine metabolism, arginine and proline metabolism, nicotinate and nicotinamide metabolism, histidine metabolism, glycine, serine, and threonine metabolism, phenylalanine metabolism, and tyrosine metabolism. The caffeine metabolism pathway is the most related to gout progression. Caffeine metabolism related to HUA and AGA progression were also found in blood and urine by Liu et al. [22] and Ohashi et al. [21]. Five pathways related to amino acid metabolism were significantly disturbed during gout development. Arginine and proline metabolism and glycine, serine, and threonine metabolism have been revealed by Shen et al. in serum, which are closely related to gout [31]. Histidine metabolism, nicotinate, and nicotinamide metabolism, phenylalanine metabolism, and tyrosine metabolism are the newly discovered metabolic pathways that are significantly disrupted in the development of gout. In addition, the purine metabolism pathway that induces HUA was enriched by the differential metabolites in this study, indicating that the process of HUA development into AGA is accompanied by changes in the pathway formed by HUA.

Because caffeine metabolism, arginine and proline metabolism, and glycine, serine, and threonine metabolism have been previously reported, we focused on the four newly discovered pathways. Figures S6–S9 show the changes in differential metabolites involved in histidine metabolism, nicotinate and nicotinamide metabolism, phenylalanine metabolism, and tyrosine metabolism. Table S4 shows that the histidine metabolism pathway was enriched with L-glutamate, histidine, and 3-methylhistidine. Nicotinamide, nicotinate ribonucleoside, and 1-methylnicotinamide were enriched in the nicotinate and nicotinamide metabolism pathway. The phenylalanine metabolism pathway was enriched with phenylacetaldehyde and tyrosine. Tyrosine metabolism was enriched with adrenaline, 3-methoxytyramine, tyrosine, and tyramine. These two metabolic pathways are interrelated through tyrosine.

### 3.7. Early Warning Biomarkers of Gout

To verify the reliability of the differential metabolites obtained by multivariate and univariate statistical analysis as biomarkers, we used receiver operating characteristic (ROC) curves to evaluate the accuracy of potential biomarkers in the diagnosis of AGA vs. HUA and AGA vs. control groups. ROC curves can reflect the relationship between the diagnostic sensitivity and specificity of potential biomarkers. The abscissa represents 1-specificity, and the ordinate represents sensitivity. The larger the area under the ROC curve (AUC) is, the greater the diagnostic accuracy of biomarkers will be. When the AUC was ≤0.5, the biomarker had no predictive value. The closer the AUC value was to one, the higher the accuracy of prediction was. When the AUC value was 0.5–0.7, the prediction accuracy was low. When the AUC value was 0.7–0.9, it had a certain prediction accuracy. When the AUC value was above 0.9, the prediction accuracy was high.

**Figure 6:**
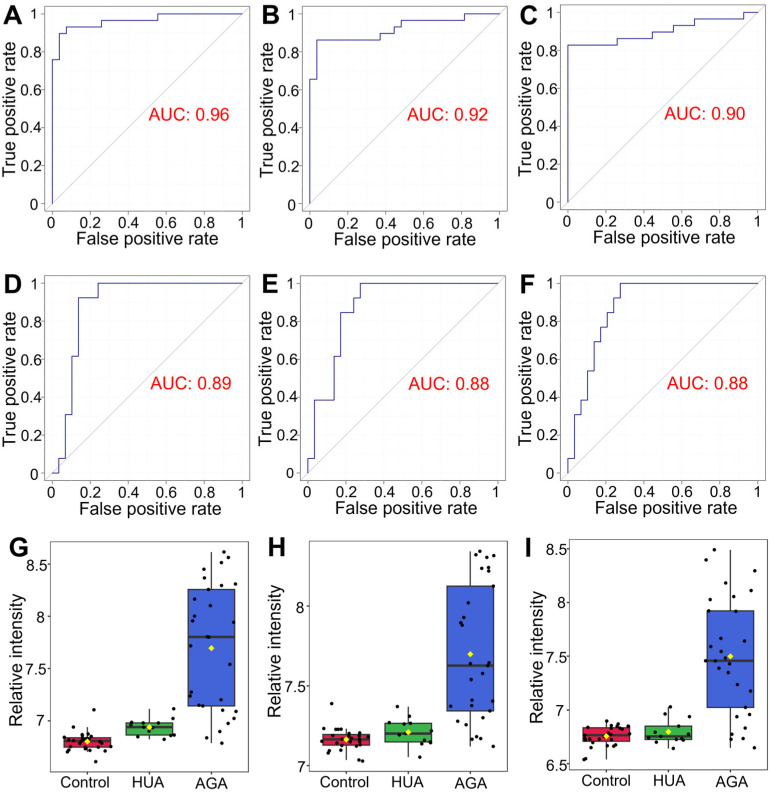
ROC curve analysis of the diagnosis of AGA and the control with (**A**) oxoamide, (**B**) 3-methylindole, and (**C**) palmitic acid. ROC curve analysis of (**D**) oxoamide, (**E**) 3-methylindole, and (**F**) palmitic acid in the diagnosis of AGA and HUA. Box diagram analysis of (**G**) oxoamide, (**H**) 3-methylindole, and (**I**) palmitic acid in the control, HUA, and AGA groups. HUA, hyperuricemia; AGA, acute gouty arthritis.

The differential metabolites in the AGA vs. control and the AGA vs. HUA groups were analyzed by ROC curves, and the AUC values of oxoamide, 3-methylindole, and palmitic acid were greater than 0.85 (Figure 6A–F). The changes of three differential metabolites in the control, HUA, and AGA groups were consistent (Figure 6G–I). The AUCs of oxoamide in the AGA vs. control and AGA vs. HUA groups were 0.96 and 0.89, respectively. The AUCs of 3-methylindole in the AGA vs. control and AGA vs. HUA groups were 0.92 and 0.88, respectively. The AUCs of palmitic acid in the AGA vs. control and AGA vs. HUA groups were 0.9 and 0.88, respectively. Therefore, these three differential metabolites have a certain predictive accuracy and can be used as biomarkers for the early diagnosis of AGA patients in the control and HUA groups. Previous studies have used similar methods to identify biomarkers that distinguish AGA and HUA in the serum metabolome and lipidome [31,32,33]. The markers of the urine metabolome were studied to distinguish healthy people, AGA patients, and chronic gouty arthritis patients [20]. Interestingly, the diagnostic markers of AGA found in this study have not been reported in previous studies.

## 4. Discussion

In this study, the urinary metabolomes of healthy people, asymptomatic HUA patients, and AGA patients were systematically analyzed by UHPLC-MS/MS technology. Based on PLS-DA analysis and the criteria of variable importance projection (VIP > 1), fold change (FC > 1.5 or <0.67), and statistical significance (*p* < 0.05), we identified 278 differential metabolites associated with gout progression. Notably, the number of specific differential metabolites was markedly greater between the AGA and control groups than between other comparison groups. This indicates that substantial metabolic reprogramming accompanies the transition from control to AGA. In-depth analysis revealed significant accumulation of cotinine, a biomarker of cigarette exposure, in the AGA group compared to controls (FC = 10.24, *p* = 1.80 × 10^−9^), which was highly consistent with the clinical characteristics of a higher smoking rate in the AGA group (AGA 67.9% vs. control 11.1%) (Table 1). These results suggest smoking may promote gout progression through perturbation of nicotine metabolism [34,35]. Furthermore, L-homocitrulline was significantly downregulated in the AGA group (AGA vs. control: FC = 0.61, *p* = 3.84 × 10^−4^, AGA vs. HUA: FC = 0.38, *p* = 2.79 × 10^−6^). This indicates that dysregulation of the urea cycle and arginine metabolism may represent a core pathological mechanism in acute gout attacks [36]. Collectively, these metabolomic findings reveal a pathogenic framework wherein exogenous environmental risk factors and endogenous metabolic disturbances jointly drive gout progression.

We identified seven metabolic pathways significantly dysregulated during gout progression. Among these, caffeine metabolism, arginine and proline metabolism, and glycine, serine, and threonine metabolism have been previously implicated in gout [21,22,31]. Importantly, urine metabolomic analysis revealed four novel dysregulated pathways for the first time: histidine metabolism, nicotinate and nicotinamide metabolism, phenylalanine metabolism, and tyrosine metabolism. This profile diverges from prior serum metabolomic studies, which primarily emphasized purine metabolism and broad amino acid pathways [31,37]. Histamine, a key substance in the histidine metabolism pathway, as a potent inflammatory mediator, may exacerbate local joint inflammation through mast cell activation [38]. Thangam et al. [39] indicated that hypertrophied mast cells serve as the primary source of histamine, providing mechanistic support for our findings. The disorder of the stress hormone adrenaline and neurotransmitter tyramine produced by phenylalanine metabolism and tyrosine metabolism indicates that the neural–immune regulatory network is deeply involved in acute attacks of gout [40,41]. Reiske et al. [42] demonstrated that adrenaline, a core catecholamine neurotransmitter of the sympatho-adrenal medullary axis, directly modulates immune cell activity. Separately, Kuo et al. [43] observed tyramine-induced transient immune responses in crustaceans. Collectively, this evidence suggests dysregulation of these metabolic pathways may drive gout inflammatory responses through neurotransmitter-mediated immunomodulation. Furthermore, nicotinamide serves as the key precursor for the coenzymes nicotinamide adenine dinucleotide (NAD^+^) and nicotinamide adenine dinucleotide phosphate (NADP^+^). Consequently, the disturbance of nicotinate and nicotinamid metabolism is closely related to the biosynthesis of NAD^+^ and NADP^+^ and the steady state of energy metabolism [44]. Yang et al. [45] demonstrated that inflammatory factors drive T-cell metabolic reprogramming to promote oral lichen planus progression. Building on this evidence, dysregulation of the nicotinate and nicotinamide metabolism pathway may modulate inflammasome activity, thereby influencing inflammatory responses in gout. This implies that energy metabolism and oxidative stress play a key role in the pathogenesis of gout, which provides a new mechanism for research.

While previous investigations by Wang et al. [46] and Lyu et al. [37] have identified gout metabolic markers in blood, this study is the first to report three non-invasive urinary metabolites demonstrating high diagnostic efficacy (AUC > 0.85) for gout progression: oxoamide, 3-methylindole, and palmitic acid. Critically, these metabolites exhibit progressive alterations across the disease continuum from healthy individuals to HUA and AGA. The inherent suitability of urine for repeated and non-invasive sampling offers a unique advantage for dynamically monitoring disease activity using these markers. Furthermore, these metabolites exhibit a robust discriminatory capacity in distinguishing AGA from HUA. This positions them as promising tools for identifying HUA patients at high risk of progression to AGA, enabling early intervention to potentially halt disease development.

To sum up, this study describes the dynamic metabolic profiles of gout progression using untargeted urine metabolomics, identifying four novel dysregulated pathways and three high-efficacy predictive biomarkers. These findings provide a mechanistic foundation for advancing understanding of gout pathogenesis and developing non-invasive diagnostic strategies. Limitations include the cohort’s modest sample size, relatively single sampling source, unvalidated biological functions of the identified pathways in experimental models, and the absence of external biomarker validation in independent cohorts. Future research should establish multi-center prospective cohorts, incorporate gender-balanced design, elucidate pathway mechanisms, and integrate multi-omics validation to translate these discoveries into clinical early-warning tools, ultimately improving long-term outcomes and quality of life in gout patients.

## 5. Conclusions

In this study, we employed nontargeted urine metabolomics to systematically characterize dynamic metabolic alterations during the progression from healthy people to AGA via HUA. A total of 278 differential metabolites were identified in urine profiles across the three cohorts. Notably, the number of specific differential metabolites distinguishing AGA patients from healthy people significantly exceeded those differentiating other groups, indicating profound metabolic pathway remodeling during AGA development. KEGG pathway analysis revealed four gout disturbance pathways for the first time in urine: histidine metabolism, nicotinate and nicotinamide metabolism, phenylalanine metabolism, and tyrosine metabolism. These findings provide new evidence suggesting the involvement of neural–endocrine–immune network imbalance and energy metabolism dysregulation in gout. Furthermore, we identified three highly specific urinary biomarkers for AGA via ROC analysis: oxoamide, 3-methylindole, and palmitic acid. Their gradient changes across the disease continuum position them as promising non-invasive tools for early gout detection. In summary, this study delineates urinary metabolic alterations associated with gout progression, where the identification of novel pathways and a highly specific biomarker panel not only elucidates gout pathogenesis but also facilitates early diagnosis and targeted intervention.

## Figures and Tables

**Table 1:** Clinical and demographic characteristics of the study participants *.

	Normal Controls (n = 28)	Patients with HUA (n = 13)	Patients with AGA (n = 29)
Age, years, median (IQR)	26 (24–28)	26 (24–27)	32 (22–51) ^†,‡^
BMI, kg/m^2^, median (IQR)	23.4 (19.6–28.4)	26.3 (22.0–29.3) ^†^	26.5 (17.0–32.0) ^†^
Smoking, n (%) ^a^	2 (11.1)	1 (10.0)	19 (67.9) ^†,‡^
Drinking, n (%) ^b^	0 (0)	1 (10.0)	7 (25.0) ^†^
Beverage, n (%) ^c^	8 (44.4)	3 (30.0)	9 (32.1)
Sleep time, hours/day, median (IQR)	7 (6–8)	7 (4–8)	7 (5.5–8) ^†^
ALT, units/liter, median (IQR)	25.1 (7.7–70.8)	29.2 (19.8–65.2)	35.9 (16.9–91.6) ^†^
AST, units/liter, median (IQR)	20.8 (8.9–67.6)	24.2 (12–30.6)	25.1 (10.4–54)
GLU, mmoles/liter, median (IQR)	4.7 (3.5–5.6)	4.9 (4.3–5.1)	5.0 (3.8–6.8) ^†^
TG, mmoles/liter, median (IQR)	0.9 (0.5–2.2)	1.3 (0.7–1.9)	1.4 (0.7–4.2) ^†^
TCH, mmoles/liter, median (IQR)	4.7 (3.4–6.1)	5.0 (3.8–6.3)	4.6 (1.8–6.9)
BUN, mmoles/liter, median (IQR)	5.5 (3.7–8.4)	4.8 (3.7–5.8) ^†^	5.1 (3.9–7.7)
CR, mmoles/liter, median (IQR)	78.0 (62.0–93.0)	86.0 (67.0–96.0)	81.1 (65.0–106.0)
SUA, µmoles/liter, median (IQR)	342.0 (231.6–427.3)	477.1 (431.8–567.9) ^†^	465.0 (336.0–696.0) ^†^

* Complete data was not available for every participant. For continuous variables, values are presented as median (quartile). For categorical variables, data are presented as n (%). BMI = body mass index; ALT = alanine aminotransferase; AST = aspartate aminotransferase; GLU = glucose; TG = triglycerides; TCH = total cholesterol; BUN = blood urea nitrogen; CR = creatinine; SUA = serum urate acid. ^a^ At least 20 cigarette packs in a lifetime or at least one cigarette a day for at least 1 year. ^b^ Alcohol intake at least once a week for 6 months. ^c^ Beverage intake at least twice a week for 6 months. ^†^ *p* < 0.05 versus normal controls. ^‡^ *p* < 0.05 versus participants with HUA.

**Table 2:** Core differential metabolites in the HUA vs. control, AGA vs. control, and AGA vs. HUA groups.

	Cotinine				L-Homocitrulline			
	FC	log2FC	*p* Value	VIP	FC	log2FC	*p* Value	VIP
HUA vs. control	1.71	0.77	5.24 × 10^−7^	1.10	1.61	0.69	6.87 × 10^−3^	1.92
AGA vs. control	10.24	3.36	1.80 × 10^−9^	2.99	0.61	−0.72	3.84 × 10^−4^	1.67
AGA vs. HUA	5.98	2.58	1.88 × 10^−6^	2.01	0.38	−1.41	2.79 × 10^−6^	3.18

## Data Availability

Data supporting this study are available from the corresponding author upon reasonable request.

## Supplementary Materials

The following supporting information can be downloaded at https://www.mdpi.com/article/10.3390/metabo15090580/s1: Figure S1: Pie chart of metabolite classification annotated by UHPLC-MS/MS positive ion mode (A) and negative ion mode (B) in the urine of control, HUA, and AGA patients. Figure S2: The metabolites detected by UHPLC-MS/MS in the urine of control, HUA, and AGA patients were used to annotate the KEGG pathway. Figure S3: The metabolites detected by UHPLC-MS/MS in the urine of control, HUA, and AGA patients were annotated by HMDB. Figure S4: The metabolites detected by UHPLC-MS/MS in the urine of control, HUA, and AGA patients were annotated with LIPID MAPS. Figure S5: Box diagram analysis of core regulatory metabolites cotinine (A) and L-homoclinic (b) in control, HUA, and AGA patients. Figure S6: Changes of differential metabolites in the histidine metabolism pathway. Figure S7: Changes of differential metabolites in the nicotinate and nicotinamid metabolism pathway. Figure S8: Changes of differential metabolites in the phenylalanine metabolism pathway. Figure S9: Changes of differential metabolites in the tyrosine metabolism pathway. Table S1: Metabolites identified in positive ion mode. Table S2: Metabolites identified in negative ion mode. Table S3: Differential metabolites in HUA vs. control. Table S4: Differential metabolites in AGA vs. control. Table S5: Differential metabolites in AGA vs. HUA. Table S6: Pathways significantly enriched in differential metabolite pathway analysis among the HUA vs. control, AGA vs. control, and AGA vs. HUA groups.

## Abbreviations

The following abbreviations are used in this manuscript:

HUA	Hyperuricemia
AGA	Acute gouty arthritis
ROC	Receiver operating characteristic
UHPLC-MS	Ultrahigh-performance liquid chromatography–mass spectrometry
PLS-DA	Partial least squares discriminant analysis
FC	Fold change
PCA	Principal component analysis
VIP	Variable importance in projection
BMI	Body mass index
ALT	Alanine aminotransferase
AST	Aspartate aminotransferase
GLU	Glucose
TG	Triglyceride
TCH	Total cholesterol
BUN	Blood urea nitrogen
CR	Creatinine
SUA	Serum urate acid
QC	Quality control
KEGG	Kyoto Encyclopedia of Genes and Genomes
AUC	Area under curve
NAD^+^	Nicotinamide adenine dinucleotide
NADP^+^	Nicotinamide adenine dinucleotide phosphate
