# Supplementary material for: Urine Metabolomics of Gout Reveals the Dynamic Reprogramming and Non-Invasive Biomarkers of Disease Progression
Source: Metabolites. 2025 Aug 29;15(9):580. doi: 10.3390/metabo15090580 (PMC12471545; doi:10.3390/metabo15090580)
Supplement: Supplementary file 1 [file metabolites-15-00580-s001.zip › metabolites-3821941-supplementary.pdf]

Supplementary Information

Urine Metabolomics of Gout Reveals the Dynamic Reprogramming and Non-Invasive Biomarkers of Disease Progression

Guizhen Zhu [1,†], Yuan Luo [1,†], Nan Su [2,†], Xiangyi Zheng [1], Zhusong Mei [1], Qiao Ye [1], Jie Peng [1], Peiyu An [1], Yangqian Song [3], Weina Luo [4], Hongxia Li [3,*], Guangyun Wang [1,*] and Haitao Zhang [4,*]

[1] Laboratory of Clinical Medicine, Air Force Medical Center, Air Force Medical University, People's Liberation Army of China, Beijing 100142, China; 13121437811@163.com (G.Z.); ly-navy@163.com (Y.L.); zhengxy12345@163.com (X.Z.); mzsa@163.com (Z.M.); yeqiao333@163.com (Q.Y.); 19991205301@163.com (J.P.); yupeipyjj@sina.com (P.A.)

[2] Military Medical Center, Air Force Medical Center, Air Force Medical University, People's Liberation Army of China, Beijing 100142, China; 13911586246@139.com

[3] Rheumatology and Immunology Department, Air Force Medical Center, Air Force Medical University, PLA, Beijing 100142, China; m15168405793@163.com

[4] Cardiovascular Department, Air Force Medical Center, Air Force Medical University, People's Liberation Army of China, Beijing 100142, China; luoweina_215@163.com

* Correspondence: hxli2005@126.com (H.L.); gfkdwgy@163.com (G.W.); kjzht@sina.com (H.Z.)

† These authors contributed equally to this work.

Supporting Figures

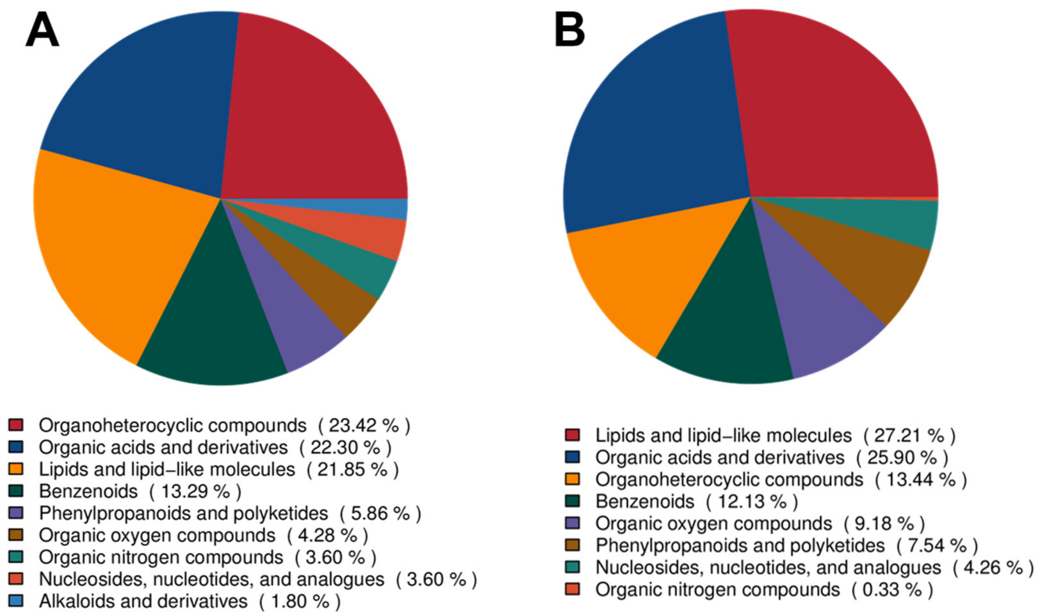

Figure S1 Pie chart of metabolite classification annotated by UHPLC-MS/MS positive ion mode (A) and negative ion mode (B) in urine of control, HUA, and AGA patients.

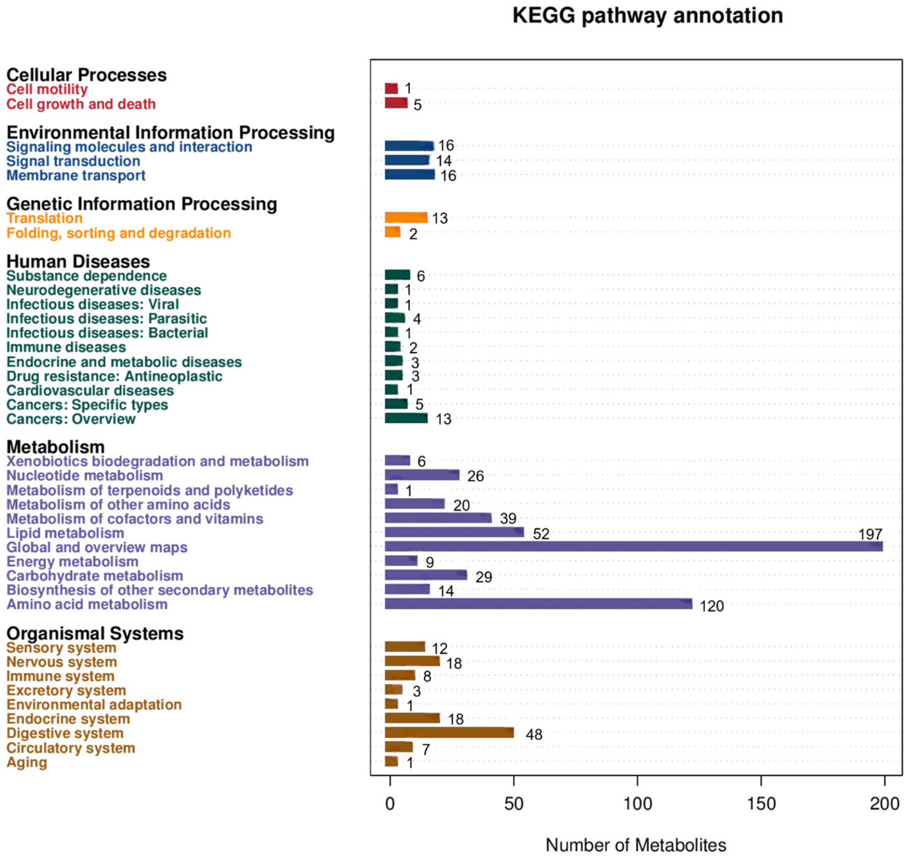

Figure S2 The metabolites detected by UHPLC-MS/MS in urine of control, HUA,

and AGA patients were used to annotate KEGG pathway.

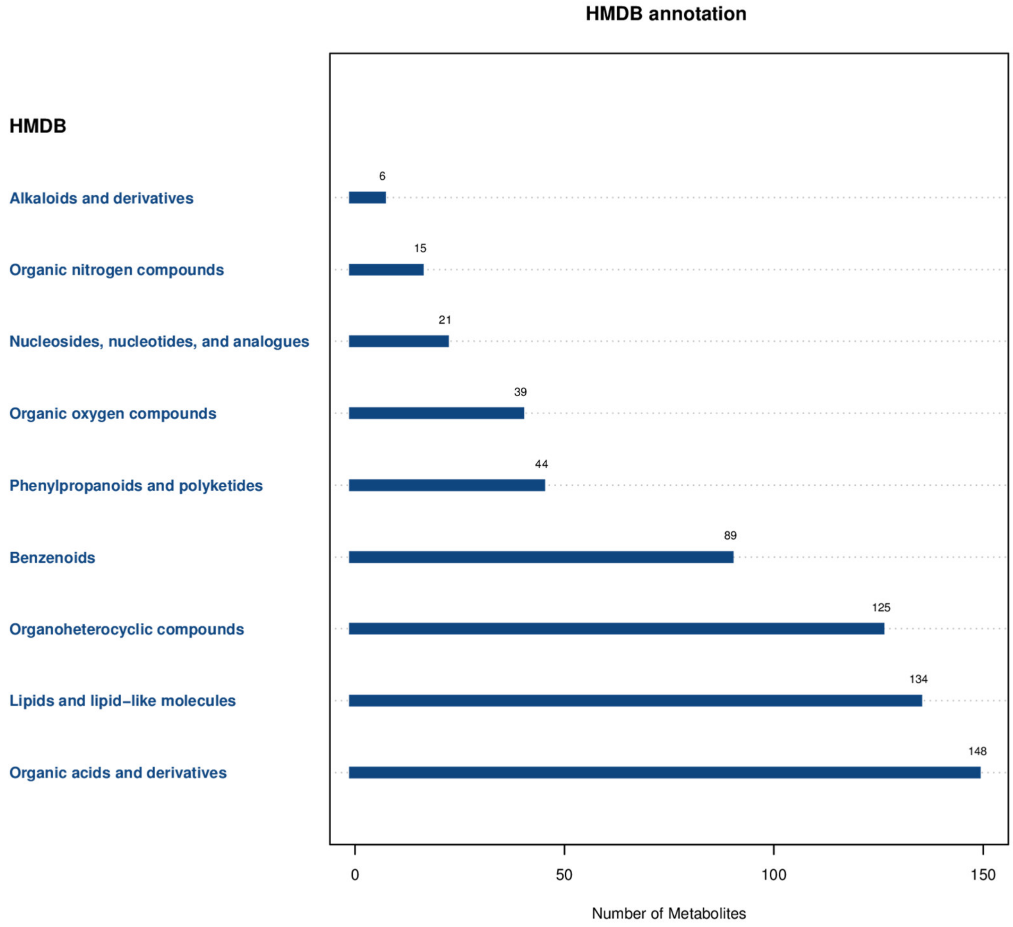

Figure S3 The metabolites detected by UHPLC-MS/MS in the urine of control, HUA, and AGA patients were annotated by HMDB.

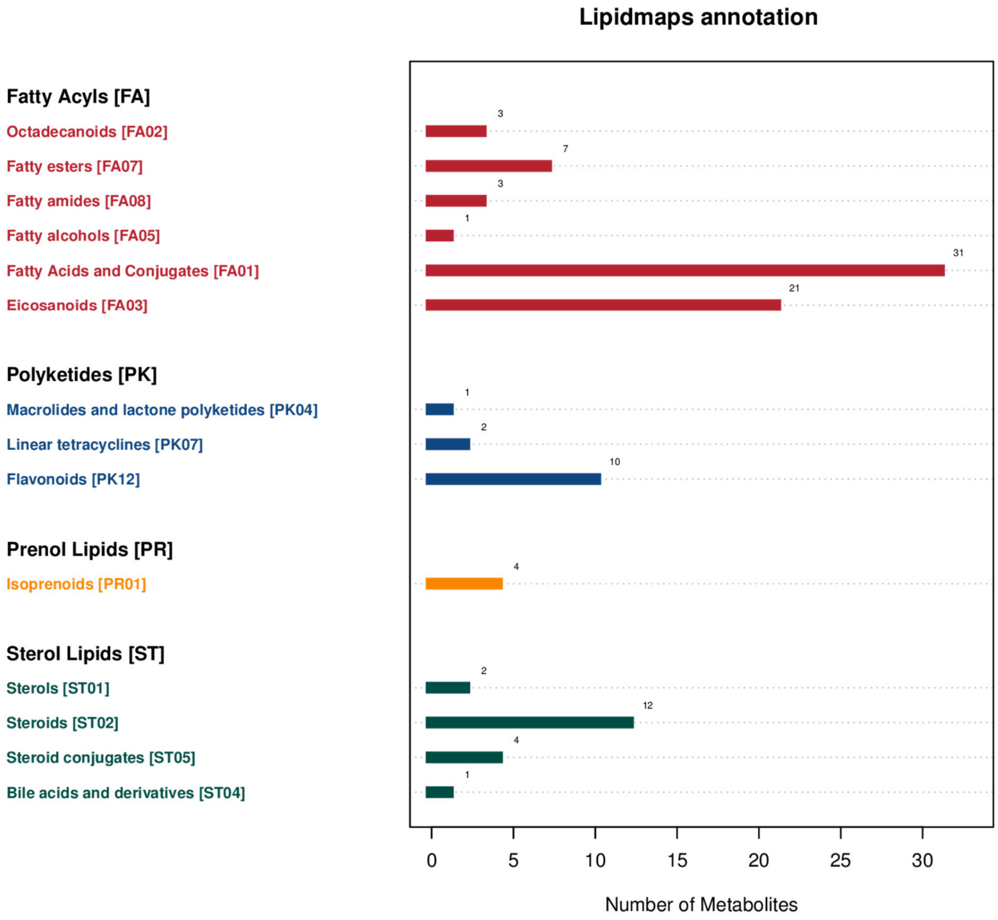

Figure S4 The metabolites detected by UHPLC-MS/MS in the urine of control, HUA, and AGA patients were annotated with lipidmaps.

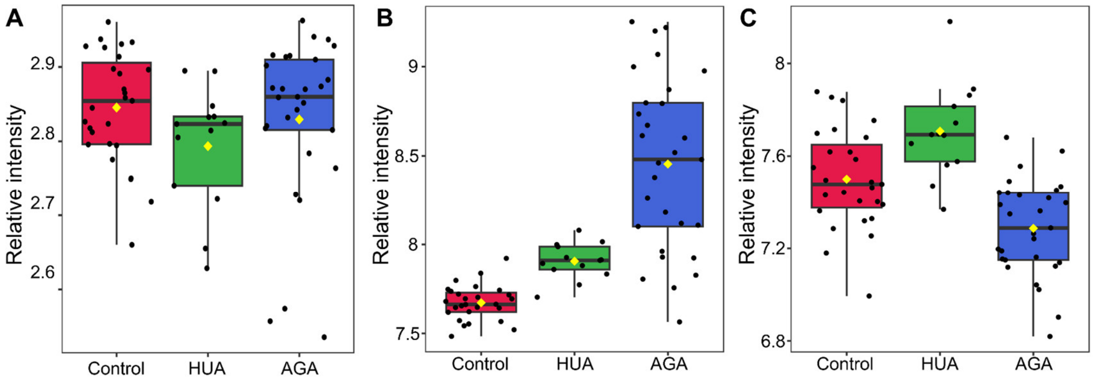

Figure S5 Box diagram analysis of uric acid (A), cotinine (B), and L-homoclinic (C) in control, HUA, and AGA patients.

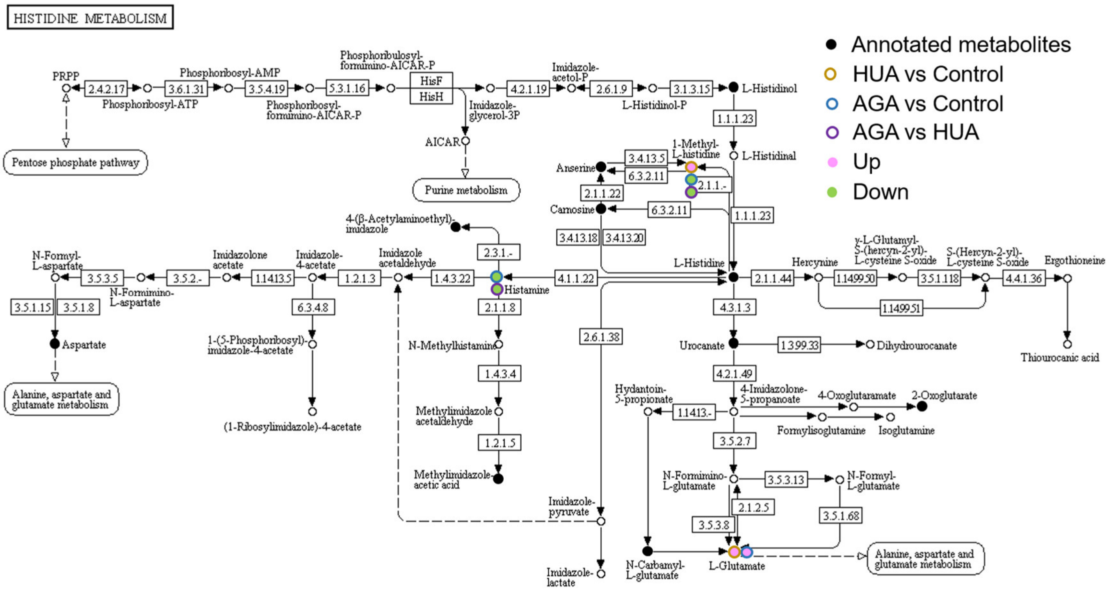

Figure S6 Changes of differential metabolites in histidine metabolism pathway.

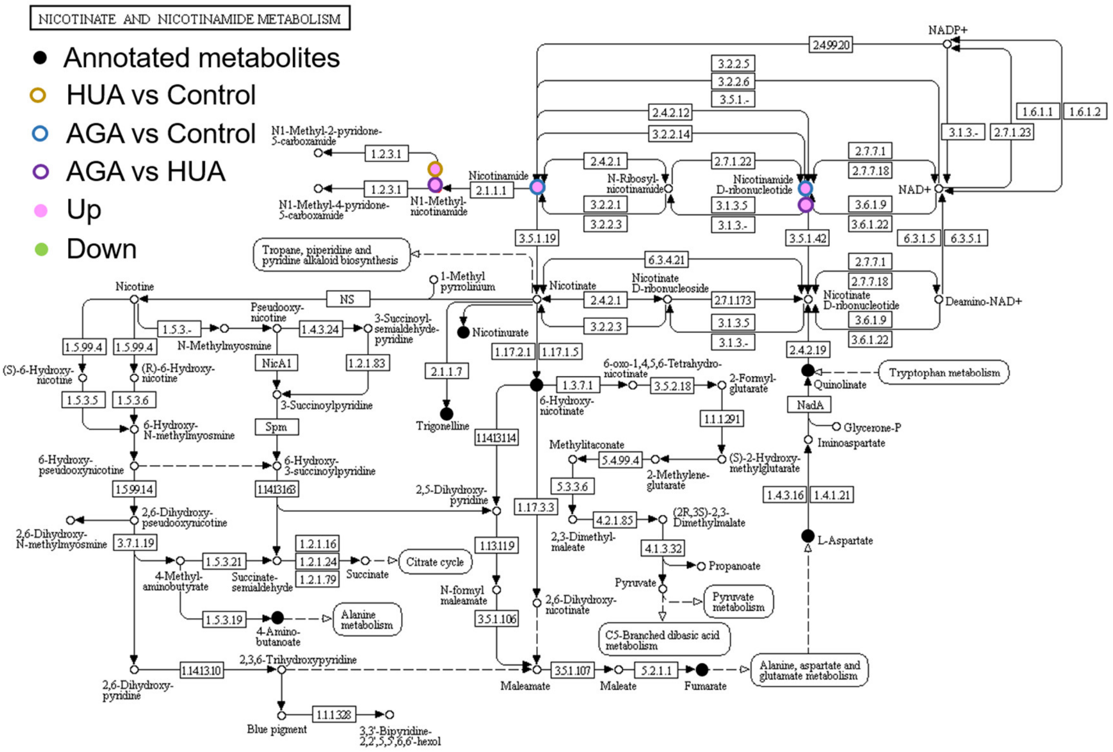

Figure S7 Changes of differential metabolites in nicotinate and nicotinamid metabolism pathway.

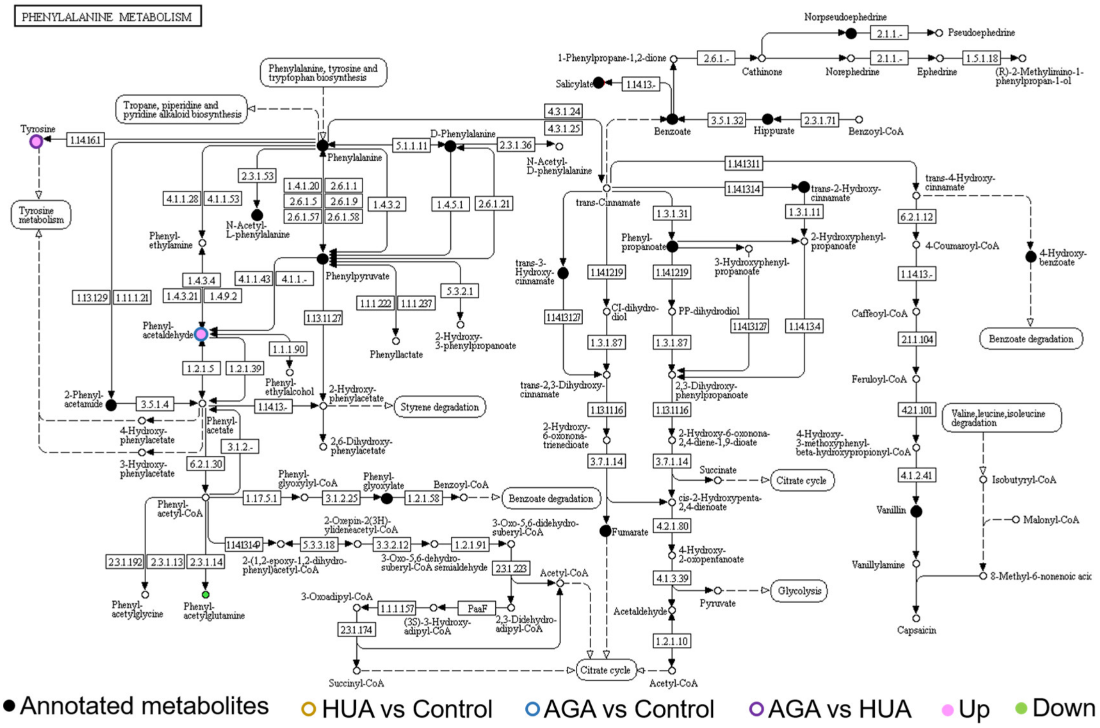

Figure S8 Changes of differential metabolites in phenylalanine metabolism pathway.

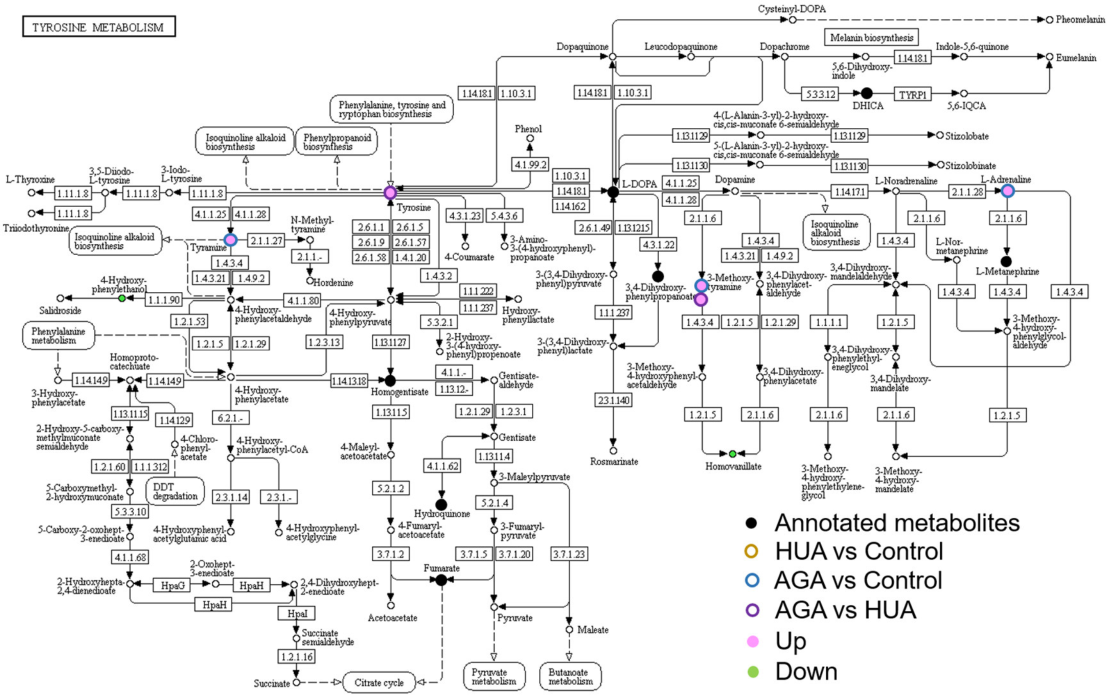

Figure S9 Changes of differential metabolites in tyrosine metabolism pathway.

Supporting Tables

Table S1 Metabolites identified in positive ion mode.

No.	Metabolites	Formula	Molecular Weight	m/z
1	Caprolactam	C6H11NO	113.0843	114.0916
2	CAR 10:1	C17H32NO4	313.2252	314.2325
3	Creatine	C4H9N3O2	131.0697	132.0769
4	DL-Carnitine	C7H15NO3	161.1051	162.1125
5	CAR 7:0	C14H28NO4	273.1941	274.2014
6	CAR 9:1	C16H30NO4	299.2097	300.217
7	DL-Tryptophan	C11H12N2O2	204.09	188.0707
8	Decanoylcarnitine	C17H33NO4	315.2411	316.2483
9	Indole-3-acrylic acid	C11H9NO2	187.0635	188.0707
10	Paraxanthine	C7H8N4O2	180.0649	181.0721
11	trans-3-Hydroxycotinine	C10H12N2O2	192.0899	193.0972
12	Trigonelline	C7H7NO2	137.0476	138.0549
13	Acetyl-L-carnitine	C9H17NO4	203.1157	204.123
14	Cathine	C9H13NO	151.0999	134.0966
15	Indole	C8H7N	117.0582	118.0654
16	CAR 10:2	C17H30NO4	311.2097	312.217
17	4-Guanidinobutyric acid	C5H11N3O2	145.0851	146.0924
18	N-Benzylformamide	C8H9NO	135.0687	118.0655
19	4-decyl-3-hydroxy-5-oxooxolane-2,3-dicarboxylic acid	C16H26O7	352.15	353.1573
20	L-Adrenaline	C9H13NO3	183.0897	184.097
21	α-Hydroxyhippuric acid	C9H9NO4	195.0534	196.0607
22	N-Acetylvaline	C7H13NO3	159.0895	160.0968
23	1-Methylnicotinamide	C7H8N2O	136.0637	137.071
24	N6,N6,N6-Trimethyl-L-lysine	C9H20N2O2	188.1526	189.1598
25	6-Methylquinoline	C10H9N	143.0736	144.0809
26	CAR 12:3	C19H32NO4	337.2253	338.2326
27	Norfloxacin	C16H18FN3O3	319.1334	320.1407
28	Caffeine	C8H10N4O2	194.0806	195.0878
29	N3,N4-Dimethyl-L-arginine	C8H18N4O2	202.143	203.1503
30	7-Methylguanine	C6H7N5O	165.0651	166.0724
31	Febuxostat	C16H16N2O3S	316.0882	317.0955
32	Betaine	C5H11NO2	117.0792	118.0865
33	Oleamide	C18H35NO	281.2718	282.2791
34	pentane-1,2,3,4,5-pentol	C5H12O5	174.0505	175.0578
35	5-ethoxy-2-[(2,3,4,5,6-pentafluorobenzyl)thio]-1H-benzo[d]imidazole	C16H11F5N2OS	374.0493	375.0566

36	Methylimidazoleacetic acid	C6H8N2O2	140.0586	141.0659
37	Theobromine	C7H8N4O2	180.0649	181.0722
38	Tramadol N-Oxide	C16H25NO3	279.1836	280.1908
39	1-Methyladenine	C6H7N5	149.0708	150.0776
40	H-Pro-Hyp-OH	C10H16N2O4	228.1111	229.1184
41	Hexanoylcarnitine	C13H25NO4	259.1787	260.186
42	2,3-Dinor-11β-prostaglandin F2α	C18H30O5	343.2361	344.2433
43	DL-Stachydrine	C7H13NO2	160.1213	161.1286
44	RPK	C17H33N7O4	399.2622	400.2695
45	XLR11 N-(4-hydroxypentyl) metabolite	C21H28FNO2	345.2152	346.2224
46	Prolylleucine	C11H20N2O3	228.1475	229.1548
47	Nitrosoheptamethyleneimine	C7H14N2O	142.1109	126.0916
48	Quinoline	C9H7N	129.058	130.0653
49	6-Methylnicotinamide	C7H8N2O	136.0638	137.0711
50	(1R,2R)-trans-N-Boc-1,2-cyclohexanediamine	C11H22N2O2	214.1684	215.1756
51	Nicotine	C10H14N2	162.1157	163.123
52	UR-144 N-(5-Methylhexyl) analog	C23H33NO	339.2523	340.2596
53	gamma-Glutamylleucine	C11H20N2O5	260.1375	261.1448
54	6-Pentyl-2H-pyran-2-one	C10H14O2	166.0996	167.1069
55	Stiripentol	C14H18O3	216.1149	217.1225
56	fentanyl-d5	C22H23D5H5N2O	341.2568	342.264
57	Cotinine	C10H12N2O	176.095	177.1023
58	Pyridoxal	C8H9NO3	167.0584	168.0657
59	DL-Lysine	C6H14N2O2	146.1056	147.1129
60	2-methyl-4-[(3-methyl-2-thienyl)methylene]-1,3-oxazol-5(4H)-one	C10H9NO2S	207.0356	208.0429
61	2-Hydroxycinnamic acid	C9H8O3	164.0475	165.0547
62	Propionylcarnitine	C10H19NO4	217.1316	218.1388
63	Isoferulic acid	C10H10O4	194.0582	195.0655
64	ILK	C18H36N4O4	354.256	355.2633
65	DL-Norvaline	C5H11NO2	117.0795	118.0867
66	1-(4-benzylpiperazino)-2-(pyridin-2-ylamino)propan-1-one	C19H24N4O	362.1555	363.1627
67	15-Deoxy-Δ12,14-prostaglandin A1	C20H30O3	318.2196	319.2268
68	1-[2-(2,5-dimethyl-1H-pyrrol-1-yl)-4-nitrophenyl]-1H-imidazole	C15H14N4O2	282.1082	283.1155
69	Isovanillic acid	C8H8O4	168.0424	169.0497
70	2,4-Dimethylbenzaldehyde	C9H10O	134.0734	135.0806
71	(5E)-7-methylidene-10-oxo-4-(propan-2-yl)undec-5-enoic acid	C15H24O3	252.1728	253.1801
72	Prostaglandin F2α-1-glyceryl ester	C23H40O7	900.5033	451.2589
73	16(R)-HETE	C20H32O3	325.189	326.1964

74	3-(propan-2-yl)-octahydropyrrolo[1,2-a]pyrazine-1,4-dione	C10H16N2O2	196.1214	197.1286
75	3',5,7-Trihydroxy-4'-methoxyflavanone	C16H14O6	302.0792	303.0864
76	CAR 12:0	C19H38NO4	343.2725	344.2798
77	4-oxododecanedioic acid	C12H20O5	266.1132	267.1205
78	2-Methoxyresorcinol	C7H8O3	140.0474	141.0547
79	Hypoglycin A	C7H11NO2	141.0791	142.0863
80	Pyridoxamine	C8H12N2O2	168.09	169.0972
81	1H-indene-3-carboxamide	C10H9NO	159.0687	160.0759
82	o-Cresol	C7H8O	108.0579	109.0652
83	Palmitic Acid	C16H32O2	273.2668	274.2741
84	1-Methylhistidine	C7H11N3O2	169.085	170.0923
85	Tetrahydrocortisone	C21H32O5	346.2144	347.2217
86	SQH	C14H22N6O6	370.1606	371.1678
87	Vatalanib dihydrochloride	C20H15ClN4	346.0986	369.0879
88	7-Methylguanosine	C11H15N5O5	297.1074	298.1147
89	Guanidineacetic acid	C3H7N3O2	117.0541	118.0614
90	Methyl nicotinate	C7H7NO2	137.0477	138.055
91	Apocynin	C9H10O3	166.0631	167.0705
92	Cuminaldehyde	C10H12O	148.089	149.0963
93	Debromohymenialdisine	C11H11N5O2	512.1664	513.1737
94	1-(4-hydroxyphenyl)propane-1,2-diol	C9H12O3	150.0682	151.0755
95	N,N-dimethyl-9H-purin-6-amine	C7H9N5	163.0859	164.0932
96	Acetylcholine	C7H15NO2	145.1103	146.1176
97	4-(tert-butyl)phenyl 3,5-dimethylisoxazole-4-carboxylate	C16H19NO3	273.1366	274.1439
98	4-Hydroxy-6-methyl-2-pyrone	C6H6O3	126.032	127.0392
99	L(-)-Carnitine	C7H15NO3	161.1053	162.1125
100	N8-Acetylspermidine	C9H21N3O	187.1686	188.1759
101	Octopamine	C8H11NO2	153.0791	154.0864
102	N1-(2,3-dihydro-1,4-benzodioxin-6-yl)acetamide	C10H11NO3	193.0741	194.0814
103	Methylparaben	C8H8O3	152.0476	135.0443
104	Arachidonoyl amide	C20H33NO	303.2536	304.2609
105	Tacrolimus	C44H69NO12	1606.936	804.4751
106	1-O-(3,4,5-Trimethoxybenzoyl)-beta-L-galactopyranose	C16H22O10	396.1035	397.1108
107	2-Oxindole	C8H7NO	133.053	134.0602
108	Urocanic acid	C6H6N2O2	138.043	139.0503
109	Oxohongdenafil	C25H32N6O4	480.255	481.2623
110	(2E)-3-phenyl-N-(2-phenylethyl)prop-2-enamide	C17H17NO	273.1115	274.1188
111	1-Methyladenosine	C11H15N5O4	281.1123	282.1196
112	Guaiacol	C7H8O2	141.0541	124.0508

113	1-(4-bromophenyl)-2-phenylethan-1-one	C14H11BrO	223.1208	224.1279
114	5,8-dihydroxy-10-methyl-5,8,9,10-tetrahydro-2H-oxecin-2-one	C10H14O4	220.0713	221.0786
115	HPH	C17H23N7O4	371.1733	372.1807
116	3-Hydroxyfluorene	C13H10O	182.0733	165.07
117	(2S)-2-(2-hydroxypropan-2-yl)-2H,3H,7H-furo[3,2-g]chromen-7-one	C14H14O4	246.087	247.0943
118	2-[2-oxo-2-(pyridin-3-ylamino)ethoxy]acetic acid	C9H10N2O4	210.0643	211.0716
119	4-Methoxycinnamaldehyde	C10H10O2	162.0682	163.0755
120	3-Methoxycinnamic acid	C10H10O3	160.0526	161.0598
121	Choline	C5H13NO	103.1002	104.1074
122	N1-[5-(3,5-dimethylpiperidino)-4-fluoro-2-nitrophenyl]acetamide	C15H20FN3O3	309.1477	310.155
123	Citral	C10H16O	152.1202	153.1275
124	methyl 4-oxo-4H-benzo[4,5]imidazo[2,1-b][1,3]thiazine-2-carboxylate	C12H8N2O3S	260.0256	261.0329
125	N-(1-benzyl-4-piperidinyl)-4-(1H-pyrazol-1-yl)benzamide	C22H24N4O	398.1558	399.163
126	2-Hydroxyphenylalanine	C9H11NO3	181.074	182.0813
127	Vanillin	C8H8O3	152.0475	153.0548
128	β-Cortolone	C21H34O5	348.2304	349.2374
129	3-hydroxy-2-octylpentanedioic acid	C13H24O5	242.1521	243.1593
130	4-Methoxybenzaldehyde	C8H8O2	154.0632	155.0705
131	N-Acetylserotonin	C12H14N2O2	218.1057	219.113
132	5-fluoro AB-PINACA N-(4-hydroxypentyl) metabolite	C18H25FN4O3	364.1856	365.1929
133	5α-Dihydrotestosterone	C19H30O2	290.2246	273.2214
134	Tetracycline	C22H24N2O8	426.1513	427.158
135	7-methyl-3-nitroimidazo[1,2-a]pyridine	C8H7N3O2	199.0335	200.0408
136	Emetine	C29H40N2O4	960.5981	481.3063
137	Cer 9:1;2O/18:4	C27H45NO3	431.3403	432.3476
138	D-Proline	C5H9NO2	115.0636	116.0709
139	Acetophenone	C8H8O	120.0578	121.0651
140	Cytosine	C4H5N3O	111.0437	112.051
141	6-(3-hydroxybutan-2-yl)-5-(hydroxymethyl)-4-methoxy-2H-pyran-2-one	C11H16O5	266.0514	267.0586
142	trans-Cinnamaldehyde	C9H8O	132.0578	133.065
143	3-(4-benzylpiperazino)pyrazine-2-carbonitrile	C16H17N5	279.1473	280.1545
144	11-Epiprostaglandin E1	C20H34O5	371.267	372.2743
145	2-Phenylacetamide	C8H9NO	135.0686	136.0758
146	3-phenethyl-2-thioxoimidazolidin-4-one	C11H12N2OS	220.0673	221.0746
147	2-Hydroxybenzothiazole	C7H5NOS	151.0095	152.0168

148	Methyl indole-3-acetate	C11H11NO2	189.0791	212.0684
149	T-2 Triol	C20H30O7	382.1964	383.2041
150	L-Tyrosine	C9H11NO3	181.0739	182.0811
151	7-(2-hydroxypropan-2-yl)-1,4a-dimethyl-decahydronaphthalen-1-ol	C15H28O2	257.2357	258.243
152	4-(4-methoxyphenyl)-6-pyridin-4-yl-1,3,5-triazin-2(3H)-one	C15H12N4O2	280.0925	281.0998
153	L-(+)-Citrulline	C6H13N3O3	175.0958	176.103
154	1-Naphthol	C10H8O	144.0576	145.0649
155	GNK	C12H23N5O5	317.17	318.1773
156	4-methyl-5-phenoxy-6-piperidino-2-(trifluoromethyl)pyrimidine	C17H18F3N3O	337.1382	338.1455
157	2-(2-oxo-2-{[2-(2-oxo-1-imidazolidinyl)ethyl]amino}ethoxy)acetic acid	C9H15N3O5	245.103	246.1103
158	Aflatoxin G1	C17H12O7	328.0584	329.0656
159	trans-3-Indoleacrylic acid	C11H9NO2	187.0635	188.0708
160	(R)-3-Hydroxy myristic acid	C14H28O3	226.1932	227.2004
161	Methylhippuric acid	C10H11NO3	193.0742	194.0815
162	(2E)-3-(3,4-dimethoxyphenyl)prop-2-enoic acid	C11H12O4	190.0632	191.0705
163	(+/-)-Equol	C15H14O3	242.0947	243.102
164	Desoxycortone	C21H30O3	330.2195	331.2268
165	Cotinine N-oxide	C10H12N2O2	192.0901	193.0974
166	5-amino-2-(dimethylamino)benzoic acid	C9H12N2O2	180.09	181.0973
167	4-(2,3-dihydro-1,4-benzodioxin-6-yl)butanoic acid	C12H14O4	222.0894	223.0967
168	4,4'-dimethoxy[1,1'-biphenyl]-2-carbonitrile	C15H13NO2	239.0947	240.102
169	Mesalamine	C7H7NO3	153.0427	154.0499
170	5'-S-Methyl-5'-thioadenosine	C11H15N5O3S	297.0897	298.097
171	TNK	C14H27N5O6	361.1965	362.2034
172	3,5-Dihydroxyphenylglycine	C8H9NO4	183.0533	184.0606
173	4-Phenyl-3-buten-2-one	C10H10O	146.0733	147.0806
174	D-(+)-Camphor	C10H16O	152.1203	153.1275
175	N-α-L-Acetyl-arginine	C8H16N4O3	216.1218	217.1291
176	2-(benzylthio)-4-[2-(3-methylbenzo[b]thiophen-2-yl)vinyl]pyrimidine	C22H18N2S2	374.0939	375.1011
177	Arecoline	C8H13NO2	155.0948	156.1021
178	Riboflavin	C17H20N4O6	376.1378	377.1451
179	Leucylproline	C11H20N2O3	228.1476	229.1548
180	D-(+)-Maltose	C12H22O11	364.0981	365.1054
181	N,N'-Diphenylurea	C13H12N2O	212.0952	213.1025
182	N-Acetylhistamine	C7H11N3O	153.0904	154.0977
183	N-(2-Furoyl)glycine	C7H7NO4	169.0376	170.0449
184	Obscurolide A1	C15H17NO5	323.1371	324.1444

185	Ecgonine methyl ester	C10H17NO3	199.121	200.1283
186	1-(2,4-diphenyl-2,3-dihydro-1H-1,5-benzodiazepin-1-yl)propan-1-one	C24H22N2O	376.1476	377.1548
187	Δ2-trans-Hexadecenoic acid	C16H30O2	254.2247	255.2319
188	β-Asarone	C12H16O3	208.1103	209.1176
189	Adenosine	C10H13N5O4	267.0968	268.1041
190	N,N-Diethylethanolamine	C6H15NO	117.1155	118.1228
191	N,5-Bis(3-nitrophenyl)oxazol-2-amine	C15H10N4O5	326.0615	327.0687
192	(2R)-2-[(2R,5S)-5-[(2S)-2-hydroxybutyl]oxolan-2-yl]propanoic acid	C11H20O4	216.1364	217.1436
193	3,5,7-trihydroxy-2-phenyl-4H-chromen-4-one	C15H10O5	270.053	271.0603
194	5-[(10Z)-14-(3,5-dihydroxyphenyl)tetradec-10-en-1-yl]benzene-1,3-diol	C26H36O4	434.2453	435.2525
195	2-Phenylglycine	C8H9NO2	151.0634	152.0707
196	DGDG O-9:0_14:0	C38H72O14	774.4747	775.4819
197	Oxymatrine	C15H24N2O2	264.1833	265.1906
198	5,5-dimethyl-3-morpholinocyclohex-2-en-1-one	C12H19NO2	209.1417	210.149
199	L-5-Hydroxytryptophan	C11H12N2O3	220.085	221.0922
200	11-Ketoetiocholanolone	C19H28O3	304.2037	305.2111
201	Tetrahydrobiopterin	C9H15N5O3	241.1176	242.1249
202	o-Toluic Acid	C8H8O2	136.0526	137.0599
203	Tomatidine	C27H45NO2	415.3452	416.3525
204	4-phenoxyphenyl 4-hydroxypiperidine-1-carboxylate	C18H19NO4	313.1345	336.1238
205	p-Mentha-1,3,8-triene	C10H14	134.1097	135.117
206	4-{3-[(3,4-dihydroxyphenyl)methyl]-2-methylbutyl}benzene-1,2-diol	C18H22O4	324.1341	325.1414
207	4-oxo-5-phenylpentanoic acid	C11H12O3	174.0682	175.0755
208	DLK	C16H30N4O6	374.2167	375.224
209	Guggulsterone	C21H28O2	312.2091	313.2164
210	2-Amino-1,3,4-octadecanetriol	C18H39NO3	317.293	318.3003
211	N6-Methyladenine	C6H7N5	149.0702	150.0775
212	Methyl cinnamate	C10H10O2	162.0683	163.0756
213	2,6-Di-tert-butyl-1,4-benzoquinone	C14H20O2	220.1466	221.1538
214	1,7-bis(4-hydroxyphenyl)heptan-3-one	C19H22O3	298.1605	299.1678
215	3-(dimethylamino)-2-[3-(trifluoromethyl)phenyl]acrylonitrile	C12H11F3N2	240.0901	241.0973
216	Irganox 259	C40H62O6	1352.802	677.4083
217	Threonine	C4H9NO3	119.0585	120.0657
218	Eucalyptol	C10H18O	136.1254	137.1326
219	2-Methoxybenzaldehyde	C8H8O2	136.0526	137.0599
220	N-Acetylputrescine	C6H14N2O	130.1108	131.1181
221	5-Aminosalicylic Acid	C7H7NO3	153.0426	154.0499

222	N1-(1-benzyl-4-piperidyl)-4-chlorobenzene-1-sulfonamide	C18H21ClN2O2S	364.0981	365.1054
223	2-oxopiperidine-3-carbohydrazide	C6H11N3O2	157.0852	158.0925
224	2-Methylhippuric acid	C10H11NO3	193.0716	194.0789
225	(3S,9aS)-3-benzyl-octahydro-1H-pyrido[1,2-a]pyrazin-1-one	C15H20N2O	266.1377	267.145
226	N-Methyldioctylamine	C17H37N	255.2928	256.3
227	L-Dopa	C9H11NO4	197.0691	198.0763
228	4-Phenylbutyric acid	C10H12O2	164.084	165.0913
229	13,14-Dihydro-15-keto Prostaglandin E2	C20H32O5	369.2517	370.259
230	5-(hydroxymethyl)-4-methoxy-2,5-dihydrofuran-2-one	C6H8O4	126.032	127.0392
231	1,7-bis(3,4-dihydroxyphenyl)heptan-3-one	C19H22O5	330.1444	331.1517
232	2-phenyl-4H-furo[2,3-h]chromen-4-one	C17H10O3	262.0664	263.0737
233	13,14-dihydro-15-keto-tetranor Prostaglandin E2	C16H26O5	320.16	321.1672
234	Arachidonic acid	C20H32O2	286.2298	287.2371
235	CAR 14:3	C21H36NO4	365.2567	366.264
236	11-Oxoetiocholanolone	C19H28O3	286.1933	287.2005
237	2-[(2-amino-6-methylpyrimidin-4-yl)thio]-4,6-dimethylnicotinonitrile	C13H13N5S	271.0854	272.0927
238	2,5-bis(4-hydroxy-3-methoxyphenyl)-3,4-dimethyloxolan-3-ol	C20H24O6	342.1468	343.1541
239	8-Hydroxyquinoline	C9H7NO	145.0529	146.0601
240	VLH	C17H29N5O4	367.222	368.2293
241	N,N-Dimethylaniline	C8H11N	121.0895	122.0968
242	N-(5-acetamidopentyl)acetamide	C9H18N2O2	208.1214	209.1287
243	Ala-Ile	C9H18N2O3	202.132	203.1393
244	Scopoletin	C10H8O4	192.0425	193.0498
245	LysoPC 12:1	C20H36NO7P	433.225	434.2323
246	N-Acetyl-S-allyl-L-cysteine	C8H13NO3S	203.062	226.0511
247	(2R,3S,4S,5R,6R)-2-(hydroxymethyl)-6-(propan-2-yloxy)oxane-3,4,5-triol	C9H18O6	260.0663	261.0736
248	Cafestol	C20H28O3	316.2038	317.2111
249	Desthiobiotin	C10H18N2O3	214.132	215.1392
250	15-epi Cloprostenol	C22H29ClO6	446.1554	447.1627
251	Vasicinone	C11H10N2O2	202.0743	203.0816
252	N-(4-fluorophenyl)-N'-(2-piperidinophenyl)urea	C18H20FN3O	335.1372	336.1445
253	3,4-Dihydroxybenzaldehyde	C7H6O3	138.0319	139.0392
254	7-Methyladenine	C6H7N5	149.0702	150.0775
255	Anserine	C10H16N4O3	240.1212	241.1295
256	(1E)-5-hydroxy-1,7-diphenylhept-1-en-3-one	C19H20O2	302.1251	303.1324
257	Genistein 4'-O-glucuronide	C21H18O11	446.0852	447.0925

258	2-imino-8-methoxy-2H-chromene-3-carbonitrile	C11H8N2O2	200.0587	201.066
259	N6-Acetyl-L-lysine	C8H16N2O3	188.1162	189.1235
260	3-Methylhistamine	C6H11N3	125.0956	126.1028
261	2-[(5-anilino-4-phenyl-4H-1,2,4-triazol-3-yl)thio]acetic acid	C16H14N4O2S	326.0829	327.0902
262	(12Z)-9,10,11-trihydroxyoctadec-12-enoic acid	C18H34O5	352.2232	353.2302
263	4-phenyl-6-(2-thienyl)-2,3,4,5-tetrahydropyridazin-3-one	C14H12N2OS	256.067	257.0743
264	4-Ethylbenzaldehyde	C9H10O	134.0734	135.0806
265	Sedanolide	C12H18O2	194.1308	195.1381
266	2-acetamido-3-(4-methoxyphenyl)propanoic acid	C12H15NO4	237.1003	238.1077
267	N1-(2-amino-2-oxoethyl)pent-3-enamide	C7H12N2O2	156.09	157.0973
268	2-(4-chlorophenyl)-2-oxoethyl 2,6-bis(trifluoromethyl)benzoate	C17H9ClF6O3	410.0106	411.0179
269	(S)-Leucic acid	C6H12O3	132.0789	133.0861
270	3-(3,4,5-trimethoxyphenyl)propanoic acid	C12H16O5	262.0818	263.0891
271	6 β-Hydroxycortisol	C21H30O6	378.2042	379.2114
272	Radicinin	C12H12O5	236.0663	237.0736
273	Benzamide	C7H7NO	121.0531	122.0603
274	4-(3,4-dimethoxyphenyl)-3-methyl-1H-pyrazol-5-amine	C12H15N3O2	233.1165	234.1238
275	Octyl hydrogen phthalate	C16H22O4	278.1507	279.1579
276	4-methyl-6-phenyl-5,6-dihydro-2H-pyran-2-one	C12H12O2	188.0838	189.0911
277	3-amino-4-(propylamino)cyclobut-3-ene-1,2-dione	C7H10N2O2	154.0743	155.0816
278	1-hydroxy-1-(4-methoxyphenyl)propan-2-yl 4-methoxybenzoate	C18H20O5	298.1182	299.1255
279	1H-indol-3-yl(pyridin-2-yl)methanol	C14H12N2O	206.0845	207.0918
280	Kahweol	C20H26O3	314.188	315.1954
281	YLK	C21H34N4O5	382.2572	405.2464
282	Phosphocholine	C5H14NO4P	183.0663	184.0736
283	5α-Pregnan-3,20-dione	C21H32O2	316.2404	317.2477
284	Bicyclo Prostaglandin E2	C20H30O4	356.1946	357.2016
285	YKK	C21H35N5O5	1749.032	875.5232
286	1-(3,4-dihydroxyphenyl)-7-(4-hydroxyphenyl)heptan-3-one	C19H22O4	336.1365	337.1437
287	Carvone	C10H14O	150.1046	151.1119
288	Gly-Phe	C11H14N2O3	222.1006	223.1079
289	5,6-dimethyl-4-oxo-4H-pyran-2-carboxylic acid	C8H8O4	168.0399	169.0472
290	Andrographolide	C20H30O5	350.2094	351.2167
291	2-(14,15-Epoxyeicosatrienoyl) glycerol	C23H38O5	416.2542	417.2614
292	ethyl 2-(5-tetrahydro-1H-pyrrol-1-yl-2H-1,2,3,4-tetraazol-2-yl)acetate	C9H15N5O2	225.1229	226.1302

293	ELK	C17H32N4O6	388.2319	389.2395
294	8-Isoprostaglandin F1β	C20H36O5	373.2827	374.29
295	3-Methoxytyramine	C9H13NO2	167.0946	168.1019
296	cis-7-Hexadecenoic Acid	C16H30O2	254.2245	255.2318
297	Hydroquinone	C6H6O2	110.0371	111.0445
298	CAR 13:0	C20H40NO4	357.2881	358.2954
299	O-Desmethylnaproxen	C13H12O3	216.0789	217.0861
300	4,6,8-trihydroxy-7-methoxy-3-methyl-3,4-dihydro-1H-2-benzopyran-1-one	C11H12O6	262.0448	263.0521
301	L-Kynurenine	C10H12N2O3	208.085	209.0923
302	5-acetyl-2,6-dimethyl-1,2,3,4-tetrahydropyridin-4-one	C9H13NO2	167.0947	168.102
303	9-HOTrE	C18H30O3	294.2195	295.2266
304	1,2-di(3,4-dimethoxyphenyl)diaz-1-ene	C16H18N2O4	638.299	320.1568
305	Phenylethanolamine	C8H11NO	137.0843	138.0915
306	(±)11(12)-DiHET	C20H34O4	361.1737	362.1808
307	Papaverine	C20H21NO4	339.1501	340.1574
308	4-Aminobutyric acid	C4H9NO2	103.0638	104.0711
309	(11E,15Z)-9,10,13-trihydroxyoctadeca-11,15-dienoic acid	C18H32O5	345.2519	346.2592
310	Methionine	C5H11NO2S	149.0511	150.0584
311	3-hydroxy-3-methylpentanedioic acid	C6H10O5	184.0349	185.0422
312	Shikonin	C16H16O5	270.0892	271.0965
313	3-(1-naphthylmethylidene)indolin-2-one	C19H13NO	271.0992	272.1065
314	6-Hydroxynicotinic acid	C6H5NO3	139.0269	140.0341
315	Nicotinamide	C6H6N2O	122.0481	123.0556
316	P-Acetamidophenyl-b-D-glucuronide	C14H17NO8	344.1214	345.1287
317	Styrene	C8H8	104.0631	105.0703
318	5-[3-(2-Chloro-4-fluorobenzyl)-1,2,4-oxadiazol-5-yl]-3-pyrrolidinol	C13H13ClFN3O2	297.0649	298.0721
319	Thromboxane B1	C20H36O6	394.2335	395.2408
320	2-(4-chlorophenoxy)pyridin-3-amine	C11H9ClN2O	220.0384	203.0351
321	2-((Dimethylamino)methyl)phenol	C9H13NO	151.1	152.1073
322	Isoproterenol	C11H17NO3	211.121	212.1283
323	methyl 3,4,5-trihydroxycyclohex-1-ene-1-carboxylate	C8H12O5	210.0506	211.0578
324	2-(2,6-dimethoxyphenyl)-5,6-dimethoxy-4H-chromen-4-one	C19H18O6	324.0996	325.1069
325	Puerarin	C21H20O9	416.1107	417.118
326	DL-Panthenol	C9H19NO4	205.1317	206.139
327	Deoxyinosine	C10H12N4O4	252.086	253.0932
328	Cortisone	C21H28O5	360.1937	361.2009
329	N-Acetyl-L-methionine	C7H13NO3S	191.0617	192.069
330	2-Isopropylaniline	C9H13N	135.105	136.1122

331	N'-hydroxy-2-methyl-1,3-thiazole-4-carboximidamide	C5H7N3OS	179.0139	180.0211
332	2-(3-chloro-2-methylanilino)benzoic acid	C14H12ClNO2	261.0557	262.063
333	Pipecolic acid	C6H11NO2	147.0897	130.0864
334	(3,4-Dimethoxyphenyl)acetic acid	C10H12O4	196.0739	197.0812
335	L-Methionine Methyl Ester	C6H13NO2S	163.0668	164.0741
336	Vindoline	C25H32N2O6	912.4538	457.2342
337	2-Aminobutyric acid	C4H9NO2	103.0638	104.0711
338	1,2-dihydroxyheptadec-16-yn-4-yl acetate	C19H34O4	343.2724	344.2797
339	5-Methylcytosine	C5H7N3O	125.0591	126.0664
340	RPH	C17H28N8O4	408.2263	409.2335
341	2-(3,4-dimethoxyphenyl)ethanamine	C10H15NO2	181.1104	182.1176
342	All trans-Retinal	C20H28O	284.214	285.2213
343	methyl 2-(2-acetyl-4,5-dimethoxyphenyl)acetate	C13H16O5	252.1002	253.1074
344	4-acetyl-4-(ethoxycarbonyl)heptanedioic acid	C12H18O7	274.1032	275.1105
345	Kinetin	C10H9N5O	215.0797	216.0869
346	1-(7-methoxy-2-oxo-2H-chromen-8-yl)-3-methyl-2-oxobutyl acetate	C17H18O6	313.1523	314.1595
347	DMK	C15H28N4O6S	392.1813	393.1886
348	Thromboxane B2	C20H34O6	392.2174	393.2247
349	6-methyl-7-nitro-2,3-dihydro-1,4-benzodioxine	C9H9NO4	177.0428	178.0501
350	3-(3,4-dihydroxyphenyl)propanoic acid	C9H10O4	164.0478	165.0548
351	4-methoxy-6-(prop-2-en-1-yl)-2H-1,3-benzodioxole	C11H12O3	192.079	193.0863
352	Prostaglandin I2	C20H32O5	352.2251	353.2324
353	Nicotinate ribonucleoside	C11H13NO6	255.0741	256.0814
354	L-(-)-alpha-Amino-epsilon-Caprolactam	C6H12N2O	128.0951	129.1024
355	5-(6-hydroxy-6-methyloctyl)-2,5-dihydrofuran-2-one	C13H22O3	208.1466	209.1539
356	DGDG O-19:2_16:0	C50H92O14	938.6161	939.6237
357	4-methylphenyl 1-ethyl-3-methyl-1H-pyrazole-5-carbothioate	C14H16N2OS	260.1027	261.1099
358	11β-Hydroxyandrosterone	C19H30O3	306.2195	307.2268
359	Tyramine	C8H11NO	137.0844	138.0915
360	3,3-dimethyl-2-morpholino-2,3-dihydrobenzo[b]furan-5-ol	C14H19NO3	249.1366	250.1438
361	N-(5-Aminopentyl)acetamide	C7H16N2O	144.1264	145.1336
362	2,2-dimethyl-6,7-di[(4-nitrobenzyl)oxy]chroman-4-one	C25H22N2O8	478.1453	479.1525
363	4-Hydroxybenzoic acid	C7H6O3	138.032	139.0392
364	L-arginine	C6H14N4O2	174.1119	175.1192
365	1-(piperidinomethyl)-2-naphthol	C16H19NO	241.1507	242.158
366	YQH	C20H26N6O6	468.1746	469.1819
367	6-Methoxy-2-naphthoic acid	C12H10O3	202.0633	203.0705
368	(+)-ar-Turmerone	C15H20O	216.1515	217.1588

369	1-(4-nitrophenyl)piperidine	C11H14N2O2	206.1057	207.113
370	Glycylproline	C7H12N2O3	172.0849	173.0922
371	Cortisol	C21H30O5	362.2091	363.2165
372	Ouabain	C29H44O12	566.2607	567.268
373	5-Phenylvaleric Acid	C11H14O2	178.0996	179.1069
374	Cnidioside A	C17H20O9	390.0928	391.1001
375	Eicosapentaenoic acid	C20H30O2	302.2246	303.2319
376	5-phenyl-2,3-dihydro-1H-1,4-benzodiazepin-2-one	C15H12N2O	274.0522	275.0595
377	N-Benzyloxycarbonylglycine	C10H11NO4	209.0691	210.0763
378	1-Acetylimidazole	C5H6N2O	110.0484	111.0557
379	2-(1H-indol-3-yl)acetic acid	C10H9NO2	175.0635	176.0708
380	Prostaglandin D1	C20H34O5	371.2672	372.2745
381	N1-[2-oxo-6-(1H-pyrrol-1-yl)-2H-chromen-3-yl]acetamide	C15H12N2O3	268.0883	269.0955
382	ERH	C17H28N8O6	440.2169	441.2242
383	2-(4-chlorophenyl)-5-(4-phenylbuta-1,3-dienyl)-2H-1,2,3,4-tetraazole	C17H13ClN4	308.0746	309.0819
384	Etiocholanolone	C19H30O2	272.2142	273.2214
385	N-[2,5-bis(2,2,2-trifluoroethoxy)benzoyl]-N'-(4-methoxyphenyl)urea	C19H16F6N2O5	466.0945	467.1018
386	Adenine	C5H5N5	135.0546	136.0619
387	Bisphenol TMC	C21H26O2	327.2158	328.2231
388	1-butyl-2-methyl-4-nitro-1H-imidazole	C8H13N3O2	183.1011	184.1083
389	Triethanolamine	C6H15NO3	149.1054	150.1127
390	2-phenyl[1,3]oxazolo[4,5-c]quinolin-4(5H)-one	C16H10N2O2	262.0745	263.0818
391	1-(4-benzhydrylpiperidino)-2,2,2-trichloroethan-1-one	C20H20Cl3NO	395.0651	396.0724
392	17alpha-Hydroxyprogesterone	C21H30O3	330.2193	331.2266
393	Biotin	C10H16N2O3S	244.0881	227.0848
394	2-phenyl-2,4,6,7-tetrahydrothiino[4,3-c]pyrazol-3-ol	C12H12N2OS	232.0688	233.0761
395	Methyl jasmonate	C13H20O3	224.1414	225.1487
396	Artesunate	C19H28O8	406.1606	407.1679
397	3-[4-(3,5-dichloro-4-pyridinyl)piperazino]-1,1,1-trifluoro-2-propanol	C12H14Cl2F3N3O	343.0516	344.0589
398	tetranor-12(R)-HETE	C16H26O3	248.1778	249.1851
399	Spectinomycin	C14H24N2O7	364.1824	365.1896
400	4-[4-(2,5-dimethyl-1H-pyrrol-1-yl)phenyl]-6-fluoro-4-thiochromanol	C21H20FNOS	353.1275	354.1348
401	Methyl 3-indolyacetate	C11H11NO2	189.0791	190.0864
402	Jasmone	C11H16O	164.1202	165.1275
403	trans-4-Hydroxy-L-proline	C5H9NO3	131.0585	132.0657
404	3-(tert-butyl)-1-methyl-4,5-dihydro-1H-pyrazol-5-one	C8H14N2O	154.1107	155.118

405	D-Glucosamine 6-phosphate	C6H14NO8P	259.0457	260.0529
406	YNH	C19H24N6O6	432.1763	433.1836
407	Tetramethylpyrazine	C8H12N2	136.1002	137.1075
408	1,7-bis(4-hydroxyphenyl)-5-methoxyheptan-3-one	C20H24O4	350.151	351.1583
409	DL-5-Methoxytryptophan	C12H14N2O3	252.111	253.118
410	Valdecoxib	C16H14N2O3S	314.0725	315.0797
411	N,N'-di[4-(2,6-dimethylmorpholino)phenyl]thiourea	C25H34N4O2S	476.2237	477.231
412	N-Acetyl-L-cysteine	C5H9NO3S	163.0304	164.0377
413	3-morpholino-5,6-diphenylpyridazine-4-carbonitrile	C21H18N4O	342.1473	343.1546
414	1-methyl-3,5-di(1-naphthylmethylidene)piperidin-4-one	C28H23NO	285.1212	286.1285
415	7α-Hydroxytestosterone	C19H28O3	304.2036	305.2109
416	Menadione	C11H8O2	172.0526	173.0599
417	2-[(butylamino)(imino)methyl]-1-oxohydrazinium-1-olate	C5H12N4O2	182.0735	183.0807
418	3-(tert-butyl)-1-methyl-N-(2-oxo-3-azepanyl)-1H-pyrazole-5-carboxamide	C15H24N4O2	292.19	293.1972
419	L-Carnitine	C7H15NO3	161.1053	162.1126
420	PLK	C17H32N4O4	356.2425	357.2498
421	L-Theanine	C7H14N2O3	174.1006	175.1079
422	Kanosamine	C6H13NO5	179.0809	180.0882
423	4-Guanidinobutanoic acid	C5H11N3O2	145.0853	146.0926
424	3,7-Dimethyluric acid	C7H8N4O3	196.0599	197.0671
425	PMK	C16H30N4O4S	170.0692	171.0765
426	(2R,3S,4S,5R,6R)-2-(hydroxymethyl)-6-(2-phenylethoxy)oxane-3,4,5-triol	C14H20O6	301.1524	302.1597
427	4-azocan-1-yl-5,7-dichloro-2-(trifluoromethyl)quinoline	C17H17Cl2F3N2	376.0775	377.0848
428	Morphine-3-glucuronide	C23H27NO9	461.1685	462.1757
429	Leukotriene E4	C23H37NO5S	439.2396	440.2469
430	3-(5-phenyl-1,3-oxazol-2-yl)-4-(trifluoromethyl)pyridine	C15H9F3N2O	290.0623	291.0696
431	1-(4-methyl-2-morpholino-1,3-thiazol-5-yl)ethan-1-one	C10H14N2O2S	226.0819	227.0892
432	N-(2-hydroxy-2-phenylethyl)-N'-(2-thienyl)urea	C13H14N2O2S	262.0821	263.0893
433	Norverapamil	C26H36N2O4	440.2677	441.275
434	Quercitrin	C21H20O11	470.0827	471.09
435	Epitestosterone	C19H28O2	288.2091	289.2163
436	Cyclocytidine	C9H11N3O4	225.0751	226.0824
437	4-Quinolinecarboxylic acid	C10H7NO2	173.0476	174.0551
438	Phenylpyruvic acid	C9H8O3	164.0466	329.0994
439	6-benzyl-4-oxo-1,4-dihydropyridine-3-carboxamide	C13H12N2O2	250.0755	251.0828

440	2-(piperidinomethylidene)malononitrile	C9H11N3	161.0954	162.1026
441	8,8-dimethyl-2-phenyl-4H,8H-pyrano[2,3-h]chromen-4-one	C20H16O3	304.1135	305.1208
442	5,6-dimethoxy-2-(2-methoxyphenyl)-4H-chromen-4-one	C18H16O5	334.0817	335.089
443	1-(3,4-dimethoxyphenyl)ethan-1-one oxime	C10H13NO3	195.0892	196.0965
444	AKB48 N-(5-hydroxypentyl) metabolite	C23H31N3O2	403.2207	404.228
445	o-Veratraldehyde	C9H10O3	166.0624	167.0696
446	N-(1-benzothiophen-2-yl)-N'-(2-methylphenyl)urea	C16H14N2OS	304.0638	305.0711
447	Guanine	C5H5N5O	151.0494	152.0567
448	(2-morpholino-5-nitrophenyl)methanol	C11H14N2O4	238.0932	239.1005
449	2-(2-acetyl-3,5-dihydroxyphenyl)acetic acid	C10H10O5	192.0425	193.0498
450	3-(4-hydroxy-3-methoxyphenyl)propanoic acid	C10H12O4	218.0558	219.063
451	3,4,5-trihydroxy-6-methyloxan-2-yl 2-(methylamino)benzoate	C14H19NO6	297.1214	298.1287
452	Dehydroepiandrosterone (DHEA)	C19H28O2	270.1986	271.2058
453	ethyl 1-(3-nitro-2-thienyl)piperidine-4-carboxylate	C12H16N2O4S	266.0767	267.084
454	N-Palmitoyl taurine	C18H37NO4S	363.2412	364.2485
455	3-benzyl-1-butyl-4-hydroxy-1,2-dihydroquinolin-2-one	C20H21NO2	345.1061	346.1134
456	(-)-Caryophyllene oxide	C15H24O	220.183	203.1797
457	5-Methoxyindoleacetic acid	C11H11NO3	205.0733	206.081
458	Prostaglandin E2-1-glyceryl ester	C23H38O7	443.2852	444.2925
459	Cinchophen	C16H11NO2	249.0791	250.0864
460	2-morpholino-1-phenyl-1-ethanol	C12H17NO2	189.1157	190.123
461	4-Methoxycinnamic acid	C10H10O3	178.0631	161.0598
462	Valylproline	C10H18N2O3	214.1319	215.1391
463	1-Aminocyclohexanecarboxylic acid	C7H13NO2	143.0948	144.1021
464	morpholino(quinolin-6-yl)methanone	C14H14N2O2	242.1035	243.1108
465	1-Phenyl-3-methyl-5-pyrazolone	C10H10N2O	174.0795	175.0868
466	1-[3-nitro-4-(2-pyridylthio)phenyl]ethan-1-one	C13H10N2O3S	274.041	275.0483
467	2-methyl-2,3,4,5-tetrahydro-1,5-benzoxazepin-4-one	C10H11NO2	159.0686	160.0759
468	2-(4-methoxyphenyl)hydrazine-1-carbothioamide	C8H11N3OS	197.0632	198.0705
469	N1-[4-hydroxy-6-(methoxymethyl)pyrimidin-2-yl]acetamide	C8H11N3O3	197.0802	198.0874
470	2-methyl-6-{[(5-phenyl-2-thienyl)carbonyl]amino}benzoic acid	C19H15NO3S	359.0548	360.062
471	4-[4-(methoxymethyl)-6-piperidinopyrimidin-2-yl]-2-methyl-1,3-thiazole	C15H20N4OS	304.1399	305.1472
472	5-(2,5-dihydroxyhexyl)oxolan-2-one	C10H18O4	184.1102	185.1175
473	HPK	C17H28N6O4	380.218	381.2253
474	Methionine sulfoxide	C5H11NO3S	165.0461	166.0534

475	L-Histidinol	C6H11N3O	141.0904	142.0977
476	N2-tetrahydrofuran-2-ylmethyl-4-(4-fluorophenyl)-1,3-thiazol-2-amine	C14H15FN2OS	278.0943	301.0835
477	4-Butylresorcinol	C10H14O2	166.0996	149.0962
478	1-(4-methoxyphenyl)propane-1,2-diol	C10H14O3	164.0839	165.0912
479	6-methyl-4-(morpholinomethyl)-2H-chromen-2-one	C15H17NO3	259.1211	260.1284
480	3-Methylcrotonylglycine	C7H11NO3	157.074	158.0813
481	1-(6-methyl-3-pyridyl)ethan-1-one O1-ethyloxime hydrochloride	C10H14N2O	178.1108	179.118
482	5-(2,4-difluorophenyl)-2-furaldehyde oxime	C11H7F2NO2	223.0445	224.0518
483	Acipimox	C6H6N2O3	154.0379	155.0452
484	Homoserine	C4H9NO3	119.0585	120.0657
485	N1-(2,3-dihydro-1,4-benzodioxin-2-ylmethyl)-2,2-dimethylpropanamide	C14H19NO3	249.1344	250.1416
486	1-(3-phenylpropanoyl)-4-piperidinecarboxylic acid	C15H19NO3	261.1328	262.1401
487	N-(8-methyl-8-azabicyclo[3.2.1]oct-3-yl)-4-nitrobenzamide	C15H19N3O3	289.1385	290.1458
488	Terephthalic Acid	C8H6O4	166.027	167.0343
489	4-methyl-5-oxo-2-pentyl-2,5-dihydrofuran-3-carboxylic acid	C11H16O4	212.1029	213.1101
490	N-(9-oxodecyl)acetamide	C12H23NO2	251.127	252.1343
491	Thr-Leu	C10H20N2O4	232.1424	215.1392
492	N-(2,4-Dimethylphenyl)formamide	C9H11NO	149.0842	150.0915
493	N-Arachidonoyl-L-serine	C23H37NO4	391.2724	392.2797
494	N-Acetylhistidine	C8H11N3O3	215.0908	198.0875
495	6-(dimethylamino)-2-phenyl-1H-benzo[de]isoquinoline-1,3(2H)-dione	C20H16N2O2	316.1206	317.1278
496	Riboflavin-5-phosphate	C17H21N4O9P	456.1033	457.1105
497	3-(3-morpholinopropyl)-2-(2-pyridinyl)-2,3-dihydro-4(1H)-quinazolinone	C20H24N4O2	352.1862	353.1935
498	3-amino-2-phenyl-2H-pyrazolo[4,3-c]pyridine-4,6-diol	C12H10N4O2	242.0805	243.0878
499	3-[(2-pyridylthio)methyl]-4,5-dihydro-1H-pyrazol-5-one	C9H9N3OS	229.0294	230.0367
500	dAMP	C10H14N5O6P	331.0668	332.074
501	4-[4-phenyl-3,6-dihydro-1(2H)-pyridinyl]butanoic acid hydrochloride	C15H19NO2	245.1415	246.1488
502	Oxoamide	C10H12N2O2	192.09	193.0973
503	4-Hydroxyquinoline	C9H7NO	145.0563	146.0636
504	Tyrosylalanine	C12H16N2O4	252.1088	253.1161
505	Benzamidine	C7H8N2	120.069	121.0763
506	(2R)-5-hydroxy-7-methoxy-2-phenyl-3,4-dihydro-2H-1-benzopyran-4-one	C16H14O4	270.0892	271.0965

507	TQH	C15H24N6O6	384.1762	385.1835
508	1-(3-ethyl-2,4-dihydroxy-6-methoxyphenyl)butan-1-one	C13H18O4	220.1077	221.1149
509	3-[4-methyl-1-(2-methylpropanoyl)-3-oxocyclohexyl]butanoic acid	C15H24O4	306.1193	307.1266
510	Doxycycline	C22H24N2O8	444.1525	445.1598
511	N-(1-methyl-3-phenyl-1H-pyrazol-5-yl)-N'-(2-thienyl)urea	C15H14N4OS	336.0458	337.0531
512	2,3,4-Trihydroxybenzoic acid	C7H6O5	170.0215	171.0288
513	1,3-Dihydro-1,3,3-trimethyl-2H-indol-2-ylidene acetaldehyde	C13H15NO	201.1155	202.1228
514	7-(2-aminophenyl)heptanoic acid	C13H19NO2	221.1419	222.1492
515	8-(1,2-dihydroxy-3-methylbut-3-en-1-yl)-7-methoxy-2H-chromen-2-one	C15H16O5	276.0977	277.105
516	N-(2-hydroxyphenyl)acetamide	C8H9NO2	173.0438	174.0511
517	Cryptotanshinone	C19H20O3	296.1378	297.145
518	3-(1-benzylpiperidin-4-yl)-3H-[1,2,3]triazolo[4,5-b]pyridine	C17H19N5	293.1629	294.1701
519	N-Desmethyltramadol	C15H23NO2	249.1732	250.1805
520	4-morpholinobenzoic acid	C11H13NO3	207.0897	208.097
521	8,8-dimethyl-2H,8H-pyrano[3,2-g]chromen-2-one	C14H12O3	228.0787	229.086
522	Thymidine	C10H14N2O5	242.0903	243.0976
523	ADBICA N-pentanoic acid metabolite	C20H27N3O4	411.1572	412.1644
524	2-(1-adamantyl)-1-morpholinoethan-1-one	C16H25NO2	263.1863	264.1936
525	D-(+)-Tryptophan	C11H12N2O2	204.0902	205.0974
526	S-Adenosylhomocysteine	C14H20N6O5S	384.1216	385.1288
527	Avobenzone	C20H22O3	326.1296	349.1191
528	5-OxoETE	C20H30O3	318.2189	319.2262
529	1-(4-Methoxyphenyl)-2-propanone	C10H12O2	164.0839	182.1177
530	2-methyl-1,2-dihydrophthalazin-1-one	C9H8N2O	160.0638	161.0711
531	Kinetin 9-riboside	C15H17N5O5	347.122	348.1293
532	Senecionine	C18H25NO5	335.173	336.1803
533	JWH 018 N-(4,5-epoxypentyl) analog	C24H21NO2	355.1635	356.1707
534	Cannabicitran	C21H30O2	314.225	315.232
535	1-(1,8-dihydroxy-3,6-dimethyl-2-naphthyl)ethan-1-one	C14H14O3	230.0946	231.1019
536	Carbaprostacyclin	C21H34O4	332.2365	333.2438
537	Isoquinoline	C9H7N	129.0579	130.0651
538	DL-Citrulline	C6H13N3O3	175.0958	176.103
539	2-[5-(2-hydroxypropyl)oxolan-2-yl]propanoic acid	C10H18O4	224.1026	225.1099
540	2-[2-oxo-2-(2-pyridylamino)ethoxy]acetic acid	C9H10N2O4	210.0642	211.0714
541	Guvacoline	C7H11NO2	141.0792	142.0865

542	Pantethine	C22H42N4O8S2	554.2469	555.2542
543	N-P-Coumaroyl Spermidine	C16H25N3O2	291.1947	292.202
544	L-Threonine	C4H9NO3	119.0585	120.0658
545	2-{2-[(1-methyl-1H-pyrazol-5-yl)amino]-2-oxoethoxy}acetic acid	C8H11N3O4	235.0591	236.0664
546	SPH	C14H21N5O5	678.3089	340.1617
547	Diphenylamine	C12H11N	169.0892	170.0965
548	3-Acetyl-2,5-dimethylfuran	C8H10O2	138.0683	139.0755
549	ethyl 4-hydroxy-2-[(4-methoxyphenoxy)methyl]pyrimidine-5-carboxylate	C15H16N2O5	342.0561	343.0633
550	2-Naphthol	C10H8O	144.0577	145.065
551	S-Adenosylmethionine	C15H22N6O5S	398.1371	399.1443
552	5-(benzyloxy)-2-(hydroxymethyl)-1,4-dihydropyridin-4-one	C13H13NO3	231.0895	232.0968
553	N-isopropyl-N'-(2-oxoazepan-3-yl)urea	C10H19N3O2	213.1479	214.1552
554	4-Methylumbelliferyl glucuronide	C16H16O9	352.0772	353.0845
555	4-OHE1-1-N3Ade	C23H25N5O3	419.1923	420.1996
556	WPH	C22H26N6O4	876.3982	439.2061
557	3-Indoleacetonitrile	C10H8N2	156.0692	157.0762
558	T-2 Toxin	C24H34O9	488.2026	489.2099
559	Clinafloxacin	C17H17ClFN3O3	387.0729	388.0801
560	Sodium cholate	C24H39NaO5	430.2696	431.2769
561	RMK	C17H35N7O4S	415.2396	416.2469
562	Metronidazole-OH	C6H9N3O4	187.0595	188.0668
563	11-dehydro Thromboxane B2	C20H32O6	368.2179	369.2251
564	Phenylpropiolic acid	C9H6O2	146.037	147.0443
565	5-Hydroxylysine	C6H14N2O3	162.1006	163.1079
566	H-Tyr(3-I)-OH	C9H10INO3	306.9699	307.9772
567	4-(cyclohexylmethyl)-6-(2-thienyl)-2,3-dihydropyridazin-3-one hydrate	C15H18N2OS	274.1182	275.1255
568	DL-3,4-Dihydroxyphenyl glycol	C8H10O4	170.0582	171.0654
569	4-Pregnen-17alpha,20alpha-Diol-3-One	C21H32O3	332.2355	333.2427
570	(2,6-dimethylmorpholino)(1-methyl-5-nitro-1H-pyrazol-4-yl)methanone	C11H16N4O4	290.099	291.1063
571	Dl-3-Hydroxy-kynurenine	C10H12N2O4	224.0796	225.0869
572	2-oxa-4-azatetracyclo[6.3.1.1~6,10~.0~1,5~]tridecan-3-one	C11H15NO2	193.1106	194.1179
573	5-chloro-3-phenylbenzo[c]isoxazole	C13H8ClNO	229.0294	230.0367
574	2-Thio-acetyl MAGE	C21H42O3S	396.2643	397.2716
575	4-(pentyloxy)benzene-1-carbohydrazide	C12H18N2O2	222.1368	223.1441
576	Choline Glycerophosphate	C8H20NO6P	257.1027	258.1099
577	Secnidazole	C7H11N3O3	185.0802	186.0874

578	3-Hydroxypicolinic acid	C6H5NO3	139.0271	140.0344
579	Metanephrine	C10H15NO3	197.1053	198.1125
580	(1E,4E)-1,5-bis(4-methoxyphenyl)penta-1,4-dien-3-one	C19H18O3	316.106	317.1133
581	Lysopc 20:4	C28H50NO7P	543.3348	544.342
582	2-[(5-chloro-3-pyridyl)oxy]-5-(1H-pyrrol-1-yl)pyridine	C14H10ClN3O	271.0491	272.0564
583	s7p	C7H15O10P	290.0405	291.0477
584	3-methyl-5-oxo-5-(4-toluidino)pentanoic acid	C13H17NO3	257.103	258.1103
585	N1-[4-(trifluoromethyl)phenyl]-2-phenylbutanamide	C17H16F3NO	307.1144	308.1217
586	RMH	C17H30N8O4S	464.1959	465.2032
587	Feruloyl Putrescine	C14H20N2O3	264.1474	265.1547
588	Quinolinic acid	C7H5NO4	167.022	168.0292
589	Palmitoleic Acid	C16H30O2	276.2088	277.2161
590	Guanosine monophosphate	C10H14N5O8P	363.0566	364.0639
591	Obacunoic acid	C26H32O8	472.2076	473.2149
592	Phe-Pro	C14H18N2O3	262.1317	263.139
593	2-[2-(1-adamantylmethylidene)hydrazono]-5-methyl-1,3-thiazolan-4-one	C15H21N3OS	291.1446	292.1519
594	Pyridoxal 5'-Phosphate	C8H10NO6P	247.0249	248.0322
595	Diflucortolone pivalate	C27H36F2O5	478.2542	479.2615
596	L-beta-Imidazolelactic acid	C6H8N2O3	156.0537	174.0875
597	VLK	C17H34N4O4	358.2557	359.263
598	5,6-Dihydroxyindole-2-Carboxylic Acid	C9H7NO4	193.0377	194.045
599	N-Acetyl-D-galactosamine	C8H15NO6	221.0902	222.0975
600	12(S)-HETE	C20H32O3	320.2352	321.2425
601	Artemisinin	C15H22O5	264.1363	265.1436
602	Biopterin	C9H11N5O3	237.0863	238.0936
603	3,4-dihydroxy-4-(4-methoxyphenyl)-1,2,3,4-tetrahydroquinolin-2-one	C16H15NO4	267.0846	268.0919
604	1-(4-chlorophenyl)-1-phenyl-2-(3-pyridyl)ethan-1-ol	C19H16ClNO	309.0936	310.1009
605	4-(3-methyl-1-phenyl-1H-pyrazolo[3,4-b]quinolin-4-yl)morpholine	C21H20N4O	344.1599	345.1672
606	(2-methylcyclopentyl)(phenyl)methanone oxime	C13H17NO	203.1313	204.1386
607	7-Ketolithocholic acid	C24H38O4	390.2772	391.2845
608	4-Hydroxy-3-methylbenzoic acid	C8H8O3	152.0475	153.0548
609	5-Hydroxytryptophol	C10H11NO2	177.079	178.0863
610	Arachidonic acid methyl ester	C21H34O2	318.2562	319.2634
611	4-Acetamidobutyric Acid	C6H11NO3	145.0736	146.0809
612	Dl-Indole-3-lactic acid	C11H11NO3	205.0742	206.0815
613	Lysope 16:0	C21H44NO7P	453.286	454.2933

614	1-{4-methoxy-3-[(2-pyridylthio)methyl]phenyl}ethan-1-one	C15H15NO2S	273.0845	274.0917
615	2,6-Dihydroxypurine	C5H4N4O2	152.0337	153.041
616	alpha-Farnesene	C15H24	204.1879	205.1952
617	Phenmetrazine	C11H15NO	177.1156	178.1229
618	N~1~-(2,5-dimethoxyphenyl)-N~2~-(2-pyridin-2-ylethyl)ethanediamide	C17H19N3O4	351.1233	352.1305
619	4-methoxy-6-[2-(4-methoxyphenyl)ethyl]-2H-pyran-2-one	C15H16O4	282.0897	283.097
620	Tetranor-12(S)-HETE	C16H26O3	248.1779	249.1852
621	2-Methoxyestradiol (2-MeOE2)	C19H26O3	302.1885	303.1958
622	CAR 11:0	C18H36NO4	329.2564	330.2637
623	N-{2-[(2-furylmethyl)thio]ethyl}-2-[(3-methoxyphenyl)thio]acetamide	C16H19NO3S2	337.0798	338.0871
624	Spermidine	C7H19N3	145.1581	146.1654
625	Histamine	C5H9N3	111.0802	112.0874
626	1-adamantyl(1,4-thiazinan-4-yl)methanone	C15H23NOS	265.1527	266.16
627	Ala-trp	C14H17N3O3	275.1273	276.1346
628	Bz-RS-ISer(3-Ph)-Ome	C17H17NO4	299.1162	300.1235
629	Melatonin	C13H16N2O2	232.1214	233.1287
630	6-Hydroxymelatonin	C13H16N2O3	248.1162	249.1235
631	7-Ketocholesterol	C27H44O2	400.3344	401.3416
632	1-[(3,5-dimethylisoxazol-4-yl)sulfonyl]piperidine	C10H16N2O3S	244.0879	245.0952
633	Folinic acid	C20H23N7O7	473.1659	474.1732
634	2,6-Xylidine	C8H11N	121.0895	122.0967
635	2-[(3S)-1-(Benzylsulfonyl)-3-pyrrolidinyl]-1-methyl-1H-benzimidazole	C19H21N3O2S	377.1106	378.1178
636	7-hydroxy-5-methoxy-2-phenyl-3,4-dihydro-2H-1-benzopyran-4-one	C16H14O4	292.0671	293.0744
637	AB-PINACA N-(2-fluoropentyl) isomer	C18H25FN4O2	348.2028	349.2101
638	3-hydroxy-1,5-diphenylpentan-1-one	C17H18O2	276.1133	277.1206
639	methyl 6-{[4-(trifluoromethyl)anilino]carbonyl}nicotinate	C15H11F3N2O3	306.0578	307.0651
640	beta-Nicotinamide mononucleotide	C11H15N2O8P	334.0582	335.0654
641	Fmoc-L-Isoleucine	C21H23NO4	353.1632	354.1705
642	GPH	C13H19N5O4	331.1219	332.1292
643	D-Phenylalanine	C9H11NO2	165.0792	166.0865
644	Creatinine	C4H7N3O	113.0591	114.0664
645	N-Phenylacetylglutamine	C13H16N2O4	264.111	265.1183
646	Gamma-Caprolactone	C6H10O2	114.0682	115.0757
647	1-Methylxanthine	C6H6N4O2	166.0492	167.0565
648	Hypoxanthine	C5H4N4O	136.0386	137.0458

649	Sebacic acid	C10H18O4	202.1208	203.128
650	Kynurenic acid	C10H7NO3	189.0428	190.0501
651	Nicotinuric acid	C8H8N2O3	180.0537	181.061
652	N2,N2-Dimethylguanosine	C12H17N5O5	311.123	312.1303
653	L-Phenylalanine	C9H11NO2	165.0791	166.0864
654	Amoxicillin	C16H19N3O5S	365.1045	349.0852
655	α-Aspartylphenylalanine	C13H16N2O5	280.1061	281.1134
656	3-N-Methyl-L-histidine	C7H13N3O3	187.0957	188.103
657	1-Methylguanine	C6H7N5O	165.0652	166.0725
658	Methyl EudesMate	C11H14O5	226.0842	227.0916
659	P-Aminohippuric Acid	C9H10N2O3	194.0694	195.0767
660	7-Hydroxy-3,4-dihydrocarbostyril	C9H9NO2	163.0635	164.0707
661	N-Acetyl-D-tryptophan	C13H14N2O3	246.1005	247.1078
662	Tetranor-PGDM	C16H24O7	310.141	311.1483
663	Indole-3-acetic acid	C10H9NO2	175.0635	176.0708
664	Phenylglyoxylic acid	C8H6O3	150.0319	151.0391
665	isoleucine	C6H13NO2	131.095	132.1021
666	L-Pyroglutamic acid	C5H7NO3	129.0428	130.05
667	2,3-Dinor-TXB2	C18H30O6	342.2038	365.193
668	Acetylcarnitine	C9H17NO4	203.116	226.1052
669	Asp-Phe methyl ester	C14H18N2O5	294.1215	295.1288
670	D-glutamine	C5H10N2O3	146.0692	147.0764
671	L-Leucyl-L-Alanine	C9H18N2O3	202.1318	203.1391
672	7-Methylxanthine	C6H6N4O2	166.0492	167.0565
673	N-Acetyl-L-carnosine	C11H16N4O4	268.117	269.1243
674	2-Methoxyestrone	C19H24O3	300.1726	301.1799
675	Xanthurenic acid	C10H7NO4	205.0377	206.045
676	Imidazolelactic acid	C6H8N2O3	156.0533	157.0608
677	2-Aminoadipic acid	C6H11NO4	161.0688	162.0761
678	alpha-Benzylsuccinic acid	C11H12O4	208.0738	209.0811
679	17α-Ethynylestradiol	C20H24O2	296.178	297.1852
680	Tetrahydrocorticosterone	C21H34O4	367.2725	368.2798
681	5-Hydroxyindole-3-acetic acid	C10H9NO3	191.0583	192.0656
682	N-Formylkynurenine	C11H12N2O4	236.0798	237.0871
683	3-Oxo-7alpha,12alpha-hydroxy-5beta-cholanoic acid	C24H38O5	406.2722	429.2614
684	Homoarginine	C7H16N4O2	188.1274	189.1347
685	Naringenin	C15H12O5	272.0686	273.0758
686	Inosine	C10H12N4O5	268.0809	269.0882
687	Isorhapontigenin	C15H14O4	258.0894	259.0967
688	Uridine	C9H12N2O6	244.0695	245.0768
689	Calcium D-Panthotenate	C18H32CaN2O10	476.1662	477.1735
690	L-Homocitrulline	C7H15N3O3	189.1114	190.1187

691	Methyldopa	C10H13NO4	211.0847	212.0919
692	L-Glutamine	C5H10N2O3	146.0733	147.0806
693	Salicylic acid	C7H6O3	138.0318	139.0391
694	3-Methylindole	C9H9N	131.0737	132.081
695	Corey Lactone Diol	C8H12O4	172.074	173.0812
696	5-Methyl-dl-tryptophan	C12H14N2O2	218.1057	219.113
697	Epinephrine	C9H13NO3	183.0898	201.1236
698	3-Methylhistidine	C7H11N3O2	169.0851	170.0924
699	Guanidinosuccinic acid	C5H9N3O4	175.0594	176.0667
700	Levodopa	C9H11NO4	197.069	198.0762
701	2-(Formylamino)Benzoic Acid	C8H7NO3	165.0427	148.0394
702	D-2-Aminoadipic acid	C6H11NO4	161.0689	144.0656
703	Thymine	C5H6N2O2	126.0432	127.0505
704	3-(3-Methoxyphenyl)propionic acid	C10H12O3	180.078	181.0852
705	L-Tyrosinemethylester	C10H13NO3	195.0897	196.097
706	2,5-Furandicarboxylic acid	C6H4O5	156.0059	139.0026
707	N-Acetyl-L-histidine	C8H11N3O3	197.0802	198.0875
708	L-lysine	C6H14N2O2	146.1055	147.1129
709	beta-Estradiol 17-Acetate	C20H26O3	314.1886	315.1959
710	Ip7G	C16H23N5O5	365.1687	366.176
711	Glycyl-L-leucine	C8H16N2O3	188.1161	227.0792
712	Estrone	C18H22O2	292.14	293.1473
713	Prostaglandin E1	C20H34O5	336.2278	337.2351
714	Isophorone	C9H14O	138.1046	121.1014
715	N-acetyl-L-ornithine	C7H14N2O3	174.1006	197.0898
716	N-METHYL (-)EPHEDRINE	C11H17NO	179.1311	180.1384
717	Prostaglandin J2	C20H30O4	334.2143	335.2215
718	Ala-Gln	C8H15N3O4	217.1065	201.0872
719	Isobutyryl carnitine	C11H21NO4	231.1473	232.1545
720	Estriol	C18H24O3	288.1725	289.1798
721	Prostaglandin A3	C20H28O4	332.1989	333.206
722	Boldione	C19H24O2	284.178	285.1853
723	cis-Aconitic acid	C6H6O6	174.0166	175.0238
724	geranyl pp	C10H20O7P2	314.07	315.0773
725	trans-2-Butene-1,4-dicarboxylic Acid	C6H8O4	144.0424	145.0497
726	(5-L-Glutamyl)-L-Amino Acid	C8H14N2O5	218.0902	219.0974
727	Asp-glu	C9H14N2O7	262.08	263.0873

Table S2 Metabolites identified in negative ion mode.

**No.**	**Metabolites**	**Formula**	**Molecular Weight**	**m/z**

1	Cyclamic acid	C6H13NO3S	179.0583	178.0511
2	3-Indoxyl sulphate	C8H7NO4S	213.0095	212.0022
3	Testosterone sulfate	C19H28O5S	368.1656	367.1584
4	Glutaconic acid	C5H6O4	130.0266	129.0193
5	Saccharin	C7H5NO3S	182.999	181.9917
6	4-Methylphenol	C7H8O	108.0575	107.0503
7	Ascorbic acid	C6H8O6	176.0319	175.0246
8	D-(-)-Quinic acid	C7H12O6	192.0633	191.0561
9	Myricetin	C15H10O8	318.0406	317.0334
10	Benzoic acid	C7H6O2	122.0367	121.0294
11	Gluconic acid	C6H12O7	196.0582	195.0509
12	Purine	C5H4N4	120.0422	119.0349
13	4-Methylcatechol	C7H8O2	124.0524	123.0451
14	L-Threonic acid	C4H8O5	136.0372	135.0299
15	Ibuprofen metabolite B	C13H16O4	236.1048	235.0975
16	2-Hydroxy-1-(4-methoxyphenyl)propyl hexopyranoside	C16H24O8	344.1471	343.1398
17	2-{2-[2,5-di(methoxycarbonyl)anilino]-2-oxoethoxy}acetic acid	C14H15NO8	325.0796	324.0723
18	Chlortetracycline	C22H23ClN2O8	478.1111	477.1038
19	2,4-Dihydroxybenzoic acid	C7H6O4	154.0265	153.0192
20	Theophylline	C7H8N4O2	180.0645	179.0573
21	4-(acetylamino)phenyl 3-chlorobenzoate	C15H12ClNO3	325.0272	324.0199
22	3-Methyladipic acid	C7H12O4	160.0735	159.0662
23	Dimetghyl 4-Hydroxyisophthalate	C10H10O5	210.0529	209.0456
24	3-Hydroxysebacic acid	C10H18O5	218.1154	217.108
25	Dihydroroseoside	C19H32O8	388.2097	387.2025
26	Cholic acid	C24H40O5	408.2877	407.2805
27	D-(-)-Lyxose	C5H10O5	150.0528	149.0455
28	D-(+)-Arabitol	C5H12O5	152.0684	151.0611
29	4-((5-(4-Nitrophenyl)oxazol-2-yl)amino)benzonitrile	C16H10N4O3	306.0772	305.0699
30	Dodecanedioic acid	C12H22O4	230.1517	229.1444
31	Androsterone glucuronide	C25H38O8	466.2568	465.2495
32	D-(-)-Mannitol	C6H14O6	182.079	181.0717
33	Phenylacetaldehyde	C8H8O	120.0574	119.0502
34	3-[(methoxycarbonyl)amino]-2,2,3-trimethylbutanoic acid	C9H17NO4	203.1158	202.1085
35	S-Lactoylglutathione	C13H21N3O8S	379.1091	378.1018
36	Δ17-6-keto prostaglandin F1α	C20H32O6	414.2254	413.2181
37	Citraconic acid	C5H6O4	130.0265	129.0192
38	Glycoursodeoxycholic acid	C26H43NO5	449.3147	448.3074
39	2-Isopropylmalate	C7H12O5	176.0684	175.0611

40	4-Ethylphenol	C8H10O	122.0731	121.0658
41	trans-Aconitic acid	C6H6O6	174.0163	173.009
42	Pseudouridine	C9H12N2O6	244.0694	243.0621
43	3-Hydroxyvaleric acid	C5H10O3	118.063	117.0558
44	β-D-Glucopyranuronic acid	C6H10O7	194.0427	193.0354
45	Capryloylglycine	C10H19NO3	201.1365	200.1292
46	3,8,9-trihydroxy-10-propyl-3,4,5,8,9,10-hexahydro-2H-oxecin-2-one	C12H20O5	244.131	243.1237
47	Ferulic acid	C10H10O4	194.0577	193.0504
48	5,5-Dimethylhydantoin	C5H8N2O2	128.0584	127.0512
49	ethyl 3-[3,5-di(trifluoromethyl)anilino]-2-nitroacrylate	C13H10F6N2O4	372.0514	371.0442
50	N-Isovalerylglycine	C7H13NO3	159.0894	158.0822
51	4-Methyl-2-Oxopentanoic Acid	C6H10O3	130.063	129.0557
52	Glucuronic acid-3,6-lactone	C6H8O6	176.032	175.0247
53	4-Chlorophenol	C6H5ClO	128.0028	126.9955
54	Estriol 17-sulfate	C18H24O6S	368.1293	367.122
55	Sinapinic acid	C11H12O5	224.0683	223.061
56	6,7-Dihydroxycoumarin	C9H6O4	178.0263	177.019
57	Quercetin-3β-D-glucoside	C21H20O12	464.0957	463.0884
58	Gibberellin A7	C19H22O5	330.1468	329.1395
59	L-Malate	C4H6O5	134.0216	133.0142
60	Aflatoxin G2	C17H14O7	330.0773	329.07
61	gamma-Nonanolactone	C9H16O2	156.115	155.1077
62	Genipin	C11H14O5	208.0734	207.0661
63	2-Furoic acid	C5H4O3	112.016	111.0087
64	3-Hydroxybutyric acid	C4H8O3	104.0474	103.0401
65	2-Hydroxyvaleric acid	C5H10O3	118.063	117.0557
66	16α-Hydroxyestrone	C18H22O3	286.1527	285.1454
67	D-(-)-Fructose	C6H12O6	180.0632	179.0559
68	5-chloro-N-(4-morpholinophenyl)-1H-indole-2-carboxamide	C19H18ClN3O2	355.1091	354.1018
69	Phellamurin	C26H30O11	518.1786	517.1713
70	2,2-Bis(hydroxymethyl)propionic acid	C5H10O4	134.0578	133.0506
71	Indole-2-carboxylic acid	C9H7NO2	161.0476	160.0403
72	Ethyl paraben	C9H10O3	166.0629	165.0556
73	3-Hydroxy-3-methylglutaric acid	C6H10O5	162.0527	161.0455
74	Butylparaben	C11H14O3	194.0942	193.0869
75	2-C-methyl D-erythritol 4-phosphate	C5H13O7P	216.0401	215.0328
76	Valproic acid	C8H16O2	144.1151	143.1078
77	Pyrogallol	C6H6O3	126.0316	125.0243
78	Cyclohexaneacetic acid	C8H14O2	142.0994	141.0921

79	N2-Methylguanosine	C11H15N5O5	297.1071	296.0999
80	alpha-D-Glucopyranosyl 2-O-(2-methylbutanoyl)-alpha-D-glucopyranoside	C17H30O12	426.1716	425.1643
81	N-(1-benzylpiperidin-4-yl)-5-methoxy-1H-indole-2-carboxamide	C22H25N3O2	363.1894	362.1821
82	Tyrosol	C8H10O2	138.068	137.0607
83	2-Hydroxy-2-methyl-3-buten-1-yl beta-D-glucopyranoside	C11H20O7	310.1262	309.1189
84	L-(+)-Tartaric acid	C4H6O6	150.0164	149.0091
85	Ciliatine	C2H8NO3P	125.0238	124.0165
86	10-Undecenoic acid	C11H20O2	184.1463	183.139
87	Ethyl-β-D-glucuronide	C8H14O7	222.074	221.0667
88	Prostaglandin E2	C20H32O5	352.225	397.2232
89	Hydrocinnamic acid	C9H10O2	150.0681	149.0607
90	Undecanedioic acid	C11H20O4	216.1361	215.1287
91	9-Methyluric acid	C6H6N4O3	182.0438	181.0366
92	Luteolin	C15H10O6	286.051	285.0438
93	4-Hydroxycoumarin	C9H6O3	162.0316	161.0243
94	Catechol	C6H6O2	110.0366	109.0293
95	N-Isobutyrylglycine	C6H11NO3	145.0739	144.0667
96	4-Hydroxybutyric acid (GHB)	C4H8O3	104.0474	103.0401
97	Ethylmalonate	C5H8O4	132.0421	113.0242
98	7-Hydroxy-4-chromone	C9H6O3	162.0315	161.0242
99	N-(2-morpholinophenyl)-2,1,3-benzoxadiazole-4-sulfonamide	C16H16N4O4S	360.0879	359.0807
100	Suberic acid	C8H14O4	174.0891	173.0818
101	(+/-)12(13)-DiHOME	C18H34O4	296.2349	295.2276
102	Cetirizine N-oxide	C21H25ClN2O4	404.1476	403.1403
103	Quercetin	C15H10O7	302.0458	301.0386
104	Fumaric acid	C4H4O4	116.0109	115.0036
105	Quinic acid	C7H12O6	192.0631	191.0559
106	23-Norcholic acid	C23H38O5	394.2722	393.2649
107	Alanyltyrosine	C12H16N2O4	252.1108	251.1035
108	L-Tryptophan	C11H12N2O2	204.0898	203.0825
109	4-Acetamidobutanoic acid	C6H11NO3	145.0737	144.0664
110	N-Acetyl-L-phenylalanine	C11H13NO3	207.0895	206.0822
111	methadone-d9	C21H18[2]H9NO	317.9551	316.9478
112	3-Hydroxybenzoic acid	C7H6O3	138.0317	137.0244
113	9-(3-O-Methylpentofuranosyl)-1,9-dihydro-6H-purin-6-one	C11H14N4O5	282.0963	281.089
114	Phloretin	C15H14O5	274.084	273.0767
115	N-Acetyl-DL-phenylalanine	C11H13NO3	207.0894	252.0876

116	Methylsuccinic acid	C5H8O4	132.0422	131.0349
117	Orotidine	C10H12N2O8	288.0592	287.052
118	2-Furoylglycine	C7H7NO4	169.0376	168.0303
119	20-Carboxy-Leukotriene B4	C20H30O6	366.2041	365.1969
120	Tartaric acid	C4H6O6	150.0164	149.0091
121	Proline-hydroxyproline	C10H16N2O4	228.111	227.1037
122	4-Hydroxy-3- methoxyphenylglycol sulfate	C9H12O7S	264.0301	263.0229
123	D-Fructose 6-phosphate	C6H13O9P	260.0296	259.0223
124	Cinnamyl alcohol	C9H10O	134.0732	133.066
125	4-Hydroxyisoleucine	C6H13NO3	147.0894	146.0821
126	cis-2-Decenoic acid	C10H18O2	170.1306	169.1233
127	Perillic acid	C10H14O2	166.0993	165.092
128	N'-(benzoyloxy)-2-(2,2-dichlorocyclopropyl)ethanimidamide	C12H12Cl2N2O2	286.0259	285.0186
129	δ-Ribono-1,4-lactone	C5H8O5	148.0371	147.0298
130	nor-6β-Oxycodol	C17H21NO4	303.1504	302.1432
131	L-Hydroxyproline	C5H9NO3	131.0581	130.0508
132	Eugenol	C10H12O2	164.0837	163.0764
133	Asaraldehyde	C10H12O4	196.0734	195.0661
134	Cynaropicrin	C19H22O6	392.1504	391.1431
135	10-Hydroxydecanoic acid	C10H20O3	188.1411	187.1338
136	6-Sialyllactose	C23H39NO19	633.2122	632.2049
137	2,3,6-Trimethylphenol	C9H12O	136.0887	135.0814
138	1,5-Anhydro-D-glucitol	C6H12O5	164.0685	163.0613
139	2-Butoxyacetic acid	C6H12O3	132.0786	131.0713
140	Prostaglandin H1	C20H34O5	336.2301	335.2229
141	16-(Hexopyranosyloxy)-7-hydroxy-8,9-epoxypimaran-18-oic acid	C26H42O10	514.2783	513.2711
142	Syringic acid	C9H10O5	198.0528	197.0455
143	5-(3-chloro-4-methylanilino)-1-methyl-1H-pyrazol-3-ol	C11H12ClN3O	237.0671	236.0598
144	Octanedioic acid	C8H14O4	174.089	173.0817
145	Hexanoic acid	C6H12O2	116.0838	115.0765
146	4-oxo-4,5,6,7-tetrahydrobenzo[b]furan-3-carboxylic acid	C9H8O4	180.0422	179.0349
147	5-amino-1-phenyl-1H-pyrazole-4-carbonitrile	C10H8N4	184.0735	183.0663
148	Glucuronic acid	C6H10O7	194.0425	193.0352
149	Caprylic acid	C8H16O2	144.1148	143.1075
150	N-(2-methyl-5-nitrophenyl)-N-(methylsulfonyl)methanesulfonamide	C9H12N2O6S2	308.0135	307.0062
151	Vanillyl alcohol	C8H10O3	154.0628	153.0555
152	19(R)-Hydroxy-prostaglandin E2	C20H32O6	350.2093	349.202

153	2-Phenylpropionic acid	C9H10O2	150.0678	149.0605
154	Benzyl 6-O-beta-D-glucopyranosyl-beta-D-glucopyranoside	C19H28O11	432.1628	431.1555
155	2-Methylbutyl beta-D-glucopyranoside	C11H22O6	296.1469	295.1396
156	2-(2-carboxy-2-methylpropyl)-4,6-dimethylbenzoic acid	C14H18O4	250.1203	249.113
157	N1-(6-methyl-4-oxo-3,4-dihydroquinazolin-2-yl)-4-nitrobenzamide	C16H12N4O4	324.0862	323.0789
158	4-Oxoproline	C5H7NO3	129.0426	128.0353
159	Prostaglandin H2	C20H32O5	334.2145	333.2072
160	N-Acetylcysteine	C5H9NO3S	163.0302	162.0229
161	L-Glutamic acid	C5H9NO4	147.0532	146.0459
162	tetranor-PGFM	C16H26O7	266.1518	265.1444
163	Pimelic acid	C7H12O4	160.0735	159.0662
164	Valsartan metabolite	C14H10N4O2	266.0802	265.073
165	3-Methylamino-L-alanine	C4H10N2O2	118.0783	117.0711
166	5-Methoxyindole-3-Carbaldehyde	C10H9NO2	175.0632	174.0559
167	5-Sulfosalicylic acid	C7H6O6S	217.9884	216.9812
168	2,3-Dinor-8-epi-prostaglandin F2α	C18H30O5	326.2093	325.202
169	Geniposidic acid	C16H22O10	374.1213	373.114
170	(2S)-4-Oxo-2-phenyl-3,4-dihydro-2H-chromen-7-yl beta-D-glucopyranoside	C21H22O8	448.1351	447.1279
171	Methylmalonic acid	C4H6O4	118.0266	117.0194
172	Ethyl chrysanthemumate	C12H20O2	196.1463	195.1389
173	3-(2-Hydroxyethyl)indole	C10H11NO	161.084	160.0768
174	3-Methylglutaric acid	C6H10O4	146.0581	145.0509
175	Heptanoic acid	C7H14O2	130.0994	111.0814
176	ST 24:1;O5;S	C24H40O8S	488.2446	487.2373
177	N-Acetylglycine	C4H7NO3	117.0426	116.0353
178	Genistein	C15H10O5	270.0524	269.0451
179	Nonanoic acid	C9H18O2	158.1306	157.1233
180	2-Methylpentanedioic acid	C6H10O4	146.0576	191.0556
181	L-Aspartic acid	C4H7NO4	133.0374	132.0301
182	3-Indoleacrylic acid	C11H9NO2	187.0631	186.0558
183	5-Aminovaleric acid	C5H11NO2	117.079	116.0717
184	D-Pantethine	C22H42N4O8S2	554.2364	553.2292
185	Xanthohumol	C21H22O5	354.1425	353.1352
186	8-iso-15-keto Prostaglandin F2α	C20H32O5	352.225	351.2177
187	19(R)-hydroxy Prostaglandin E2	C20H32O6	350.2093	349.2022
188	Mesaconic acid	C5H6O4	130.0267	129.0194
189	N-Acetyl-D-galactosamine 4-sulfate	C8H15NO9S	301.0465	300.0392
190	Sucrose	C12H22O11	342.1102	341.1029

191	Deoxyribose 5-Phosphate	C5H11O7P	214.0244	213.0171
192	Prostaglandin F3α	C20H32O5	398.2306	397.2234
193	Glycerol 3-phosphate	C3H9O6P	172.0137	171.0064
194	(±)9-HpODE	C18H32O4	312.2303	311.223
195	methyl 2-[(2-acetyl-3-oxo-1-butenyl)amino]acetate	C9H13NO4	199.0843	198.077
196	2-Isopropylmalic acid	C7H12O5	176.0684	175.0611
197	Oxaceprol	C7H11NO4	173.0687	172.0615
198	Glyphosate	C3H8NO5P	169.0138	168.0065
199	L-Ascorbic acid 2-sulfate	C6H8O9S	255.9888	254.9815
200	6-Keto-prostaglandin f1alpha	C20H34O6	370.2353	369.228
201	Biocytin	C16H28N4O4S	372.1761	371.1689
202	JNJ-1661010	C19H19N5OS	365.1322	364.1249
203	3-Coumaric acid	C9H8O3	164.0474	163.0401
204	1,3-Dimethyluracil	C6H8N2O2	140.0585	139.0513
205	5,7-Dihydroxy-2-(3-hydroxy-4-methoxyphenyl)chroman-4-one	C16H14O6	302.0789	301.0716
206	17(S)-HpDHA	C22H32O4	396.1994	395.1922
207	Adipic acid	C6H10O4	146.0579	145.0506
208	Tretinoin	C20H28O2	300.2051	299.1978
209	5-[(Benzoyloxy)methyl]-4,5,6-trihydroxy-2-cyclohexen-1-yl benzoate	C21H20O7	384.1267	383.1194
210	Sulfoacetic acid	C2H4O5S	139.9779	138.9707
211	3,4-Dihydroxyphenylpropionic acid	C9H10O4	182.0579	181.0506
212	Capric acid	C10H20O2	172.1463	171.1391
213	D-Threose	C4H8O4	120.0421	119.0348
214	ST 24:1;O4;T	C26H45NO7S	515.2927	514.2854
215	5-Aminopentanoate	C5H11NO2	117.079	116.0717
216	15(R)-Prostaglandin D2	C20H32O5	352.225	351.2178
217	N1-(4-chlorophenyl)-2-cyano-4,4-dimethyl-3-oxopentanamide	C14H15ClN2O2	278.0808	277.0735
218	16-Glucuronide-estriol	C24H32O9	464.2055	463.1982
219	13,14-dihydro-19(R)-hydroxy Prostaglandin E1	C20H36O6	372.2511	371.2439
220	D-Saccharic acid	C6H10O8	210.0375	209.0303
221	NSI-189	C22H30N4O	366.2406	365.2333
222	Nicotinamide N-oxide	C6H6N2O2	138.043	137.0357
223	DL-Malic acid	C4H6O5	134.0214	133.0142
224	(+/-)9,10-dihydroxy-12Z-octadecenoic acid	C18H34O4	314.2459	313.2387
225	3'-Adenosine monophosphate (3'-AMP)	C10H14N5O7P	347.0613	346.0541
226	β-Estradiol-17β-glucuronide	C24H32O8	448.2099	447.2026
227	13,14-dihydro Prostaglandin F1α	C20H38O5	394.2469	393.2397
228	Allantoin	C4H6N4O3	158.0439	157.0366
229	N1-(4-chlorophenyl)-3-(1H-pyrrol-1-	C17H20ClN3O	317.1293	316.122

	ylmethyl)piperidine-1-carboxamide			
230	2-({[4-(6-methyl-1,3-benzothiazol-2-yl)phenyl]imino}methyl)phenol	C21H16N2OS	344.0928	343.0854
231	L-Cystine	C6H12N2O4S2	240.0238	239.0165
232	Cystathionine	C7H14N2O4S	222.0675	221.0602
233	2-Methylbutyroylcarnitine	C12H23NO4	245.1627	244.1554
234	2'-O-Methyluridine	C10H14N2O6	258.0851	257.0779
235	Protocatechuic acid	C7H6O4	154.0265	153.0192
236	Levulinic acid	C5H8O3	116.0475	115.0402
237	S-Sulfo-L-cysteine	C3H7NO5S2	200.9766	199.9693
238	2-Aminobenzenesulfonic acid	C6H7NO3S	173.0146	172.0073
239	N-{5-[(dimethylamino)sulfonyl]-2-methylphenyl}cyclohexanecarboxamide	C16H24N2O3S	324.1532	323.1459
240	4-hydroxy-1-methyl-3-(phenylthio)-1,2-dihydroquinolin-2-one	C16H13NO2S	283.0653	282.0581
241	Valeric acid	C5H10O2	102.0682	101.0609
242	4-(octyloxy)benzoic acid	C15H22O3	250.1568	249.1495
243	Robenidine	C15H13Cl2N5	333.0517	332.0444
244	Dl-Lanthionine	C6H12N2O4S	208.0518	207.0446
245	N-Acetyl-Asp-Glu	C11H16N2O8	304.0905	303.0832
246	Hexadecanedioic acid	C16H30O4	286.2144	285.2071
247	D-Glucuronic acid	C6H10O7	194.0423	193.035
248	Orotic Acid	C5H4N2O4	156.0173	155.01
249	Flavin mononucleotide (FMN)	C17H21N4O9P	456.1034	455.0961
250	13(S)-HOTrE	C18H30O3	294.2195	293.2123
251	Monobutyl phthalate	C12H14O4	222.0891	221.0819
252	Quinoline-4-carboxylic acid	C10H7NO2	173.0475	172.0402
253	3-amino-2,6-diphenyl-4,7-dihydro-2H-pyrazolo[3,4-d]pyrimidin-4-one	C17H13N5O	303.1106	302.1033
254	Acetildenafil	C25H34N6O3	466.2779	465.2706
255	1-Pyrenol	C16H10O	218.0731	217.0658
256	Lactobionic acid	C12H22O12	358.111	357.1037
257	Sepiapterin	C9H11N5O3	237.0848	236.0775
258	4-Hydroxyretinoic Acid	C20H28O3	316.2039	315.1966
259	6α-Prostaglandin I1	C20H34O5	354.2408	353.2335
260	N6-Succinyl Adenosine	C14H17N5O8	383.1073	382.1001
261	Dl-2-Amino-3-phosphonopropionic acid	C3H8NO5P	169.0134	168.0062
262	L-(-)-Glyceric acid	C3H6O4	106.0265	105.0192
263	L-Methionine sulfone	C5H11NO4S	181.0409	180.0336
264	Dithranol	C14H10O3	226.062	225.0547
265	2-[6-(1H-benzo[d]imidazol-2-yl)-2-pyridyl]-1H-benzo[d]imidazole	C19H13N5	312.1211	311.1138

266	Homotaurine	C3H9NO3S	139.0304	138.0232
267	L-Anserine	C10H16N4O3	240.1221	239.1149
268	NADH	C21H29N7O14P2	665.1236	664.1163
269	Phenylacetylglutamine	C13H16N2O4	264.1111	263.1039
270	Sodium Dehydrocholate	C24H33NaO5	424.2212	423.2139
271	1-{[5-(4-chlorophenyl)-4H-1,2,4-triazol-3-yl]thio}acetone	C11H10ClN3OS	534.0497	266.0176
272	Jasmonic acid	C12H18O3	210.1254	209.1181
273	Asparagine	C4H8N2O3	132.0534	131.0462
274	Estrone sulfate	C18H22O5S	350.1211	349.1138
275	Lauric acid	C12H24O2	200.1775	199.1702
276	4-Amino-5-imidazolecarboxamide	C4H6N4O	126.0543	125.047
277	Phenobarbital	C12H12N2O3	232.0888	231.0815
278	Oxoadipic Acid	C6H8O5	160.037	159.0298
279	Dl-Threitol	C4H10O4	122.0578	121.0506
280	13-Hpotre(R)	C18H30O4	310.2145	309.2072
281	ethyl 3-cyano-2-hydroxy-6-phenylisonicotinate	C15H12N2O3	268.0807	267.0734
282	[1,1'-biphenyl]-2,2'-dicarboxylic acid	C14H10O4	242.058	241.0507
283	8,15-Dihete	C20H32O4	336.2298	335.2225
284	2-Anisic acid	C8H8O3	152.0473	151.04
285	L-Histidine	C6H9N3O2	155.0696	154.0623
286	Uric acid	C5H4N4O3	168.0282	167.0209
287	Xanthine	C5H4N4O2	152.0333	151.026
288	Paracetamol	C8H9NO2	151.0632	150.0559
289	Asp-Phe	C13H16N2O5	280.1058	279.0985
290	Mevalonic acid	C6H12O4	148.0735	147.0662
291	1,3-Dimethyluric acid	C7H8N4O3	196.0593	195.052
292	D-(-)-Glutamine	C5H10N2O3	146.069	145.0617
293	Porphobilinogen	C10H14N2O4	226.0953	225.0881
294	N-Acetyl-L-leucine	C8H15NO3	173.1052	172.0979
295	Kynurenic acid O-hexside	C16H17NO8	351.0952	350.088
296	Citric acid	C6H8O7	192.0269	191.0196
297	Gluconolactone	C6H10O6	178.0477	177.0404
298	Traumatic acid	C12H20O4	228.1362	227.1289
299	Xanthosine	C10H12N4O6	284.0756	283.0683
300	Cinnamoylglycine	C11H11NO3	205.0738	204.0665
301	Acetaminophen glucuronide	C14H17NO8	327.0953	326.088
302	1,3,7-Trimethyluric acid	C8H10N4O3	210.0753	209.068
303	DL-m-Tyrosine	C9H11NO3	181.0738	180.0665
304	N4-Acetylcytidine	C11H15N3O6	285.096	284.0887
305	Daidzein	C15H10O4	254.0579	253.0506
306	Chenodeoxycholic acid-3-beta-D-glucuronide	C30H48O10	568.325	567.3178

307	Glycocholic acid	C26H43NO6	465.3091	464.3018
308	L-Ascorbate	C6H8O6	176.0319	175.0246
309	Pyridoxine O-Glucoside	C14H21NO8	331.1267	330.1194
310	2-Hydroxyhippuric acid	C9H9NO4	195.053	194.0458
311	Boc-beta-cyano-L-alanine	C9H14N2O4	214.0952	213.088
312	Homogentisic Acid	C8H8O4	168.0421	167.0349
313	Epitestosterone glucuronide	C25H36O8	464.2413	463.234
314	Chlorogenic Acid Methyl Ester	C17H20O9	368.1108	367.1035
315	Lipoic acid	C8H14O2S2	206.0426	205.0353
316	Royal jelly acid	C10H18O3	186.1255	185.1183
317	Tetradecanedioic acid	C14H26O4	258.1832	257.1759
318	Caffeic acid	C9H8O4	180.0422	179.0349
319	4-Pyridoxic acid	C8H9NO4	183.0531	182.0458
320	Hippuric acid	C9H9NO3	179.0583	178.051
321	Taurine	C2H7NO3S	125.0146	124.0073
322	Taurochenodeoxycholic acid	C26H45NO6S	499.2979	498.2907
323	Cystine	C6H12N2O4S2	240.0239	239.0166
324	Prostaglandin G2	C20H32O6	368.2194	367.2119
325	DL-α-Aminocaprylic acid	C8H17NO2	159.1259	158.1186
326	5-Hydroxyindole	C8H7NO	133.0522	132.0449
327	Delta-Tridecalactone	C13H24O2	212.1777	211.1705
328	5-Methyluridine	C10H14N2O6	258.0852	257.0779
329	P-Coumaroyl Agmatine	C14H20N4O2	276.1573	275.15
330	Gamma-Glu-Leu	C11H20N2O5	260.1372	259.1299
331	4-Hydroxybenzaldehyde	C7H6O2	122.0368	121.0295
332	Pantothenic acid	C9H17NO5	219.1105	218.1033
333	Adenosine 3'5'-cyclic monophosphate	C10H12N5O6P	329.0524	328.0452
334	1-Caffeoylquinic Acid	C16H18O9	354.095	353.0876
335	Prostaglandin K2	C20H30O5	350.2093	349.2021
336	N-Acetylneuraminic acid	C11H19NO9	309.1058	308.0985
337	Dl-3,4-Dihydroxymandelic Acid	C8H8O5	184.0371	183.0298
338	L-cysteine	C3H7NO2S	121.02	166.0183
339	Azelaic acid	C9H16O4	188.1048	187.0975
340	Carnosine	C9H14N4O3	226.1066	225.0993
341	N-Carbamyl-L-glutamicacid	C6H10N2O5	190.0589	171.0411
342	aminoimidazole carboxamide ribonucleotide	C9H15N4O8P	338.0636	337.0564
343	5-Hydroxytryptophan	C11H12N2O3	220.0846	219.0774
344	1,7-Dimethyluric acid	C7H8N4O3	196.0595	195.0522
345	5-Hydroxydiclofenac	C14H11Cl2NO3	311.0116	310.0043
346	N-[4-(diethylamino)phenyl]-N'-phenylurea	C17H21N3O	319.1451	318.1378
347	N-Acetyl-L-tyrosine	C11H13NO4	223.0842	222.077
348	10-Nitrolinoleate	C18H31NO4	325.2253	324.2181

349	Propionyl-L-carnitine	C10H19NO4	217.1313	216.124
350	Homovanillic acid	C9H10O4	182.0579	181.0507
351	Uracil	C4H4N2O2	112.0272	111.02
352	alpha-Ketoglutaric acid	C5H6O5	146.0214	145.0141
353	N-Acetyl-Dl-glutamic acid	C7H11NO5	189.0637	188.0565
354	N-lactoyl-phenylalanine	C12H15NO4	237.1	236.0928
355	SDMA	C8H18N4O2	202.1429	201.1356
356	Homocysteic acid	C4H9NO5S	183.0201	182.0128
357	Dl-3-Hydroxynorvaline	C5H11NO3	133.0739	132.0666
358	N-Acetyl-1-aspartylglutamic acid	C11H16N2O8	304.0905	303.0832
359	Acetylcysteine	C5H9NO3S	163.0303	162.023
360	Vitamin B2	C17H20N4O6	376.1374	375.1302
361	N-Acetyl-aspartic acid	C6H9NO5	175.0479	174.0406
362	1-Methyluric acid	C6H6N4O3	182.0441	181.0369

Table S3 Differential metabolites in HUA vs control.

Metabolite	FC	log2FC	P	VIP	Up.Down
Cotinine	1.71	0.77	5.24E-07	1.10	up
gamma-Glutamylleucine	0.59	-0.77	1.22E-05	2.99	down
Gamma-Glu-Leu	0.46	-1.12	2.87E-05	2.90	down
Isovanillic acid	1.65	0.72	4.80E-04	2.34	up
Asp-Phe methyl ester	0.52	-0.94	4.85E-04	2.93	down
L-Kynurenine	0.49	-1.03	7.53E-04	2.35	down
16-(Hexopyranosyloxy)-7-hydroxy-8,9-epoxypimaran-18-oic acid	0.35	-1.52	9.81E-04	2.04	down
Leukotriene E4	0.26	-1.94	1.02E-03	2.15	down
Apocynin	1.87	0.90	1.41E-03	2.35	up
Propionylcarnitine	0.31	-1.67	1.55E-03	1.84	down
β-D-Glucopyranuronic acid	1.54	0.63	1.61E-03	1.90	up
L-Glutamate	1.63	0.71	1.65E-03	1.75	up
Tyrosylalanine	0.57	-0.80	1.72E-03	2.19	down
Sinapinic acid	3.05	1.61	1.81E-03	1.89	up
11-Oxoetiocholanolone	0.58	-0.78	2.11E-03	1.80	down
Isoferulic acid	1.80	0.85	2.17E-03	2.21	up
Scopoletin	4.46	2.16	3.80E-03	2.43	up
2-(2-acetyl-3,5-dihydroxyphenyl)acetic acid	2.53	1.34	3.82E-03	2.27	up
1-Methylnicotinamide	1.80	0.85	5.06E-03	2.12	up
α-Hydroxyhippuric acid	1.90	0.92	5.46E-03	1.50	up
Pantothenic acid	0.61	-0.72	5.78E-03	2.08	down
N-(5-Aminopentyl)acetamide	2.03	1.02	6.54E-03	2.56	up
ethyl 4-hydroxy-2-[(4-methoxyphenoxy)methyl]pyrimidine-5-carboxylate	2.69	1.43	6.59E-03	2.29	up

L-Homocitrulline	1.61	0.69	6.87E-03	1.92	up
6α-Prostaglandin I1	0.57	-0.82	7.26E-03	1.64	down
Acetyl-L-carnitine	0.29	-1.77	7.31E-03	1.65	down
3-amino-2,6-diphenyl-4,7-dihydro-2H-pyrazolo[3,4-d]pyrimidin-4-one	0.33	-1.59	7.78E-03	2.40	down
Lactobionic acid	1.51	0.59	7.85E-03	1.63	up
L-Carnitine	0.64	-0.64	7.88E-03	1.04	down
D-Saccharic acid	1.78	0.83	8.76E-03	1.53	up
Tretinoin	0.59	-0.76	9.40E-03	1.61	down
2-{2-[(1-methyl-1H-pyrazol-5-yl)amino]-2-oxoethoxy}acetic acid	0.40	-1.32	9.43E-03	1.45	down
L(-)-Carnitine	0.55	-0.87	1.10E-02	1.25	down
alpha-Benzylsuccinic acid	2.31	1.21	1.34E-02	1.57	up
(+/-)-Equol	0.23	-2.10	1.42E-02	1.76	down
L-Tryptophan	0.63	-0.66	1.45E-02	1.70	down
DL-Carnitine	0.46	-1.11	1.52E-02	1.16	down
Dimetghyl 4-Hydroxyisophthalate	9.74	3.28	1.59E-02	2.92	up
Salicylic acid	1.63	0.70	1.69E-02	1.73	up
aminoimidazole carboxamide ribonucleotide	2.24	1.17	1.81E-02	1.64	up
3-(2-Hydroxyethyl)indole	0.40	-1.32	1.85E-02	1.72	down
Obscurolide A1	1.58	0.66	1.86E-02	1.43	up
Biocytin	0.58	-0.79	1.89E-02	1.59	down
3-Methylhistidine	1.94	0.95	1.96E-02	1.74	up
3-benzyl-1-butyl-4-hydroxy-1,2-dihydroquinolin-2-one	1.90	0.92	1.98E-02	1.67	up
Caffeic acid	3.25	1.70	2.08E-02	2.04	up
Sodium cholate	4.61	2.20	2.31E-02	2.80	up
N-Acetyl-S-allyl-L-cysteine	0.30	-1.75	2.31E-02	1.40	down
1-Methyluric acid	1.76	0.82	2.43E-02	1.47	up
Phenylglyoxylic acid	1.66	0.73	2.44E-02	2.09	up
7-methyl-3-nitroimidazo[1,2-a]pyridine	0.60	-0.74	2.60E-02	1.81	down
ST 24:1;O4;T	0.35	-1.51	2.61E-02	1.48	down
4-Hydroxyisoleucine	0.52	-0.94	2.66E-02	1.17	down
5-[3-(2-Chloro-4-fluorobenzyl)-1,2,4-oxadiazol-5-yl]-3-pyrrolidinol	0.48	-1.06	2.78E-02	1.68	down
Ferulic acid	2.78	1.48	2.90E-02	1.97	up
N-Formylkynurenine	0.43	-1.23	2.90E-02	1.36	down
Nonanoic acid	0.66	-0.60	2.97E-02	1.34	down
Thymine	2.48	1.31	3.35E-02	2.10	up
Pyridoxal 5'-Phosphate	0.49	-1.04	3.76E-02	1.40	down
Dl-2-Amino-3-phosphonopropionic acid	1.90	0.92	3.81E-02	1.45	up
N-P-Coumaroyl Spermidine	0.62	-0.69	3.98E-02	1.02	down
Phenylethanolamine	0.49	-1.04	4.01E-02	1.49	down
1-(7-methoxy-2-oxo-2H-chromen-8-yl)-3-methyl-2-oxobutyl acetate	0.42	-1.25	4.02E-02	1.54	down

3',5,7-Trihydroxy-4'-methoxyflavanone	4.08	2.03	4.05E-02	1.94	up
Acetylcysteine	0.60	-0.74	4.06E-02	1.11	down
2-[(3S)-1-(Benzylsulfonyl)-3-pyrrolidinyl]-1-methyl-1H-benzimidazole	3.54	1.83	4.33E-02	1.74	up
N,5-Bis(3-nitrophenyl)oxazol-2-amine	2.51	1.33	4.58E-02	1.75	up
Ciliatine	3.82	1.93	4.64E-02	1.72	up
Pantethine	0.59	-0.76	4.81E-02	1.33	down
3-(tert-butyl)-1-methyl-N-(2-oxo-3-azepanyl)-1H-pyrazole-5-carboxamide	0.33	-1.59	4.96E-02	1.37	down

Table S4 Differential metabolites in AGA vs control.

Metabolite	FC	log2FC	Pvalue	VIP	Up.Down
Cotinine	10.24	3.36	1.80E-09	2.99	up
Oxoamide	16.53	4.05	9.63E-09	2.74	up
trans-3-Hydroxycotinine	34.76	5.12	1.33E-08	2.72	up
Cotinine N-oxide	6.21	2.63	2.41E-08	2.63	up
Palmitic Acid	10.87	3.44	4.45E-08	2.58	up
Levulinic acid	1.60	0.68	1.15E-07	2.68	up
N1-[2-oxo-6-(1H-pyrrol-1-yl)-2H-chromen-3-yl]acetamide	17.68	4.14	2.04E-07	2.50	up
3-Methylindole	5.29	2.40	2.39E-07	2.61	up
Oleamide	2.67	1.41	8.01E-07	2.06	up
Nicotine	12.77	3.67	1.05E-06	2.72	up
3-Methyladipic acid	2.12	1.09	1.59E-06	2.15	up
2,4-Dihydroxybenzoic acid	2.81	1.49	1.72E-06	2.16	up
Xanthohumol	7.52	2.91	4.01E-06	2.39	up
2-Oxindole	0.47	-1.08	6.85E-06	1.97	down
1-butyl-2-methyl-4-nitro-1H-imidazole	0.52	-0.96	1.06E-05	2.14	down
Adipic acid	1.51	0.59	1.42E-05	2.22	up
Arachidonoyl amide	2.73	1.45	1.59E-05	2.03	up
2-Amino-1,3,4-octadecanetriol	6.44	2.69	1.73E-05	1.87	up
1-{4-methoxy-3-[(2-pyridylthio)methyl]phenyl}ethan-1-one	1.59	0.67	1.80E-05	2.14	up
2-imino-8-methoxy-2H-chromene-3-carbonitrile	7.06	2.82	2.01E-05	2.07	up
Salicylic acid	1.75	0.81	2.35E-05	2.02	up
Pimelic acid	1.50	0.59	2.51E-05	2.25	up
Hexanoic acid	2.00	1.00	2.62E-05	1.96	up
4-oxo-4,5,6,7-tetrahydrobenzo[b]furan-3-carboxylic acid	3.01	1.59	3.60E-05	1.88	up
1-(4-methyl-2-morpholino-1,3-thiazol-5-yl)ethan-1-one	2.69	1.43	3.94E-05	1.86	up
Nicotinate ribonucleoside	2.12	1.08	4.30E-05	1.79	up
2-[6-(1H-benzo[d]imidazol-2-yl)-2-pyridyl]-1H-benzo[d]imidazole	4.20	2.07	5.23E-05	2.12	up

1-(6-methyl-3-pyridyl)ethan-1-one O1-ethyloxime hydrochloride	3.73	1.90	5.80E-05	1.98	up
2-(4-chlorophenyl)-2-oxoethyl 2,6-bis(trifluoromethyl)benzoate	29.99	4.91	5.97E-05	2.47	up
Glycoursodeoxycholic acid	0.29	-1.77	1.01E-04	1.81	down
Leukotriene E4	0.30	-1.72	1.08E-04	2.14	down
3,3-dimethyl-2-morpholino-2,3-dihydrobenzo[b]furan-5-ol	0.47	-1.09	1.08E-04	1.96	down
2-Isopropylmalic acid	1.55	0.63	1.14E-04	1.95	up
Xanthosine	3.02	1.60	1.18E-04	2.04	up
10-Hydroxydecanoic acid	2.30	1.20	1.31E-04	1.85	up
N-Acetyl-D-tryptophan	0.60	-0.73	1.31E-04	2.01	down
Δ2-trans-Hexadecenoic acid	1.53	0.61	1.33E-04	1.16	up
2,6-Dihydroxypurine	1.99	0.99	1.42E-04	2.06	up
Phenylacetylglutamine	0.66	-0.60	1.79E-04	1.86	down
6-Sialyllactose	1.85	0.89	2.44E-04	1.89	up
2-phenyl[1,3]oxazolo[4,5-c]quinolin-4(5H)-one	0.38	-1.41	2.77E-04	1.86	down
Benzyl 6-O-beta-D-glucopyranosyl-beta-D-glucopyranoside	2.04	1.03	2.88E-04	1.78	up
2-Thio-acetyl MAGE	0.35	-1.51	3.39E-04	1.86	down
1-hydroxy-1-(4-methoxyphenyl)propan-2-yl 4-methoxybenzoate	0.43	-1.23	3.44E-04	1.88	down
Heptanoic acid	2.06	1.04	3.73E-04	1.72	up
L-Homocitrulline	0.61	-0.72	3.84E-04	1.67	down
N-Phenylacetylglutamine	0.65	-0.63	3.92E-04	1.97	down
Valproic acid	2.21	1.14	4.43E-04	1.73	up
4-Hydroxybenzaldehyde	1.69	0.76	4.79E-04	1.81	up
YNH	2.96	1.57	4.89E-04	1.73	up
3-Methoxytyramine	3.16	1.66	5.51E-04	1.89	up
16-(Hexopyranosyloxy)-7-hydroxy-8,9-epoxypimaran-18-oic acid	0.45	-1.15	5.86E-04	1.72	down
3-morpholino-5,6-diphenylpyridazine-4-carbonitrile	0.53	-0.92	5.97E-04	1.87	down
Dl-3,4-Dihydroxymandelic Acid	4.02	2.01	6.21E-04	1.56	up
2-Isopropylaniline	1.62	0.70	6.59E-04	1.72	up
Phenylacetaldehyde	2.82	1.50	6.77E-04	1.86	up
Cystathionine	2.98	1.58	8.09E-04	1.86	up
Ecgonine methyl ester	1.51	0.60	8.72E-04	1.85	up
5-amino-1-phenyl-1H-pyrazole-4-carbonitrile	1.55	0.63	8.75E-04	1.66	up
DL-m-Tyrosine	1.82	0.86	9.01E-04	1.79	up
Phe-Pro	2.26	1.17	9.45E-04	1.66	up
Senecionine	0.59	-0.76	1.01E-03	1.63	down
Kynurenic acid O-hexside	1.73	0.79	1.05E-03	1.55	up
Diphenylamine	1.57	0.65	1.07E-03	1.27	up
5-ethoxy-2-[(2,3,4,5,6-pentafluorobenzyl)thio]-1H-	94.25	6.56	1.10E-03	2.01	up

benzo[d]imidazole					
4-(tert-butyl)phenyl 3,5-dimethylisoxazole-4-carboxylate	0.60	-0.73	1.12E-03	1.63	down
(2E)-3-phenyl-N-(2-phenylethyl)prop-2-enamide	0.65	-0.61	1.25E-03	1.79	down
Terephthalic Acid	2.39	1.25	1.28E-03	1.55	up
4-Methylphenol	0.51	-0.96	1.31E-03	1.62	down
N-(2-Furoyl)glycine	2.04	1.03	1.47E-03	1.62	up
Lactobionic acid	1.82	0.86	1.47E-03	1.72	up
4-methyl-5-oxo-2-pentyl-2,5-dihydrofuran-3-carboxylic acid	2.16	1.11	1.50E-03	1.56	up
Febuxostat	311.60	8.28	1.63E-03	1.72	up
N-Acetyl-DL-phenylalanine	1.69	0.76	1.76E-03	1.56	up
Tramadol N-Oxide	0.54	-0.89	1.79E-03	1.57	down
Boc-beta-cyano-L-alanine	1.70	0.77	1.88E-03	1.64	up
2-(benzylthio)-4-[2-(3-methylbenzo[b]thiophen-2-yl)vinyl]pyrimidine	19.63	4.29	1.89E-03	1.96	up
Acetylcholine	1.68	0.75	1.90E-03	1.04	up
5-chloro-N-(4-morpholinophenyl)-1H-indole-2-carboxamide	0.66	-0.60	1.99E-03	1.44	down
Acetildenafil	4.58	2.20	2.00E-03	1.86	up
Ethyl-β-D-glucuronide	7.50	2.91	2.01E-03	1.79	up
methyl 2-[(2-acetyl-3-oxo-1-butenyl)amino]acetate	1.88	0.91	2.35E-03	1.53	up
3-Indoleacrylic acid	1.60	0.67	2.38E-03	1.61	up
11-Oxoetiocholanolone	0.66	-0.61	2.53E-03	1.70	down
Methionine sulfoxide	1.66	0.73	2.65E-03	1.63	up
Xanthine	3.60	1.85	2.67E-03	1.52	up
2-Phenylpropionic acid	1.72	0.78	2.69E-03	1.63	up
Isorhapontigenin	0.58	-0.80	2.73E-03	1.44	down
Pyridoxine O-Glucoside	2.05	1.04	2.82E-03	1.52	up
Sucrose	4.18	2.06	2.84E-03	1.61	up
3-[(methoxycarbonyl)amino]-2,2,3-trimethylbutanoic acid	1.63	0.70	2.97E-03	1.53	up
GPH	0.45	-1.14	3.14E-03	1.61	down
2,2-dimethyl-6,7-di[(4-nitrobenzyl)oxy]chroman-4-one	0.52	-0.95	3.21E-03	1.46	down
Caprylic acid	2.01	1.01	3.30E-03	1.69	up
2-Furoylglycine	4.00	2.00	3.33E-03	1.61	up
N-(2-hydroxy-2-phenylethyl)-N'-(2-thienyl)urea	1.89	0.92	3.38E-03	1.60	up
AKB48 N-(5-hydroxypentyl) metabolite	1.98	0.98	3.77E-03	1.37	up
2-[(3S)-1-(Benzylsulfonyl)-3-pyrrolidinyl]-1-methyl-1H-benzimidazole	2.75	1.46	3.94E-03	1.45	up
1-Caffeoylquinic Acid	2.13	1.09	4.02E-03	1.52	up
7-Methylxanthine	2.08	1.06	4.57E-03	1.50	up
Norfloxacin	15.09	3.92	4.74E-03	1.59	up
4-Methoxycinnamic acid	2.39	1.26	4.80E-03	1.58	up

Orotidine	1.92	0.94	5.08E-03	1.53	up
Histamine	0.64	-0.65	5.39E-03	1.16	down
3-(2-Hydroxyethyl)indole	0.40	-1.31	5.55E-03	1.40	down
4-(pentyloxy)benzene-1-carbohydrazide	1.90	0.93	6.20E-03	1.34	up
(+/-)-Equol	0.41	-1.28	6.29E-03	1.54	down
Scopoletin	2.38	1.25	6.65E-03	1.40	up
3-(5-phenyl-1,3-oxazol-2-yl)-4-(trifluoromethyl)pyridine	2.51	1.33	7.99E-03	1.41	up
Caffeine	2.74	1.46	8.12E-03	1.43	up
1-(7-methoxy-2-oxo-2H-chromen-8-yl)-3-methyl-2-oxobutyl acetate	0.43	-1.23	8.22E-03	1.47	down
Ferulic acid	1.78	0.83	8.36E-03	1.40	up
5-Sulfosalicylic acid	0.56	-0.85	8.81E-03	1.38	down
morpholino(quinolin-6-yl)methanone	1.55	0.63	9.07E-03	1.34	up
1,3,7-Trimethyluric acid	3.15	1.65	9.10E-03	1.41	up
2-phenyl-2,4,6,7-tetrahydrothiino[4,3-c]pyrazol-3-ol	4.92	2.30	9.73E-03	1.23	up
ethyl 3-cyano-2-hydroxy-6-phenylisonicotinate	4.84	2.27	9.84E-03	1.49	up
Allantoin	1.66	0.73	9.97E-03	1.35	up
1-Methylxanthine	2.23	1.15	1.04E-02	1.35	up
ST 24:1;O4;T	0.36	-1.48	1.04E-02	1.31	down
2-oxopiperidine-3-carbohydrazide	0.66	-0.61	1.06E-02	1.16	down
L-Ascorbate	1.65	0.72	1.07E-02	1.36	up
16-Glucuronide-estriol	1.78	0.83	1.09E-02	1.29	up
L-Glutamate	1.75	0.80	1.12E-02	1.37	up
Melatonin	2.06	1.04	1.16E-02	1.32	up
Phenylethanolamine	0.46	-1.13	1.18E-02	1.38	down
ILK	2.01	1.01	1.18E-02	1.17	up
Nicotinamide	1.85	0.89	1.23E-02	1.41	up
Tyramine	2.82	1.50	1.30E-02	1.24	up
Taurine	1.79	0.84	1.33E-02	1.47	up
2-Methylpentanedioic acid	2.09	1.06	1.35E-02	1.39	up
β-Estradiol-17β-glucuronide	2.37	1.25	1.37E-02	1.46	up
N,5-Bis(3-nitrophenyl)oxazol-2-amine	1.72	0.78	1.37E-02	1.07	up
3-Methylglutaric acid	1.56	0.64	1.38E-02	1.39	up
5-Methoxyindole-3-Carbaldehyde	2.91	1.54	1.40E-02	1.50	up
4-Hydroxyquinoline	0.48	-1.07	1.47E-02	1.33	down
D-(-)-Quinic acid	1.95	0.96	1.51E-02	1.36	up
Undecanedioic acid	1.79	0.84	1.56E-02	1.38	up
L-Cystine	3.25	1.70	1.60E-02	1.35	up
4-(cyclohexylmethyl)-6-(2-thienyl)-2,3-dihydropyridazin-3-one hydrate	0.55	-0.88	1.83E-02	1.21	down
methyl 3,4,5-trihydroxycyclohex-1-ene-1-carboxylate	3.58	1.84	1.84E-02	1.08	up
N-Acetyl-L-methionine	1.51	0.60	1.87E-02	1.14	up
2-Methylbutyl beta-D-glucopyranoside	1.93	0.95	1.90E-02	1.07	up

2-Isopropylmalate	2.89	1.53	1.97E-02	1.45	up
RPH	0.61	-0.71	2.00E-02	1.23	down
1,7-Dimethyluric acid	2.97	1.57	2.07E-02	1.22	up
(1R,2R)-trans-N-Boc-1,2-cyclohexanediamine	0.08	-3.61	2.11E-02	1.38	down
(2S)-4-Oxo-2-phenyl-3,4-dihydro-2H-chromen-7-yl beta-D-glucopyranoside	2.23	1.16	2.11E-02	1.22	up
D-Saccharic acid	2.29	1.20	2.17E-02	1.29	up
2,6-Xylidine	1.74	0.80	2.19E-02	1.40	up
L-Adrenaline	1.84	0.88	2.21E-02	1.26	up
Papaverine	0.65	-0.62	2.22E-02	1.37	down
Guaiacol	2.08	1.06	2.26E-02	1.21	up
1,3-Dimethyluric acid	3.22	1.69	2.30E-02	1.19	up
Ibuprofen metabolite B	58.87	5.88	2.40E-02	1.40	up
2,3,4-Trihydroxybenzoic acid	0.37	-1.42	2.45E-02	1.28	down
5-acetyl-2,6-dimethyl-1,2,3,4-tetrahydropyridin-4-one	1.58	0.66	2.51E-02	1.30	up
Cinnamyl alcohol	1.74	0.80	2.57E-02	1.21	up
N1-(4-chlorophenyl)-3-(1H-pyrrol-1-ylmethyl)piperidine-1-carboxamide	1.57	0.65	2.69E-02	1.15	up
3,4-Dihydroxybenzaldehyde	1.60	0.68	2.74E-02	1.10	up
4-decyl-3-hydroxy-5-oxooxolane-2,3-dicarboxylic acid	3.42	1.77	2.77E-02	1.20	up
methyl 4-oxo-4H-benzo[4,5]imidazo[2,1-b][1,3]thiazine-2-carboxylate	66.14	6.05	2.78E-02	1.19	up
2-(1-adamantyl)-1-morpholinoethan-1-one	2.03	1.02	2.91E-02	1.11	up
3-[4-methyl-1-(2-methylpropanoyl)-3-oxocyclohexyl]butanoic acid	1.53	0.61	2.93E-02	1.08	up
2-(4-methoxyphenyl)hydrazine-1-carbothioamide	2.17	1.12	2.96E-02	1.11	up
Alanyltyrosine	2.33	1.22	3.05E-02	1.18	up
Morphine-3-glucuronide	3.19	1.68	3.17E-02	1.13	up
Cathine	7.14	2.84	3.70E-02	1.50	up
Chlorogenic Acid Methyl Ester	1.93	0.95	3.81E-02	1.13	up
P-Coumaroyl Agmatine	1.70	0.76	4.15E-02	1.26	up
D-(+)-Arabitol	2.46	1.30	4.31E-02	1.19	up
3-Methylamino-L-alanine	4.36	2.12	4.31E-02	1.26	up
methyl 6-{[4-(trifluoromethyl)anilino]carbonyl}nicotinate	1.78	0.83	4.40E-02	1.06	up
Pyridoxal 5'-Phosphate	0.52	-0.96	4.41E-02	1.34	down
3,5-Dihydroxyphenylglycine	1.74	0.80	4.50E-02	1.10	up

Table S5 Differential metabolites in AGA vs HUA.

Metabolite	FC	log2FC	P	VIP	Up.Down
Palmitic Acid	9.76	3.29	1.41E-07	2.35	up
trans-3-Hydroxycotinine	25.28	4.66	1.89E-07	2.25	up
Oxoamide	12.13	3.60	2.39E-07	2.21	up
N1-[2-oxo-6-(1H-pyrrol-1-yl)-2H-chromen-3-yl]acetamide	18.39	4.20	3.08E-07	2.33	up
Oleamide	3.02	1.59	5.07E-07	2.24	up
Cotinine N-oxide	4.87	2.28	8.33E-07	2.08	up
3-Methylindole	4.73	2.24	1.30E-06	2.27	up
Cotinine	5.98	2.58	1.88E-06	2.01	up
Arachidonoyl amide	3.29	1.72	2.64E-06	2.22	up
L-Homocitrulline	0.38	-1.41	2.79E-06	3.18	down
Gamma-Glu-Leu	2.45	1.29	5.68E-06	2.89	up
Nicotine	10.96	3.45	7.36E-06	2.31	up
Xanthohumol	6.94	2.79	1.04E-05	2.16	up
2-Isopropylmalic acid	1.70	0.76	2.35E-05	2.16	up
1-butyl-2-methyl-4-nitro-1H-imidazole	0.36	-1.48	3.17E-05	2.70	down
2-(4-chlorophenyl)-2-oxoethyl 2,6-bis(trifluoromethyl)benzoate	32.55	5.02	3.55E-05	2.33	up
2-imino-8-methoxy-2H-chromene-3-carbonitrile	6.65	2.73	6.50E-05	1.89	up
gamma-Glutamylleucine	1.60	0.68	1.22E-04	2.30	up
1-(4-methyl-2-morpholino-1,3-thiazol-5-yl)ethan-1-one	3.04	1.60	1.32E-04	2.22	up
Xanthine	4.67	2.22	1.43E-04	2.05	up
2-Amino-1,3,4-octadecanetriol	5.47	2.45	1.68E-04	1.70	up
N1-(4-chlorophenyl)-2-cyano-4,4-dimethyl-3-oxopentanamide	0.61	-0.70	1.69E-04	1.82	down
2,6-Dihydroxypurine	2.01	1.01	1.76E-04	2.04	up
PMK	0.64	-0.64	2.48E-04	2.87	down
Xanthosine	3.04	1.61	2.76E-04	1.92	up
1-Methylnicotinamide	0.50	-0.99	4.05E-04	1.83	down
5-ethoxy-2-[(2,3,4,5,6-pentafluorobenzyl)thio]-1H-benzo[d]imidazole	109.14	6.77	4.90E-04	2.01	up
Cyclohexaneacetic acid	1.69	0.76	5.35E-04	2.20	up
N6-Succinyl Adenosine	1.56	0.64	5.69E-04	1.09	up
methyl 3,4,5-trihydroxycyclohex-1-ene-1-carboxylate	5.04	2.33	6.81E-04	1.63	up
N-Isovalerylglycine	1.64	0.71	7.14E-04	2.01	up
2,4-Dihydroxybenzoic acid	2.41	1.27	7.25E-04	1.72	up
10-Hydroxydecanoic acid	2.29	1.19	7.93E-04	1.80	up
2-(benzylthio)-4-[2-(3-methylbenzo[b]thiophen-2-yl)vinyl]pyrimidine	21.51	4.43	9.28E-04	1.95	up
L-Kynurenine	2.22	1.15	9.50E-04	2.04	up
Febuxostat	342.81	8.42	1.01E-03	1.68	up

5-[3-(2-Chloro-4-fluorobenzyl)-1,2,4-oxadiazol-5-yl]-3-pyrrolidinol	3.31	1.73	1.50E-03	1.91	up
Pantothenic acid	2.14	1.10	1.70E-03	2.29	up
4-Methyl-2-Oxopentanoic Acid	1.64	0.71	1.73E-03	1.84	up
4-oxo-4,5,6,7-tetrahydrobenzo[b]furan-3-carboxylic acid	2.75	1.46	1.95E-03	1.84	up
(+)-ar-Turmerone	0.60	-0.74	2.02E-03	2.10	down
3-Methylhistidine	0.58	-0.79	2.04E-03	2.26	down
5-Aminovaleric acid	1.76	0.82	2.04E-03	2.45	up
α-Hydroxyhippuric acid	0.59	-0.75	2.05E-03	1.66	down
Caprylic acid	2.17	1.12	2.15E-03	1.57	up
3-morpholino-5,6-diphenylpyridazine-4-carbonitrile	0.59	-0.75	2.43E-03	1.64	down
Isoferulic acid	0.43	-1.23	2.59E-03	1.70	down
Octanedioic acid	1.55	0.63	2.64E-03	1.94	up
2-[(5-chloro-3-pyridyl)oxy]-5-(1H-pyrrol-1-yl)pyridine	2.00	1.00	2.65E-03	1.81	up
2-{2-[(1-methyl-1H-pyrazol-5-yl)amino]-2-oxoethoxy}acetic acid	2.91	1.54	2.71E-03	1.47	up
L(-)-Carnitine	3.28	1.71	2.94E-03	1.67	up
2,2-dimethyl-6,7-di[(4-nitrobenzyl)oxy]chroman-4-one	0.44	-1.18	3.01E-03	1.82	down
2-(piperidinomethylidene)malononitrile	0.53	-0.92	3.08E-03	2.14	down
4-Acetamidobutyric Acid	0.35	-1.51	3.30E-03	3.05	down
Heptanoic acid	2.05	1.04	3.63E-03	1.68	up
Asp-Phe methyl ester	1.71	0.78	3.97E-03	2.05	up
4-methyl-5-oxo-2-pentyl-2,5-dihydrofuran-3-carboxylic acid	2.36	1.24	4.06E-03	1.72	up
3-Methoxytyramine	2.89	1.53	4.37E-03	1.56	up
2-[6-(1H-benzo[d]imidazol-2-yl)-2-pyridyl]-1H-benzo[d]imidazole	3.52	1.82	4.67E-03	1.57	up
Phenylacetylglutamine	0.65	-0.63	5.10E-03	1.74	down
6-benzyl-4-oxo-1,4-dihydropyridine-3-carboxamide	1.52	0.61	5.41E-03	1.47	up
Dl-Threitol	3.20	1.68	5.46E-03	1.31	up
3-[4-methyl-1-(2-methylpropanoyl)-3-oxocyclohexyl]butanoic acid	2.02	1.01	6.19E-03	1.76	up
5-Aminopentanoate	1.63	0.70	6.38E-03	2.11	up
L-Tyrosine	1.54	0.62	6.48E-03	1.79	up
2-Hydroxyvaleric acid	1.52	0.60	6.70E-03	1.55	up
Propionylcarnitine	3.48	1.80	6.95E-03	1.44	up
N-Acetyl-D-tryptophan	0.58	-0.78	6.97E-03	1.90	down
4-Hydroxyisoleucine	5.35	2.42	7.39E-03	1.60	up
L-Tryptophan	2.22	1.15	7.89E-03	2.00	up
N1-(4-chlorophenyl)-3-(1H-pyrrol-1-ylmethyl)piperidine-1-carboxamide	1.87	0.91	7.96E-03	1.60	up

Acetylcysteine	2.33	1.22	8.24E-03	1.45	up
2-Oxindole	0.34	-1.55	8.69E-03	2.19	down
Valproic acid	2.05	1.03	8.74E-03	1.66	up
Alanyltyrosine	3.04	1.61	9.17E-03	1.59	up
Taurine	2.07	1.05	9.66E-03	1.48	up
Melatonin	2.14	1.10	9.82E-03	1.24	up
3-(tert-butyl)-1-methyl-4,5-dihydro-1H-pyrazol-5-one	1.66	0.73	9.84E-03	1.31	up
N-Phenylacetylglutamine	0.62	-0.68	9.87E-03	1.87	down
DL-Carnitine	4.26	2.09	9.88E-03	1.42	up
ethyl 1-(3-nitro-2-thienyl)piperidine-4-carboxylate	6.21	2.64	9.98E-03	1.63	up
Nonanoic acid	1.85	0.89	1.00E-02	1.55	up
Guanidineacetic acid	0.59	-0.75	1.10E-02	1.72	down
3-Methylhistamine	0.57	-0.82	1.21E-02	1.92	down
Histamine	0.37	-1.45	1.25E-02	2.18	down
4,4'-dimethoxy[1,1'-biphenyl]-2-carbonitrile	7.50	2.91	1.28E-02	1.36	up
β-Estradiol-17β-glucuronide	2.39	1.26	1.40E-02	1.43	up
N-Formylkynurenine	3.38	1.76	1.43E-02	1.38	up
Acetaminophen glucuronide	59.67	5.90	1.46E-02	1.32	up
Orotic Acid	1.95	0.96	1.46E-02	1.36	up
1-(6-methyl-3-pyridyl)ethan-1-one O1-ethyloxime hydrochloride	2.54	1.35	1.47E-02	1.34	up
3-benzyl-1-butyl-4-hydroxy-1,2-dihydroquinolin-2-one	0.60	-0.75	1.53E-02	1.67	down
3-Hydroxyvaleric acid	1.55	0.63	1.56E-02	1.92	up
methyl 6-{[4-(trifluoromethyl)anilino]carbonyl}nicotinate	1.96	0.97	1.64E-02	1.31	up
Thromboxane B1	0.64	-0.65	1.70E-02	1.71	down
Propionyl-L-carnitine	1.57	0.65	1.72E-02	1.38	up
N-(5-Aminopentyl)acetamide	0.51	-0.96	1.74E-02	1.81	down
Isorhapontigenin	0.62	-0.69	1.79E-02	1.36	down
2,3,4-Trihydroxybenzoic acid	0.43	-1.21	1.80E-02	1.50	down
methyl 4-oxo-4H-benzo[4,5]imidazo[2,1-b][1,3]thiazine-2-carboxylate	71.54	6.16	1.82E-02	1.23	up
4-(tert-butyl)phenyl 3,5-dimethylisoxazole-4-carboxylate	0.60	-0.74	1.89E-02	1.37	down
2-oxopiperidine-3-carbohydrazide	0.39	-1.37	1.96E-02	1.98	down
Ala-trp	1.57	0.65	2.14E-02	1.78	up
16α-Hydroxyestrone	1.81	0.85	2.17E-02	1.62	up
Acetyl-L-carnitine	3.21	1.68	2.20E-02	1.28	up
(2S)-4-Oxo-2-phenyl-3,4-dihydro-2H-chromen-7-yl beta-D-glucopyranoside	2.62	1.39	2.34E-02	1.45	up
7-(2-hydroxypropan-2-yl)-1,4a-dimethyl-decahydronaphthalen-1-ol	8.14	3.02	2.49E-02	1.32	up
3-(dimethylamino)-2-[3-	1.63	0.70	2.53E-02	1.68	up

(trifluoromethyl)phenyl]acrylonitrile					
Valdecoxib	5.71	2.51	2.54E-02	1.19	up
All trans-Retinal	2.70	1.43	2.54E-02	1.30	up
Cinnamyl alcohol	1.99	0.99	2.58E-02	1.36	up
L-Carnitine	2.62	1.39	2.65E-02	1.18	up
Benzyl 6-O-beta-D-glucopyranosyl-beta-D-glucopyranoside	1.70	0.77	2.66E-02	1.35	up
7-Ketocholesterol	1.63	0.71	2.67E-02	1.22	up
2-[2-oxo-2-(2-pyridylamino)ethoxy]acetic acid	0.40	-1.31	2.80E-02	1.82	down
(2E)-3-phenyl-N-(2-phenylethyl)prop-2-enamide	0.66	-0.60	2.83E-02	1.61	down
Tyrosylalanine	1.53	0.62	2.86E-02	1.33	up
3-Methylamino-L-alanine	4.52	2.18	2.99E-02	1.28	up
2-phenyl-2,4,6,7-tetrahydrothiino[4,3-c]pyrazol-3-ol	4.38	2.13	3.03E-02	1.09	up
Estrone sulfate	1.53	0.61	3.05E-02	1.36	up
2-(2-acetyl-3,5-dihydroxyphenyl)acetic acid	0.53	-0.92	3.06E-02	1.54	down
Phenylglyoxylic acid	0.57	-0.82	3.23E-02	1.38	down
YNH	2.24	1.17	3.27E-02	1.20	up
Creatine	0.35	-1.52	3.31E-02	1.77	down
3-Methylhistidine	0.67	-0.59	3.32E-02	1.53	down
N-(1-benzylpiperidin-4-yl)-5-methoxy-1H-indole-2-carboxamide	19.51	4.29	3.35E-02	1.07	up
Sodium cholate	0.29	-1.77	3.39E-02	2.27	down
Acetildenafil	3.52	1.82	3.52E-02	1.38	up
Tyrosol	1.96	0.97	3.56E-02	1.13	up
Capric acid	1.93	0.95	3.58E-02	1.36	up
Boc-beta-cyano-L-alanine	1.58	0.66	3.59E-02	1.53	up
Nicotinate ribonucleoside	1.57	0.65	3.60E-02	1.12	up
Phellamurin	6.56	2.71	3.60E-02	1.53	up
Spermidine	1.83	0.87	3.68E-02	1.34	up
2-phenyl[1,3]oxazolo[4,5-c]quinolin-4(5H)-one	0.31	-1.68	3.71E-02	1.70	down
Cetirizine N-oxide	4.87	2.28	3.77E-02	1.30	up
5-phenyl-2,3-dihydro-1H-1,4-benzodiazepin-2-one	2.63	1.40	3.83E-02	1.19	up
5-(benzyloxy)-2-(hydroxymethyl)-1,4-dihydropyridin-4-one	2.10	1.07	3.89E-02	1.23	up
Guanine	1.54	0.62	4.02E-02	1.11	up
CAR 7:0	2.05	1.04	4.09E-02	1.14	up
N-Acetyl-DL-phenylalanine	1.55	0.63	4.27E-02	1.41	up
Cystathionine	2.32	1.22	4.36E-02	1.10	up
1-hydroxy-1-(4-methoxyphenyl)propan-2-yl 4-methoxybenzoate	0.63	-0.66	4.65E-02	1.21	down
Choline	1.50	0.59	4.78E-02	1.25	up
Undecanedioic acid	1.74	0.80	4.87E-02	1.18	up
N-Acetylcysteine	1.50	0.59	4.94E-02	1.28	up
N-Acetyl-L-tyrosine	1.51	0.59	4.94E-02	1.32	up

Table S6 Pathways significantly enriched in differential metabolite pathway analysis among HUA vs control, AGA vs control, and AGA vs HUA.

Pathway	Enriched metabolites	P	FDR	Impact
Caffeine metabolism	1-Methylxanthine, 7-Methylxanthine, Caffeine, 1-Methyluric acid, 1,7-Dimethyluric acid	7.92E-06	0.00	0.31
Arginine and proline metabolism	Guanidinoacetate, Creatine, Spermidine, L-Glutamate, 4-Acetamidobutanoate	6.08E-03	0.24	0.14
Nicotinate and nicotinamide metabolism	Nicotinate D-ribonucleoside, Nicotinamide, 1-Methylnicotinamide	1.23E-02	0.30	0.33
Histidine metabolism	L-Glutamate, Histamine, 3-Methylhistidine	1.48E-02	0.30	0.19
Glycine, serine and threonine metabolism	Choline, Guanidinoacetate, L-Cystathionine, Creatine	2.27E-02	0.36	0.03
Phenylalanine metabolism	Phenylacetaldehyde, L-Tyrosine	2.73E-02	0.36	0.14
Tyrosine metabolism	L-Adrenaline, 3-Methoxytyramine, L-Tyrosine, Tyramine	4.97E-02	0.57	0.19
